# Age‐related remodelling of the blood immunological portrait and the local tumor immune response in patients with luminal breast cancer

**DOI:** 10.1002/cti2.1184

**Published:** 2020-10-03

**Authors:** Lieze Berben, Giuseppe Floris, Cindy Kenis, Bruna Dalmasso, Ann Smeets, Hanne Vos, Patrick Neven, Asier Antoranz Martinez, Annouschka Laenen, Hans Wildiers, Sigrid Hatse

**Affiliations:** ^1^ Laboratory of Experimental Oncology Department of Oncology KU Leuven Leuven Belgium; ^2^ Department of Pathology University Hospitals Leuven Leuven Belgium; ^3^ Laboratory of Translational Cell and Tissue Research Department of Imaging and Pathology KU Leuven Leuven Belgium; ^4^ Department of General Medical Oncology and Geriatric Medicine University Hospitals Leuven Leuven Belgium; ^5^ Genetis of Rare Cancers Department of Internal Medicine and Medical Specialties (DiMI) University of Genoa and IRCCS Ospedale Policlinico San Martino Genoa Italy; ^6^ Department of Surgical Oncology KU Leuven, University Hospitals Leuven Leuven Belgium; ^7^ Department of Gynaecology and Obstetrics University Hospitals Leuven Leuven Belgium; ^8^ Interuniversity Centre for Biostatistics and Statistical Bioinformatics Leuven Belgium; ^9^ Department of General Medical Oncology University Hospitals Leuven Leuven Belgium

**Keywords:** ageing, biomarkers, breast cancer, clinical frailty, tumor immune infiltrate

## Abstract

**Objectives:**

Aging is associated with altered immune function and chronic low‐grade inflammation, referred to as immunosenescence. As breast cancer is an age‐related disease, the impact of aging on tumor immune responses may have important consequences. However, effects of immunosenescence on breast tumor immune infiltration remain largely unknown.

**Methods:**

This exploratory study investigated a broad panel of immune/senescence markers in peripheral blood and in the tumor microenvironment of young, middle‐aged and old patients diagnosed with early invasive luminal (hormone‐sensitive, HER2‐negative) breast cancer. In the old group, G8‐scores were computed as a correlate for clinical frailty.

**Results:**

Significant age‐related changes in plasma levels of several inflammatory mediators (IL‐1α, IP‐10, IL‐8, MCP‐1, CRP), immune checkpoint markers (Gal‐9, sCD25, TIM‐3, PD‐L1), IGF‐1 and circulating miRs (miR‐18a, miR‐19b, miR‐20, miR‐155, miR‐195 and miR‐326) were observed. Shifts were observed in distinct peripheral blood mononuclear cell populations, particularly naive CD8^+^ T‐cells. At the tumor level, aging was associated with lower total lymphocytic infiltration, together with decreased abundance of several immune cell markers, especially CD8. The relative fractions of cell subsets in the immune infiltrate were also altered. Clinical frailty was associated with higher frequencies of exhausted/senescent (CD27^−^CD28^−^ and/or CD57^+^) terminally differentiated CD8^+^ cells in the blood and with increased tumor infiltration by FOXP3^+^ cells.

**Conclusion:**

Aging and frailty are associated with profound changes of the blood and tumor immune profile in luminal breast cancer, pointing to a different interplay between tumor cells, immune cells and inflammatory mediators at higher age.

## Graphical abstract

In this study, apart from age‐related changes in the immune profile of patients with luminal breast cancer, remarkable frailty‐related changes within the subgroup of older patients were observed. Our data support age‐dependent remodelling of both systemic immunity features and anti‐tumor immune responses.

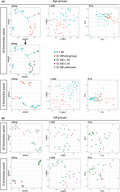

## Introduction

Breast cancer (BC), like all epithelial cancers, shows increased incidence with age. As the general population is ageing, the number of older patients with BC cancer is dramatically rising.^1^ Additionally, ageing might also impact BC biology: compared to younger patients, older women often develop less aggressive tumors that are mostly oestrogen receptor (ER)‐positive, lack over‐expression of human epidermal growth factor receptor‐2 (HER2) and have lower proliferation rates (luminal A‐like tumors). Contrarily, older patients often present with larger and more advanced stage tumors at diagnosis.

Lifelong exposure to endogenous and exogenous factors can induce progressive oxidative stress and DNA damage, eventually resulting in cell transformation and tumor initiation. Furthermore, ageing is associated with accumulation of metabolically active senescent cells, exhibiting a senescence‐associated secretory phenotype (SASP). SASP includes various inflammatory mediators that may promote tumor growth. Lastly, ageing induces a progressive decay of immune functioning, which may result in insufficient immune responses against a developing tumor.^2^, ^3^

Decreased adaptive immunity and increased low‐grade chronic inflammation in older people have been defined as ‘immunosenescence’ and ‘inflammaging’.^3^ Ageing strongly affects the adaptive immune system, reflected by shifts in abundance and functioning of several adaptive immune cell subsets. Decreased numbers of naive peripheral blood T cells and B cells and increases in memory cells and regulatory T cells (Tregs) have been reported.^3^, ^4^, ^5^, ^6^ T‐cell function is impaired since T cells become anergic and T‐cell receptor diversity declines. Compared to the CD4 compartment, the CD8 compartment appears to be more affected by age.^3^, ^5^, ^7^ Also, T‐cell *p16^INK4a^* expression has been described as a hallmark of T‐cell senescence.^8^ The innate immune system is reshaped as well. Chemotaxis and phagocytosis are reduced in neutrophils and macrophages. The latter produce more inflammatory cytokines, natural killer (NK) cells produce less cytokines, and their cytolytic potential decreases.^3^, ^7^, ^9^ In plasma, a gradual increase in pro‐inflammatory cytokines and chemokines has been observed, concomitant with a decrease in anti‐inflammatory mediators.^3^, ^10^ Additionally, several microRNAs (miRs) may be interesting immunosenescence markers. Over the past years, numerous miRs were reported to be involved in various immunological processes such as T‐ and B‐cell proliferation, differentiation and activation.^11^, ^12^, ^13^, ^14^

Breast cancer has long been considered as non‐immunogenic, yet many recent studies have demonstrated that the tumor immune infiltrate actually is of considerable clinical importance with respect to prognosis and outcome, most particularly for triple‐negative and HER2‐positive disease.^15^ Recent publications clearly show tumor‐infiltrating lymphocytes (TILs) in luminal BC^16^; however, the immune system’s role in hormone‐sensitive (luminal) BC is less established and impact of age has hardly been studied. Therefore, we performed an in‐depth analysis of the immunological profile, both in tumor and blood, in luminal BC patients from different age and frailty categories.

## Results

### Patient and tumor characteristics

Between March 2014 and November 2015, 65 patients who fulfilled the inclusion criteria were included in this study: 15 patients aged 35–45 years (young group); 19 patients aged 55–65 years (middle group) and 31 patients older than 70 years (old group). A sufficient amount of tumor tissue could be collected from 62 out of the 65 patients. In the old group, 19 patients had a ‘normal’ geriatric 8 (G8) score higher than 14 and 10 patients had a decreased G8 score that was equal to or lower than 14 (indicating increased risk for significant deficits when a full geriatric assessment is performed). Table 1 summarises the main characteristics of patients and tumors.

**Table 1:** Patient characteristics (age, G8 score for older patients) and tumor properties (histological subtype, grade, size and lymph node involvement)

Variable	Statistic	All	35–45 years	55–65 years	≥ 70 years
Age					
	*N* (%)	65	15	19	31
	Mean	63.4	40.1	60.4	76.3
	(Range)	(35.0; 89.0)	(35.0; 46.0)	(55.0; 65.0)	(70.0; 89.0)
G8 score					
	*N*	29			29
	Mean	15.2			15.2
	(Range)	(12.0; 17.0)			(12.0; 17.0)
Tumor histological Subtype					
Ductal (IDA)	*n*/*N* (%)	54/65 (83.1)	15/15 (100.0)	13/19 (68.4)	26/31 (83.9)
Lobular (ILA)	*n*/*N* (%)	5/65 (7.7)	0/15 (0.0)	3/19 (15.8)	2/31 (6.5)
Mixed ILA‐IDA	*n*/*N* (%)	2/65 (3.1)	0/15 (0.0)	1/19 (5.3)	1/31 (3.2)
Invasive solid papillary	*n*/*N* (%)	2/65 (3.1)	0/15 (0.0)	1/19 (5.3)	1/31 (3.2)
Micro‐papillary	*n*/*N* (%)	1/65 (1.5)	0/15 (0.0)	0/19 (0.0)	1/31 (3.2)
Mixed micro‐papillary and mucinous	*n*/*N* (%)	1/65 (1.5)	0/15 (0.0)	1/19 (5.3)	0/31 (0.0)
Tumor Grade					
Grade I	*n*/*N* (%)	1/65 (0.02)	0/15 (0.0)	0/19 (0.0)	1/31 (0.03)
Grade II	*n*/*N* (%)	40/65 (61.5)	9/15 (60.0)	10/19 (52.6)	21/31 (67.7)
Grade III	*n*/*N* (%)	24/65 (36.9)	6/15 (40.0)	9/19 (47.4)	9/31 (29.0)
Tumor size (mm)					
	*N*	65	15	19	31
	Mean	31.8	27.7	31.4	34.0
	(Range)	(10.0; 115.0)	(10.0; 60.0)	(15.0; 60.0)	(12.0; 115.0)
Node status					
pN0	*n*/*N* (%)	32/65 (49.2)	6/15 (40.0)	9/19 (47.4)	17/31 (54.8)
pN1	*n*/*N* (%)	29/65 (44.6)	8/15 (53.3)	9/19 (47.4)	12/31 (38.7)
pN2	*n*/*N* (%)	3/65 (4.6)	1/15 (6.7)	1/19 (5.3)	1/31 (3.2)
pN3	*n*/*N* (%)	1/65 (1.5)	0/15 (0.0)	0/19 (0.0)	1/31 (3.2)

The inclusion criteria were based on clinical estimate of the tumor size and on the grading based on the core needle biopsy. Enough tumor material could be collected for 62 out of the 65 patients. The table reports pathological tumor size and tumor grade measured on the resection specimen after surgery, explaining a few discrepancies between selection criteria and results on the surgical specimen reported here. For two patients in the old group, the G8 scores were not available.

This exploratory study generated enormous amounts of data. Only the most striking observations are presented in figures throughout this paper, while full data are available in Supplementary tables [Link], [Link], [Link], [Link], [Link], [Link], [Link].

### Analysis of immune/senescence markers in blood and association with age

**Figure 1:**
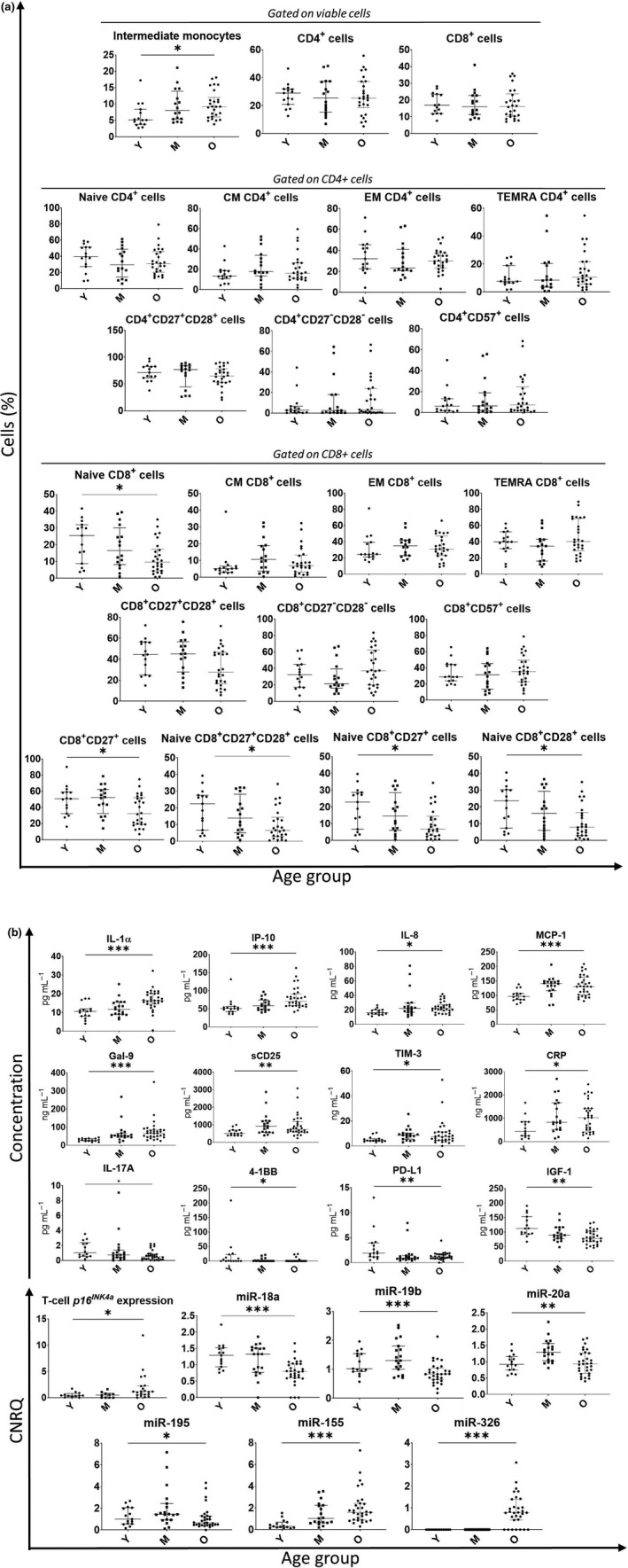
**(a)** PBMC subsets with significant age‐related variations are shown, as well as the important CD4 and CD8 T‐cell subpopulations (Naive, CM, EM, TEMRA) together with CD27/CD28 and CD57 expression on CD4^+^ and CD8^+^ T cells. **(b)** Blood immune/senescence plasma markers that showed significant difference between the three different age categories. Plasma markers measured via multiplex cytometric bead array technology (plasma cytokines, chemokines and immune checkpoint markers) or ELISA (IGF‐1) were run in duplicate. T‐cell *p16^INK4a^* and miRs expression were measured via RT‐PCR in triplicate. The 35–45 years category (*N* = 15) is represented with Y, the 55–65 years category (*N* = 19) is represented with M, and the ≥ 70 years (*N* = 31) category is represented with O. The median and IQR are shown in grey. The level of significance is represented with an *, with *: *P* ≤ 0.05; **: *P* ≤ 0.01 and ***: *P* ≤ 0.001. The *P*‐values were calculated via the Kruskal–Wallis test.

In the blood of the patients, multiple significant differences were observed between the three age groups, as summarised in Figure 1 and Supplementary table 1. Regarding PBMC subset profiling, no major differences between the age groups were noted among the principal cell populations (i.e. T cells, B cells, NK cells, dendritic cells, stem cells), except for a significant increase in the proportion of CD14^high^CD16^+^ intermediate monocytes (*P* = 0.019) with increasing age (Figure 1, Supplementary table 1). When looking deeper into T‐cell subsets, CD4^+^ subpopulations did not show any significant age‐related changes (Figure 1). Conversely, within the CD8^+^ pool, a significant decrease in cells expressing the costimulatory receptor CD27 (*P* = 0.036) was observed in the older age group. Moreover, the naive CD8^+^ compartment (i.e. CD8^+^ cells expressing both CD45RA and CD197/CCR7 and mostly CD28/CD27) showed to be highly affected by age: significant decreases were found not only for the total population of naive CD8^+^ cells (*P* = 0.035), but also for subsets of naive CD8^+^ cells expressing either CD27 (*P* = 0.018), CD28 (*P* = 0.031) or both (*P* = 0.021) (Supplementary table 1, Figure 1). With increasing age, significantly elevated plasma levels of several inflammatory cytokines [interleukin (IL)‐1α, *P* < 0.001) and chemokines (IP‐10, *P* < 0.001; IL‐8, *P* = 0.011 and monocyte chemoattractant protein 1 (MCP‐1), *P* = 0.001] were observed. Noteworthy, in our cohort, plasma levels of IL‐6 increased with ageing; however, this increase did not reach statistical significance. Moreover, no significant changes were observed in tumor necrosis factor alpha (TNFα) plasma levels (Supplementary table 1). Higher plasma levels at more advanced ages were also noted for different soluble mediators, the immune checkpoint markers galectin‐9 (Gal‐9) (*P* < 0.001), soluble CD25 (sCD25) (*P* = 0.008) and T‐cell immunoglobulin and mucin domain 3 (TIM‐3) (*P* = 0.017), as well as for the aspecific inflammation parameter C‐reactive protein (CRP) (*P* = 0.030). Contrary, significant age‐related decreases were seen for the inflammatory cytokine IL‐17A (*P* = 0.012), immune checkpoint marker 4‐1BB (*P* = 0.025) and programmed death‐ligand 1 (PD‐L1) (*P* = 0.008) and, most prominently, for the insulin‐like growth factor‐1 (IGF‐1) (*P* = 0.002). We noticed that, however, age‐related alterations did not always follow similar dynamics in function of time: whereas some markers progressively increased/decreased over the age groups (e.g. interferon gamma‐induced protein 10 (IP‐10), Gal‐9, CRP, IGF‐1), others reached a maximum/minimum in the middle age group and then remained more or less stable (e.g. IL‐8, MCP‐1, 4‐1BB) or even slightly dropped/raised again (e.g. sCD25, TIM‐3, PD‐L1). A significant increase of T‐cell *p16^INK4a^* expression with age (*P* = 0.014), especially in the oldest group, was also observed in our cohort. However, this marker could only be measured in 66% of patients because of the low RNA yield from the purified T cells. Finally, we also studied the evolution of the plasma miR profile in function of age. Six miRs showed significantly different plasma expression levels between the age groups: miR‐18a (*P* < 0.001) was decreased in the oldest age group, while miR‐19b (*P* < 0.001), miR‐20a (*P* = 0.002) and miR‐195 (*P* = 0.034) first peaked in the 55–65 years group but then dropped again in the ≥70 years group. A marked progressive increase with age was seen for miR‐155 (*P* < 0.001). Noteworthy, miR‐326 could only be measured in the majority (77%) of patients from the ≥ 70 years group, while being totally undetectable in young and middle‐aged patients (Figure 1).

### Characterisation of the tumor immune infiltrate in the different age groups

**Figure 2:**
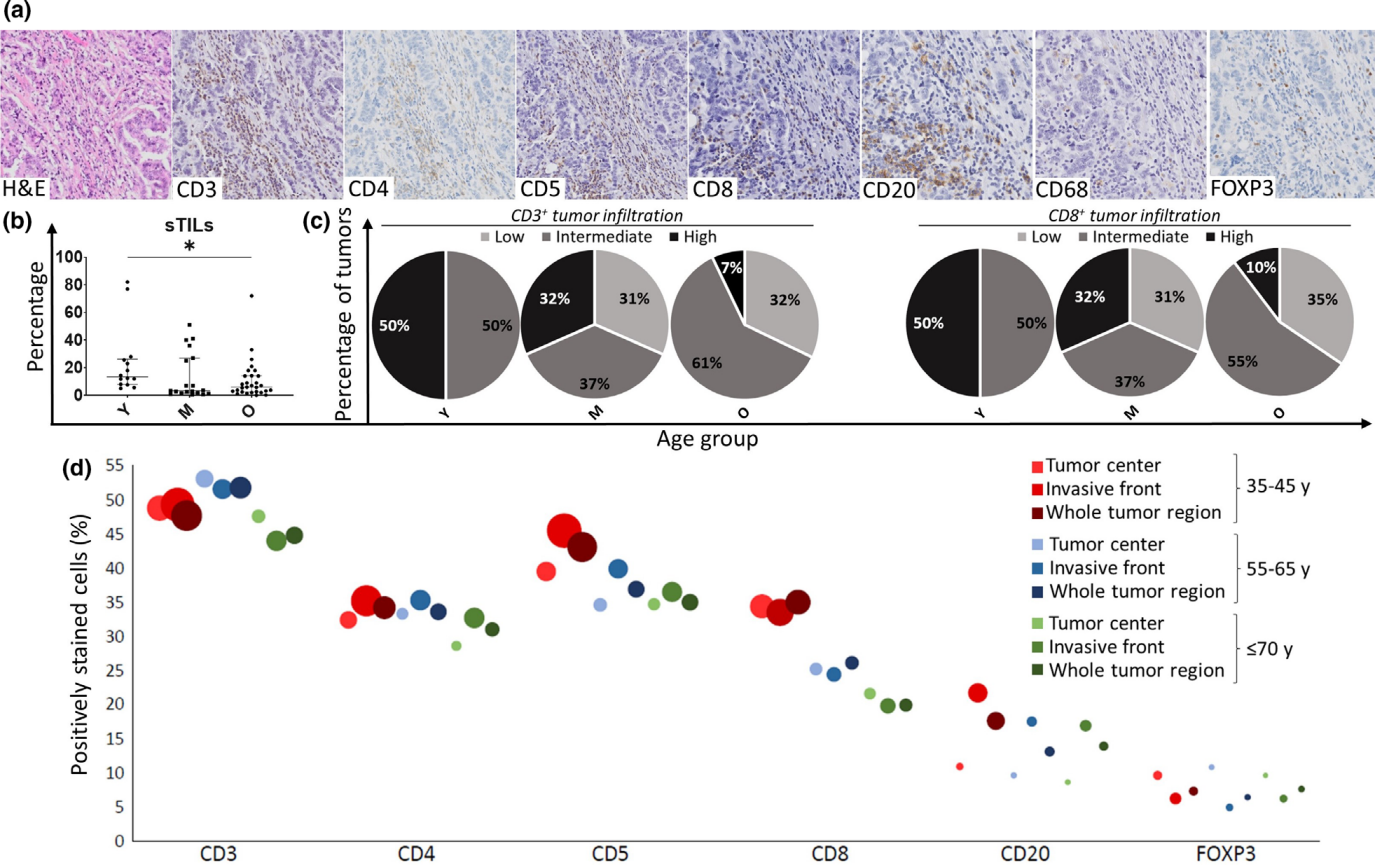
**(a)** Representative microphotographs of the 7 immunostainings: H&E, CD3, CD4, CD5, CD8, CD20, CD68 and FOXP3. The microphotographs were collected from the same tumor sample, which had 27.0% sTILs. **(b)** Stromal tumor‐infiltrating lymphocytes (sTILs) % shows a significant difference (*P* = 0.025) between the three different age categories: the 35–45 years ‘young’ (Y) category (*N* = 14), the 55–65 years ‘middle’ (M) category (*N* = 19) and the ≥ 70 years ‘old’ (O) category (*N* = 29). The median and IQR are shown. **(c)** The percentage of tumors with a low, intermediate or high CD3 and CD8 infiltration in the different age groups. The cut‐offs were based on the IQR of the density of CD3^+^ and CD8^+^ cells in the whole cohort. Low infiltration: minimum – lower quartile (lowest 25%), intermediate infiltration: lower quartile – upper quartile and high infiltration: upper quartile – maximum (highest 25%). **(d)** Tumor immune infiltration in the different tumor regions for the different age categories. The different immune cell markers (CD3, CD4, CD5, CD8, CD20 and FOXP3) are denoted on the *x*‐axis. Positively stained immune cells (%) can be found on the *y*‐axis. The area of the bubbles represents the density (absolute number per mm^2^) of the marker in that specific region. The 35–45 years age category is represented in red, the 55–65 years category in blue and the ≥ 70 years category in green. The tumor centre, invasive front and the whole tumor region are represented by increasing colour intensities. *P*‐values were calculated via the Kruskal–Wallis test.

For each immune cell marker, full data, including proportion and density of positively stained cells in different tumor zones (invasive front, tumor centre and whole tumor), are summarised per age category in Supplementary table 2 and in Figure 2 with representative microphotographs of each marker. Stromal tumor‐infiltrating lymphocytes (sTILs) % ranged between 0% and 82%. Yet, only 5 tumors showed ≥ 40% sTILs. The predominant TIL subset consisted of CD3^+^ T cells in all patients, but CD20^+^ B cells also represented a considerable fraction. The proportion of CD3^+^ T cells seemed to expand, while the fraction of B cells decreased with increasing tumor size. We also examined the spatial distribution of the immune subtypes within the tumor and noted that, compared to the tumor centre, TILs in the invasive front were significantly enriched in CD20^+^, cells which was also seen by König *et al*.,^17^ while FOXP3^+^ cells tended to invade much deeper into the tumor core. For the markers CD3, CD4, CD5 and CD8, spatial differences were less pronounced. With increasing age, various significant changes were observed regarding the tumor immune infiltrate. sTILs % (*P* = 0.025) decreased with increasing age; tumors from patients of the middle‐aged group surprisingly exhibited the lowest degree of lymphocytic infiltration (Figure 2). The pie charts (Figure 2) show the % of tumors with a low, intermediate, or high CD3 and CD8 infiltration in the different age groups, revealing a striking decrease of highly infiltrated tumors (both for CD3 and CD8) with increasing age. Comprehensive results for the particular cell subsets (i.e. proportion and density of positive cells in distinct tumor regions for each age group) are displayed in the bubble chart (Figure 2). The density of cells staining positive for the T‐cell markers CD3 and CD5 decreased in all tumor regions with increasing age [CD3: tumor centre (*P* = 0.007), invasive front (*P* = 0.019) and whole tumor (*P* = 0.005); CD5: tumor centre (*P* = 0.022), invasive front (*P* = 0.006) and whole tumor (*P* = 0.006)] (Supplementary table 2, Figure 2). Likewise, an age‐related decrease in the density of the cytotoxic T‐cell marker CD8 was seen in all tumor regions (tumor centre: *P* = 0.002, invasive front and whole tumor: *P* < 0.001). The B‐cell marker CD20 was less abundant in the invasive front (*P* = 0.042), and whole tumor (*P* = 0.031) in the older age groups, although the age effect appeared to be less pronounced than for the T‐cell markers CD3, CD5 and CD8. Furthermore, the proportion of the immune infiltrate also changed with ageing: proportions of lymphocytes staining positive for CD8^+^ were lower in all tumor regions (all *P* < 0.001) at older ages. Differently, no significant age‐related changes were observed for the markers CD4, FOXP3 and CD68 (Supplementary table 2, Figure 2). Besides the immune infiltrate’s quantity and composition in the tumor regions, spatial distribution of the immune markers within the tumor area was also investigated. We compared within each age group the mean proportion of positive cells between the different tumor regions. No significant interactions were observed between the age groups and tumor regions (invasive front and tumor centre), indicating that distribution patterns of the studied immune cells within the tumor did not significantly differ between the age groups (Supplementary table 3). Irrespective of age group, several immune subtypes were unequally distributed across the tumor zones: CD20^+^ cells predominantly resided in the invasive front, and FOXP3^+^ cells represented a higher proportion of immune cells in the tumor centre (Supplementary table 3).

### Association of blood immune/senescence markers with G8 score

**Figure 3:**
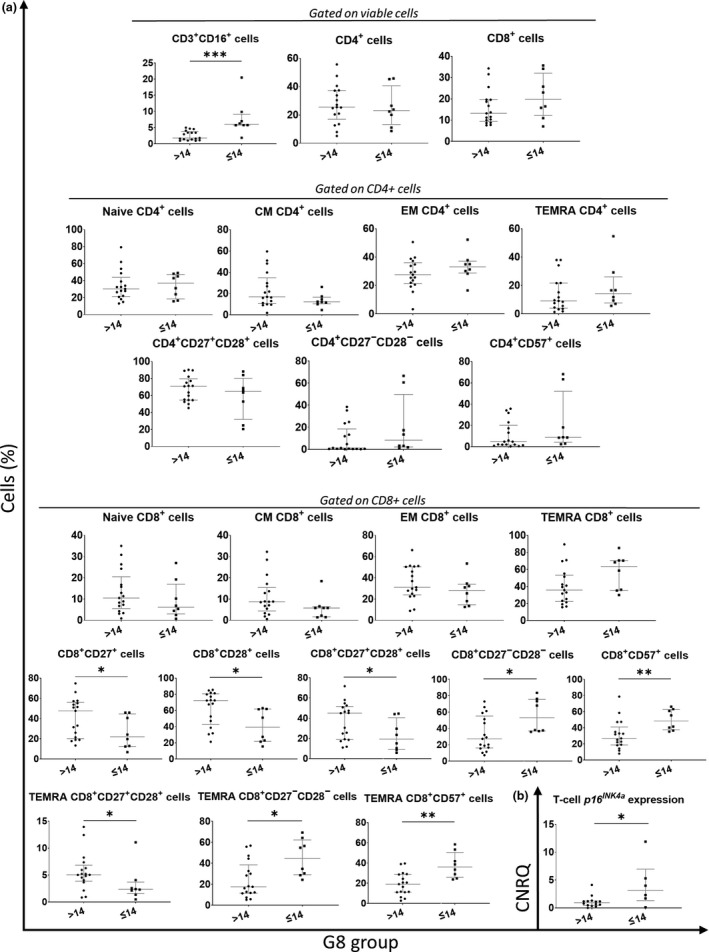
**(a)** PBMC subsets with significant variations between the G8 groups [G8 > 14: ‘fit’ (*N* = 19) and G8 ≤ 14: ‘frail’ (*N* = 10)] are shown, and biologically relevant CD4 and CD8 T‐cell subpopulations (Naive, CM, EM, TEMRA) together with CD27/CD28 and CD57 expression on CD4^+^ and CD8^+^ T‐cells. **(b)** T‐cell *p16^INK4a^* expression, showing significant difference between the two G8 groups. Plasma markers measured via multiplex cytometric bead array technology (plasma cytokines, chemokines and immune checkpoint markers) or ELISA (IGF‐1) were run in duplicate. T‐cell *p16^INK4a^* and miRNA expression were measured via RT‐PCR in triplicate. The median and IQR are shown in grey. Levels of significance: *: *P* ≤ 0.05; **: *P* ≤ 0.01 and ***: *P* ≤ 0.001. The *P*‐values were calculated via the Mann–Whitney *U* test.

Next, we compared the blood immune/senescence profile between the G8 subgroups (i.e. G8‐normal and G8‐decreased) of the ≥ 70 years age category, as summarised in Supplementary table 4, mean age of the groups was comparable. While none of the plasma protein biomarkers (including IL‐6, TNFα and CRP) nor miRs differed significantly between both G8 subgroups, the PBMC subset profiles revealed multiple significant G8‐dependent differences (Figure 3). Firstly, fractions of total CD8^+^ cells expressing CD27 or CD28 were significantly lower in more frail patients (*P* = 0.044 and *P* = 0.016, respectively) as compared to fit patients. The same also applied to double‐positive CD27^+^CD28^+^ cells (*P* = 0.029), whereas double‐negative CD27^−^CD28^−^ cells (mostly terminally differentiated cells) were concurrently increased within the CD8^+^ compartment of frailer patients (*P* = 0.013). Similar observations were made when looking specifically into the terminally differentiated effector memory re‐expressing CD45RA (TEMRA) CD8^+^ subpopulation: the fraction of cells co‐expressing CD27 and CD28 was again lower in the G8‐decreased than in the G8‐normal subgroup (*P* = 0.044), while, accordingly, the proportion of cells lacking both costimulatory receptors was higher in the frailer group (*P* = 0.013). Moreover, the proportion of cells expressing the senescence marker CD57 was significantly higher in frailer patients, both in the total CD8^+^ pool (*P* = 0.007) and in the TEMRA CD8^+^ subpopulation (*P* = 0.005). Additionally, the frequency of a peculiar subpopulation of CD3^+^CD16^+^ cells (NK‐like T cells) was significantly higher (*P* < 0.001) in the subgroup of frailer patients (G8 ≤ 14). Finally, higher T‐cell *p16^INK4a^* expression (*P* = 0.043) was also associated with lower G8 score and thus with frailer patients.

### G8‐dependent changes in the tumor immune infiltrate

**Figure 4:**
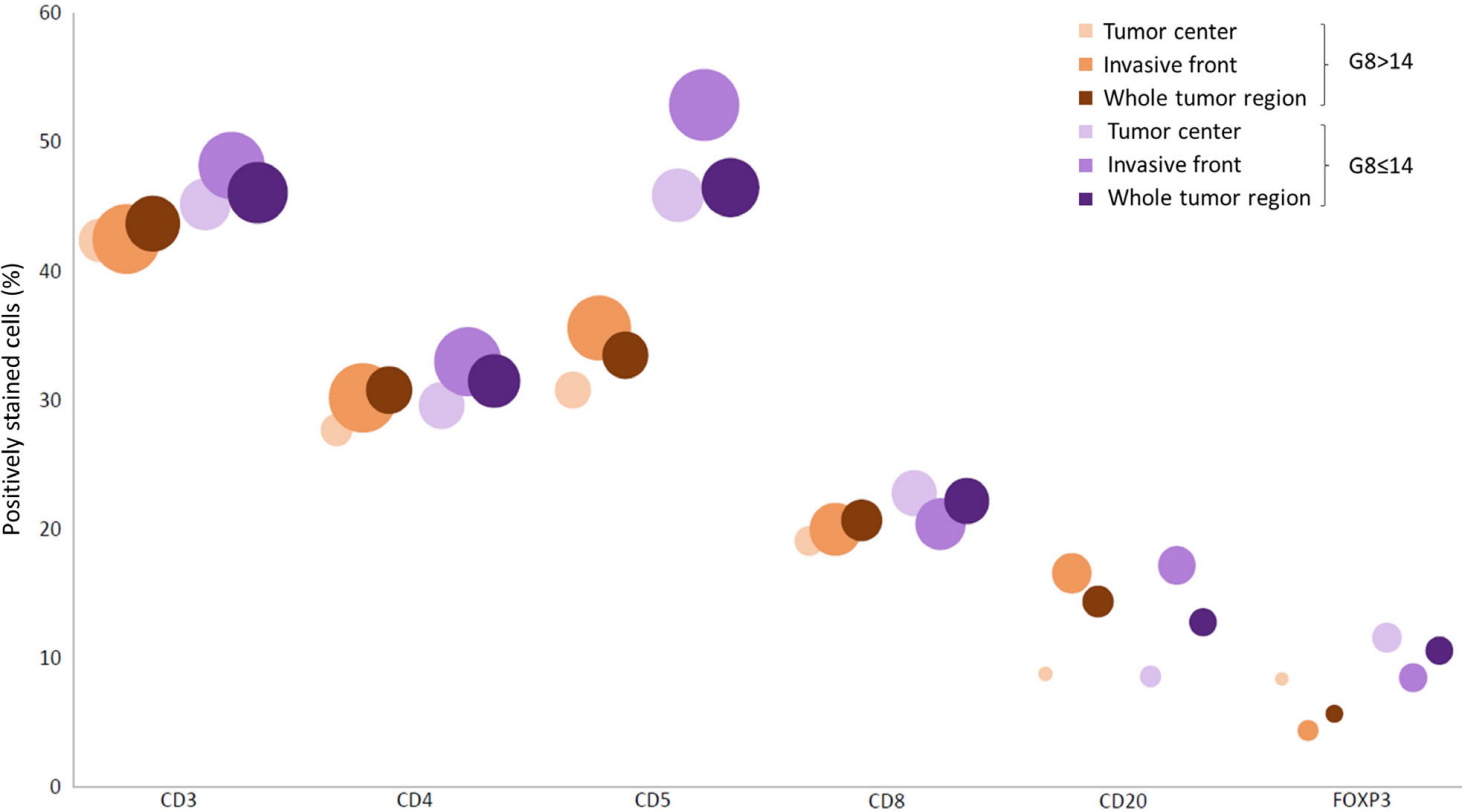
Bubble chart: tumor immune infiltration in the different tumor regions in the different G8 groups [G8 > 14: ‘fit’ (*N* = 19) and G8 ≤ 14: ‘frail’ (*N* = 10)]. The different immune cell markers (CD3, CD4, CD5, CD8, CD20 and FOXP3) are represented on the *x*‐axis. Positively stained immune cells (%) can be found on the *y*‐axis. The area of the bubbles represent the density of each marker in a specific region. The tumor centre is represented by the lightest shade, invasive front by the second lightest shade and the whole tumor region by the darkest colour. The G8 > 14 is represented in orange, the G8 ≤ 14 in purple.

Within the oldest (≥ 70 years) age category, we also examined the different tumor immune markers according to G8 score (Supplementary table 5, Figure 4). Noteworthy, tumor characteristics were comparable between the two groups. Significant changes were observed between G8‐normal and G8‐decreased subgroups with regard to FOXP3^+^ cell infiltration. In frailer patients (G8 ≤ 14), the proportion of FOXP3^+^ cells was higher in the invasive front (*P* = 0.015) and whole tumor (*P* = 0.007), as compared to fit patients (G8 > 14), and the density of FOXP3^+^ cells was also increased in the tumor centre (*P* = 0.037). Thus, breast tumors in frailer patients appeared to be more heavily infiltrated by immunosuppressive FOXP3^+^ cells. sTILs %, CD68 grade and infiltration of the other markers were not associated with the G8 score. Again, no significant interactions were observed between G8 groups and tumor regions (invasive front and tumor centre), indicating that spatial distribution of the studied immune cells within the tumor did not differ between the G8 groups (Supplementary table 3).

### Correlations between blood immune/senescence markers and tumor immune infiltrate

We also performed a detailed investigation of the relationships among and between blood and tumor immune biomarkers (Supplementary table 6). Firstly, sTILs % inversely correlated with the pro‐inflammatory plasma markers IL‐1α, IL‐6 and MCP‐1, the plasma immune checkpoint markers TIM‐3 and Gal‐9, with CRP and also with circulating Tregs. The strong negative correlation of MCP‐1 with global sTILs % was further substantiated by its inverse relationship with whole tumor density of CD3^+^, CD5^+^, CD8^+^ and FOXP3^+^ cells. The immune checkpoint marker TIM‐3, in addition to its negative association with sTILs %, also inversely correlated with CD68 staining grade and with whole tumor density of CD8^+^ and CD20^+^ cells, while IL‐1α only showed a significant inverse association with CD8. Secondly, several correlations were noted between specific tumor immune markers and PBMC subpopulations. Remarkably, circulating class‐switched memory B cells (CD27^+^IgD^−^) showed a strong inverse correlation with CD68 staining grade, while intermediate monocytes (CD14^++^CD16^+^) negatively correlated with CD3^+^ and CD5^+^ cell density in the tumor. Also, the density of CD8^+^ cells in the whole tumor was inversely correlated with the % Tregs in blood. CD8^+^ TEMRA cells expressing CD27 and/or CD28 showed multiple positive correlations with tumor infiltration by CD3^+^, CD4^+^, CD5^+^, CD8^+^ and CD20^+^ cells, whereas senescent CD8^+^ TEMRA (lacking both CD27 and CD28 and/or expressing CD57) clearly exhibited a positive association with infiltration of FOXP3^+^ cells in the tumor. Similar observations, albeit somewhat less pronounced, were made for the corresponding CD4^+^ TEMRA subsets. Lastly, several immune‐related circulating miRs also showed diverse correlations with different tumor immune infiltration markers. miR‐17 and especially miR‐19b appeared to strongly correlate with the proportion of CD3^+^ cells in the tumor infiltrate, while miR‐195 consistently showed positive associations with both proportion and density of CD3^+^, CD4^+^, CD5^+^, CD8^+^ and FOXP3^+^ cells, but not CD20^+^ cells. Other correlations between the blood immune/senescence markers and tumor immune infiltrate markers are not further discussed here because statistical significance was only moderate and biological relevance uncertain (Supplementary table 6). Additionally, solid correlations were found between breast tumor biology parameters (size, grade and nodal status) and the immune profile, mainly at tumor level (Supplementary results and Supplementary table 7).

### Age and G8 group prediction using a panel of blood and tumor biomarkers

**Figure 5:**
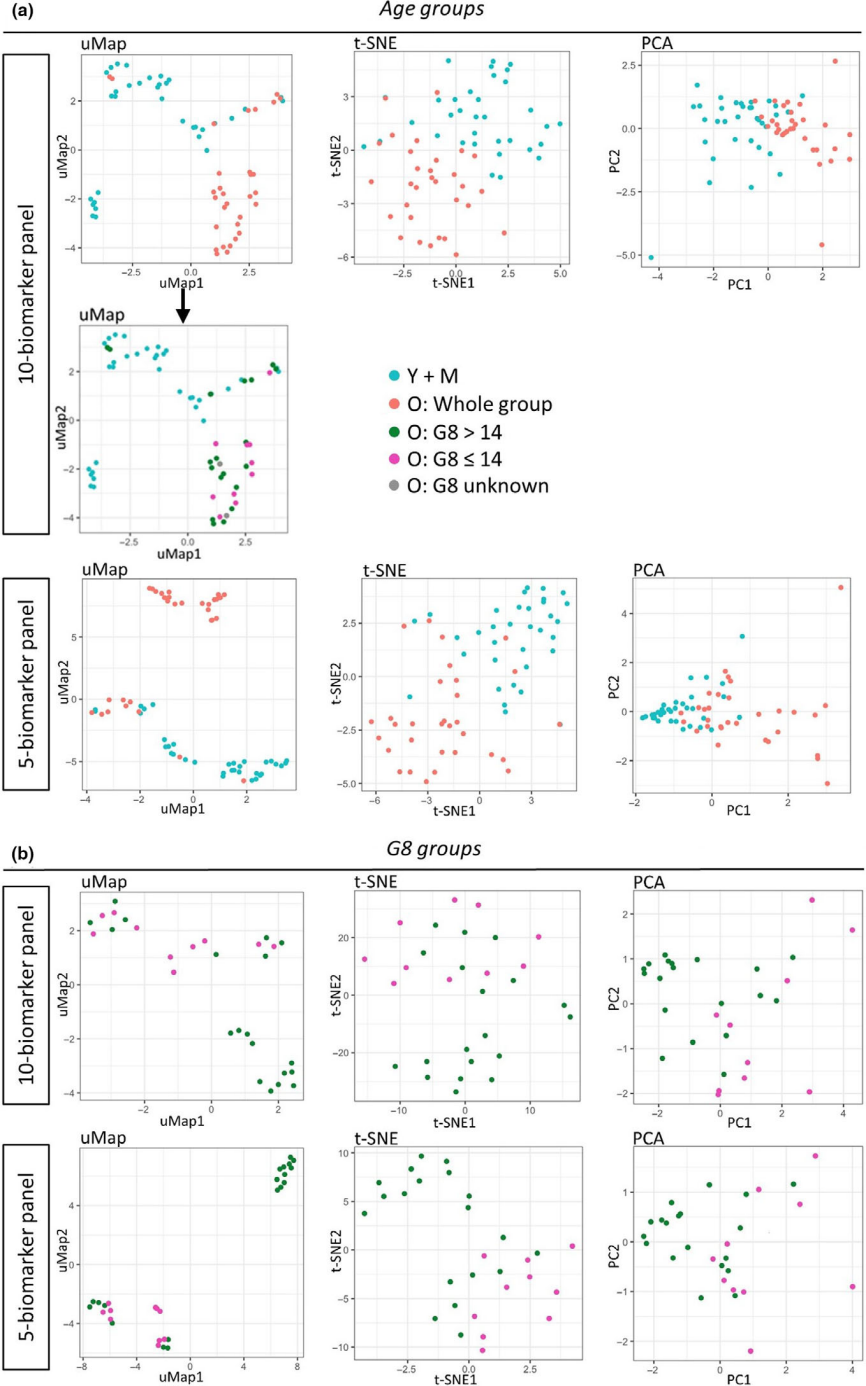
Dimensionality reduction plots. **(a)** A panel of 10 and 5 biomarkers was assembled to classify the patients in the old group (*N* = 31) or the younger age groups (*N* = 34). The 10 and 5 biomarkers were selected based on their statistical performance and biological relevance. uMap, t‐SNE and PCA projections are shown, with the younger age groups in blue (Y + M), the whole old group in red. The uMap projection of the 10‐biomarker age panel shows a distinct cluster of older patients. This cluster was studied in more detail in the plot below, showing ‘frail’ older patients (G8 ≤ 14) are shown in pink, ‘fit’ older patients (G8 > 14) in green and older patients with an unknown G8‐score in grey. **(b)** Another panel of 10 or 5 biomarkers was assembled to classify the patients in the ‘fit’ G8 > 14 group (*N* = 19) or the ‘frail’ G8 ≤ 14 group (*N* = 10). Again, the 10 or 5 biomarkers were selected based on their statistical performance and biological relevance. On the uMap, t‐SNE and PCA projections the ‘fit’ patients (G8 > 14) are shown in green and the ‘frail’ patients (G8 ≤ 14) in pink.

Additionally, we examined the ability of the blood immune/senescence and tumor immune infiltrate markers to predict a patient’s age and G8 group. For both age and G8, panels of 10 and 5 biomarkers were selected based on their individual statistical performance and biological relevance. The 50 statistically highest ranked blood immune/senescence and tumor immune infiltrate markers can be found in Table 2. The 10‐biomarker panel for age groups consisted of the blood immune/senescence markers: miR‐326, miR‐155, *p16^INK4a^*, IL‐17A, Gal‐9, IP‐10, IGF‐1 and naive CD8^+^CD27^+^CD28^+^ cells in combination with the tumor immune infiltrate markers: whole tumor density of CD8^+^ cells and tumor centre density of CD3^+^ cells. Using this 10‐biomarker panel, the old group could be separated quite well from the two younger groups by either dimensionality reduction tool (uMap, PCA or t‐SNE) (Figure 5). Moreover, the uMap shows a distinct cluster of older patients while a smaller group of older patients mix with the younger patients. Interestingly, all ‘frailer’ patients (G8 ≤ 14) except for one were contained within the distinct cluster of older patients and this observation was statistically significant (*P* = 0.018). For the G8 groups, the 10‐biomarker panel included the blood immune/senescence markers: miR‐9, *p16^INK4a^*, Gal‐9, CD3^+^CD16^+^, CD8^+^CD57^+^, CD8^+^CD27^−^CD28^−^, CD4^+^CD27^−^CD28^−^ cells and CD4^+^CD57^+^ cells together with the tumor immune infiltrate markers: whole tumor proportion and tumor centre density of FOXP3^+^ cells. The ‘frailer’ older patients (G8 ≤ 14) could be separated relatively well from the ‘fit’ older patients (G8 > 14) using the 10‐biomarker panel (Figure 5). The uMap clearly shows that most of the ‘fit’ patients are separated from the ‘frailer’ patients, while some of the ‘fit’ patients mix with the ‘frailer’ patients. Furthermore, we checked if the same or better separation could be achieved using 5‐biomarker panels. These consisted of miR‐326, miR‐155, *p16^INK4a^,* Gal‐9 and IP‐10 for age and *p16^INK4a^*, CD3^+^CD16^+^, CD8^+^CD57^+^, CD28^+^CD27^−^CD28^−^ and whole tumor proportion of FOXP3^+^ cells for the G8 groups. For both age and G8, the 5‐biomarker panel displayed an even better performance compared to the 10‐biomaker panel in the uMap, while performing comparably well in t‐SNE and PCA (Figure 5). Moreover, for age, a distinct cluster with most of the frailer patients could be observed with the 5‐biomarker panel.

**Table 2:** The 50 statistically highest ranked blood immune/senescence and tumor immune infiltrate markers to classify the patients in the age groups and the G8 groups

Position	Blood immune/senescence and tumor immune infiltrate markers	*N*	AUC	*P*‐value	log FC	AUC score	*P‐*value score	log FC score	Final score
Age									
1	miR‐326	65	0.110	< 0.001	10.00	4	1	1	3
2	*p16^INK4a^*	43	0.240	0.004	1.87	44	44	51	46
3	miR‐155	65	0.240	< 0.001	1.01	43	15	193	74
4	CD8^+^ cells – Whole tumor – Density	62	0.710	0.004	−1.45	129	45	109	103
5	miR‐18a	65	0.780	< 0.001	−0.63	27	7	394	114
6	miR‐19b	65	0.780	< 0.001	−0.62	28	9	407	118
7	CD3^+^ cells – Tumor centre – Density	61	0.700	0.007	−1.49	161	60	99	120
8	IL‐17A	65	0.690	0.010	−2.19	192	85	37	127
9	Gal‐9	65	0.240	< 0.001	0.61	42	14	419	129
10	CD8^+^ cells – Tumor centre – Density	62	0.690	0.010	−1.59	196	86	74	138
11	PBMC – Naive CD8^+^CD27^+^	57	0.710	0.007	−0.85	127	66	253	143
12	CD3^+^ cells – Whole tumor – Density	61	0.690	0.011	−1.29	195	101	142	158
13	PBMC – Naive CD8^+^CD27^+^CD28^+^	57	0.700	0.008	−0.84	160	72	256	162
14	CD8^+^ cells – Invasive front – Proportion	62	0.760	< 0.001	−0.48	45	19	577	172
15	CD8^+^ cells – Invasive front – Density	62	0.680	0.013	−1.21	216	112	152	174
16	CD8^+^ cells – Whole tumor – Proportion	62	0.760	0.001	−0.46	46	23	612	182
17	IP‐10	65	0.240	< 0.001	0.43	41	20	659	190
18	PBMC – Naive CD8^+^CD28^+^	57	0.690	0.013	−0.78	190	110	289	195
19	PBMC – Naive CD8^+^	57	0.690	0.013	−0.74	191	106	320	202
20	CD5^+^ cells – Whole tumor – Density	62	0.670	0.023	−1.18	264	149	156	208
21	CD5^+^ cells – Tumor centre – Density	62	0.660	0.033	−1.23	309	177	149	236
22	CD8^+^ cells – Tumor centre – Proportion	62	0.720	0.003	−0.40	101	38	734	244
23	CD5^+^ cells – Invasive front – Density	62	0.660	0.036	−1.00	308	197	198	253
24	4‐1BB	65	0.640	0.019	−2.42	427	134	31	255
25	PBMC – CD8^+^CD27^+^	57	0.700	0.010	−0.46	159	89	613	255
26	miR‐195	65	0.670	0.021	−0.64	262	138	384	262
27	IGF‐1	65	0.710	0.003	−0.37	128	37	805	275
28	miR‐9	65	0.640	0.022	−1.36	430	141	126	282
29	CD3^+^ cell – Invasive front – Density	61	0.650	0.043	−1.01	359	216	194	282
30	PBMC – CD8^+^CD27^+^CD28^+^	57	0.680	0.020	−0.44	214	135	649	303
31	CD20^+^ cells – Tumor centre – Density	62	0.640	0.064	−1.51	431	277	92	308
32	CD20^+^ cells – Whole tumor – Density	62	0.640	0.060	−1.39	432	269	120	313
33	PBMC – CD56^bright^CD16^−^ NK cells	57	0.660	0.040	−0.60	306	208	438	315
34	CD4^+^ cells – Whole tumor – Density	61	0.640	0.055	−1.07	434	249	179	324
35	FOXP3^+^ cells – Tumor centre – Density	61	0.640	0.066	−0.97	435	287	205	341
36	CD4^+^ cells – Tumor centre – Density	61	0.630	0.087	−1.19	523	358	155	390
37	CD3^+^ cells – Invasive front – Proportion	61	0.690	0.010	−0.21	193	93	1371	463
38	miR‐19a	65	0.640	0.049	−0.39	429	235	759	463
39	miR‐20a	65	0.660	0.029	−0.28	307	163	1081	465
40	CD20^+^ cells – Invasive front – Density	62	0.620	0.121	−0.94	624	481	212	485
41	miR‐125b	65	0.640	0.050	−0.35	428	237	864	489
42	CD3^+^ cells – Whole tumor – Proportion	61	0.680	0.018	−0.20	215	131	1425	497
43	FOXP3^+^ cells – Whole tumor – Density	61	0.620	0.114	−0.73	626	458	326	509
44	PBMC – EM CD8^+^CD27^+^	57	0.650	0.058	−0.28	356	257	1082	513
45	PBMC – Intermediate monocytes	57	0.350	0.059	0.28	358	262	1083	515
46	Total pDC	57	0.640	0.079	−0.25	425	333	1191	594
47	let‐7e	65	0.370	0.077	0.30	520	331	1016	597
48	PBMC – TEMRA CD8^+^CD27^−^CD28^−^	57	0.380	0.124	0.43	622	499	666	602
49	PBMC – CD8^+^CD27^−^CD28^−^	57	0.380	0.119	0.39	621	472	762	619
50	miR‐21	65	0.620	0.095	−0.33	623	389	917	638
G8									
1	PBMC – CD3^+^CD16^+^	25	0.940	< 0.001	−1.62	1	10	67	20
2	PBMC – TEMRA CD8^+^CD57^+^	25	0.860	0.003	−0.98	5	40	202	63
3	*p16^INK4a^*	20	0.800	0.041	−1.94	21	211	47	75
4	FOXP3^+^ cells – Whole tumor – Proportion	27	0.830	0.005	−0.80	11	50	274	87
5	PBMC – TEMRA CD8^+^CD27^−^CD28^−^	25	0.820	0.011	−0.90	16	98	225	89
6	PBMC – CD8^+^CD57^+^	25	0.850	0.005	−0.67	7	49	356	105
7	FOXP3^+^ cells – Invasive front – Proportion	27	0.800	0.012	−0.78	22	105	285	109
8	PBMC – CD8^+^CD27^−^CD28^−^	25	0.820	0.011	−0.75	15	99	310	110
9	FOXP3^+^ cells – Tumor centre – Density	27	0.750	0.035	−1.24	53	191	148	111
10	PBMC – CD8^+^CD28^+^	25	0.190	0.013	0.63	20	109	393	136
11	PBMC – CD8^+^CD27^+^CD28^+^	25	0.220	0.027	0.71	29	153	331	136
12	PBMC – EM CD4^+^CD57^+^	25	0.740	0.057	−1.07	70	255	176	143
13	PBMC – TEMRA CD8^+^CD27^+^CD28^+^	25	0.240	0.044	0.77	48	221	295	153
14	PBMC – TEMRA CD4^+^CD57^+^	25	0.730	0.076	−1.20	82	321	154	160
15	PBMC – CD8^+^CD27^+^	25	0.240	0.044	0.65	47	222	373	172
16	PBMC – CD4^+^CD27^−^CD28^−^	25	0.720	0.086	−1.17	102	350	157	178
17	PBMC – CM CD8^+^	25	0.280	0.086	0.90	103	351	228	196
18	FOXP3^+^ cells – Tumor centre – Proportion	27	0.730	0.060	−0.66	84	263	363	199
19	Gal‐9	29	0.720	0.062	−0.66	104	275	365	212
20	PBMC – CM CD8^+^CD27^+^	25	0.290	0.098	0.91	118	394	220	213
21	PBMC – CD4^+^CD57^+^	25	0.710	0.110	−1.08	130	438	174	218
22	PBMC – CM CD8^+^CD28^+^	25	0.290	0.109	0.89	119	433	232	226
23	miR‐9	29	0.340	0.056	10.00	332	252	4	230
24	PBMC – TEMRA CD8^+^CD27^+^	25	0.260	0.057	0.51	71	256	535	233
25	CD5^+^ cells – Tumor centre – Density	27	0.720	0.076	−0.62	106	322	408	236
26	PBMC – Total Treg cells	25	0.290	0.098	0.73	117	395	323	238
27	PBMC – Naive CD8^+^CD27^−^CD28^−^	25	0.710	0.097	−0.76	131	393	303	240
28	PBMC – CM CD8^+^CD27^+^CD28^+^	25	0.300	0.124	0.88	149	497	240	259
29	PBMC – CM CD4^+^CD27^+^	25	0.300	0.124	0.76	148	498	304	275
30	FOXP3^+^ cells – Whole tumor – Density	27	0.690	0.131	−0.96	199	525	209	283
31	PBMC – CD56^bright^CD16^−^ NK cells	25	0.300	0.123	0.69	150	493	345	285
32	PBMC – EM CD4^+^CD27^−^CD28^−^	25	0.680	0.153	−1.25	217	578	147	290
33	sCD25	29	0.710	0.069	−0.45	133	300	628	299
34	PBMC – TEMRA CD4^+^CD27^−^CD28^−^	25	0.680	0.157	−1.09	218	590	173	300
35	PBMC – TEMRA CD8^+^	25	0.710	0.098	−0.50	132	396	545	301
36	PBMC – CM CD4^+^CD27^+^CD28^+^	25	0.310	0.140	0.75	176	548	312	303
37	miR‐195	29	0.710	0.069	−0.43	134	301	660	307
38	CD5^+^ cells – Tumor centre – Proportion	27	0.730	0.060	−0.37	83	264	803	308
39	miR‐20a	29	0.720	0.056	−0.36	105	253	830	323
40	FOXP3^+^ cells – Invasive front – Density	27	0.670	0.176	−0.94	267	645	211	348
41	miR‐17	29	0.680	0.130	−0.49	220	521	560	380
42	PBMC – Naive CD8^+^CD28^+^	25	0.320	0.175	0.63	246	637	400	382
43	PBMC – Naive CD8^+^CD27^+^CD28^+^	25	0.320	0.175	0.61	244	638	421	387
44	PBMC – CM CD4^+^CD28^+^	25	0.330	0.194	0.81	289	701	269	387
45	PBMC – Naive CD8^+^CD27^+^	25	0.320	0.175	0.59	245	639	448	394
46	PBMC – TEMRA CD8^+^CD28^+^	25	0.320	0.175	0.54	247	640	495	407
47	PBMC – CM CD4^+^	25	0.340	0.215	0.80	330	752	277	422
48	CD4^+^ cells – Tumor centre – Density	27	0.680	0.145	−0.42	222	560	693	424
49	CD5^+^ cells – Invasive front – Proportion	27	0.690	0.118	−0.32	197	467	940	450
50	miR‐223	29	0.660	0.176	−0.51	312	646	539	452

The number of patients (*N*) for whom a specific marker could be assessed, the area under the curve (AUC), *P*‐value and log fold change (FC) are reported. Based on the statistical performance, the different markers were assigned a score for AUC, *P*‐value and log FC. In the final score, AUC weighted double compared to *P*‐value and log FC. For both age and G8, a 10‐biomarker panel (combination of the markers marked in light and dark grey) and a 5‐biomarker panel (marked in dark grey) were selected based on the statistical performance of a marker together with its biological relevance.

## Discussion

**Figure 6:**
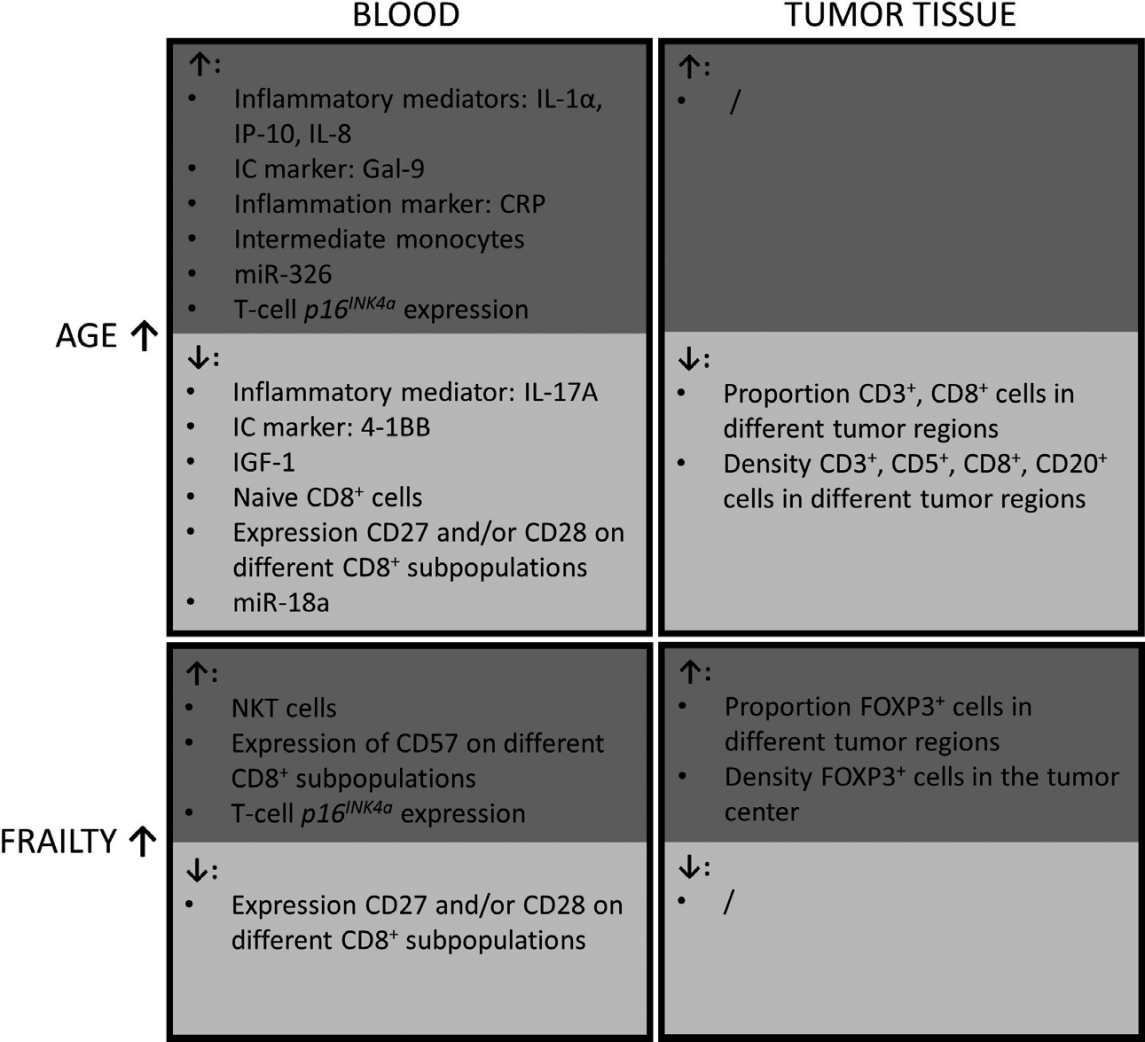
An overview of increases (↑) and decreases (↓) of the blood immune/senescence markers and the tumor immune infiltrate markers according to age and increasing frailty level. Notably, for some markers (not shown in this figure), the highest levels were present in the middle age category (e.g. blood levels of MCP‐1, sCD25, TIM‐3, miR‐19b, miR‐20a, miR‐195), while for other markers, lowest levels were present in the middle age category (PD‐L1 in blood, and sTILs % in tumor). IC, Immune checkpoint.

In this study, we have for the first time performed a comprehensive analysis of the immune/senescence profile in blood, together with a detailed characterisation of the tumor immune infiltrate in hormone‐sensitive BC in relation to the patient’s age and clinical frailty. Our main findings regarding the observed biomarker changes in function of ageing and frailty are basically summarised in Figure 6.

Overall, we observed huge variations in the degree of tumor immune infiltration in our cohort of patients with luminal breast cancer; yet, only a minority of tumors (8%) showed strong infiltration (≥ 40% sTILs). This indicates that luminal tumors are indeed less immunogenic than HER2‐positive or TNBC.^16^, ^18^ Interestingly, tumors of higher grade appeared to be more heavily infiltrated by immune cells, which has previously been reported by Smid *et al*.,^19^ and increasing tumor size was associated with shifts in TILs composition. Notably, nodal status (i.e. positive versus negative) was not correlated with any of the peripheral nor tumor immune biomarkers, suggesting that the impact of immune status/immunosenescence on lymph node metastasis may be limited for luminal BC.

When comparing the different age groups, our data revealed a decreased degree of lymphocytic infiltration in older patients and an altered immune infiltrate composition, with a markedly lower contribution of CD8^+^ cells. Within the oldest age group, we also investigated whether patient’s clinical fitness/frailty status, assessed by G8 score, had an impact on the tumor immune response. We observed a relatively more immunosuppressive FOXP3^+^ immune milieu, which could possibly suggest less effective immune response, in frail patients.

We also evaluated a comprehensive panel of immune/senescence markers in blood, in order (i) to investigate the impact of ageing on systemic immunity features (immunosenescence) in patients with luminal BC and (ii) to explore connections between tumor and blood immune profiles. Significant age‐related increases were noted for several plasma inflammatory cytokines (IL‐1α) and chemokines (IP‐10, IL‐8 and MCP‐1), which all play an eminent role in the migration, recruitment and activation of specific immune subsets.^20^, ^21^, ^22^, ^23^, ^24^ These observations are consistent with earlier studies reporting on a gradually increasing chronic, low‐grade inflammation in the old aged.^3^, ^21^, ^22^, ^25^, ^26^, ^27^ Despite their well‐established role in lymphocyte and macrophage migration, elevated plasma levels of the 3 chemokines in the older patients of our cohort were not associated with increased immune infiltration in the tumor; actually, the opposite was true: MCP‐1 showed a striking inverse correlation with sTILs %, more specifically with T‐cell subsets expressing CD3, CD5, CD8 and FOXP3. It would be interesting to study intratumoral levels of MCP‐1 and other chemokines in function of age, which might be more relevant than circulating chemokines. In the context of ageing and frailty, IL‐6 is one of the most described pro‐inflammatory cytokines, involved in acute as well as chronic inflammation.^23^, ^25^, ^26^, ^27^, ^28^ IL‐6 indeed showed a trend towards an age‐related increase in our cohort, although this association did not reach statistical significance. The clinical inflammation biomarker CRP, on the other hand, did significantly rise with ageing in our study. Both IL‐6 and CRP have consistently been reported to be strongly correlated with various age‐related diseases and are considered valuable predictors of physical and cognitive decline.^28^, ^29^ Furthermore, a significant age‐related decrease was seen for plasma IGF‐1, in accordance with the notion that IGF‐1 signalling diminishes with age and may play a role in biological ageing.^30^ Several immune checkpoint markers displayed altered plasma concentrations in function of ageing: Gal‐9, TIM‐3 and sCD25 levels were elevated at more advanced age, whereas age‐related decreases were seen for 4‐1BB and PD‐L1. To date, limited research has been carried out on soluble immune checkpoint markers plasma. TIM‐3 has been recognised as a T‐cell exhaustion marker, which is shed into the extracellular milieu.^31^ Gal‐9 is the most important ligand of TIM‐3^32^, ^33^ and sCD25 is the soluble form of IL‐2Ra, a cytokine involved in activation of Tregs.^34^ As T‐cell exhaustion and elevated Treg frequency/functional potential have both been associated with old age,^35^ plasma accumulation of TIM‐3, Gal‐9 and sCD25 at higher age is not unexpected. Although we could not demonstrate significantly increased frequencies of circulating Tregs nor exhausted/senescent (CD27^−^CD28^−^ or CD57^+^) T cells in the oldest patients of our cohort, we did find significant expansion of these specific cell subsets in blood of frailer patients within the oldest age category, concomitant with higher representation of FOXP3^+^ cells within their tumors. These findings possibly suggest that the previously reported increases in Tregs and exhausted/senescent T cells in older patients might rather be associated with progressive frailty than barely higher age.

Previous studies in the general (non‐cancer) population have shown that the cytotoxic CD8^+^ T‐cell population appears to be highly affected by age.^5^ Our current data further support these findings. Particularly, percentages of circulating naive CD8^+^ T cells significantly decreased with age, probably by immunological reserves depletion because of lifelong exposure to immune challenges. Moreover, smaller fractions of naive CD8^+^ T cells expressing CD27, CD28 or both were present in the higher age groups. These co‐receptors are important for T‐cell survival and activation by (tumor) antigens and are thus essential for effective T‐cell responses. Loss of CD27/CD28, which has been described as a hallmark of ageing,^5^, ^9^ could be partially responsible for decreased tumor infiltration by CD8^+^ cells observed in the older patients of our cohort. Apparently, circulating immune cells of older patients may be less capable to recognise tumor antigens, become activated and infiltrate into the tumor tissue. Interestingly, activated T cells, mainly CD8^+^ cells, also express the costimulatory receptor 4‐1BB. Its age‐related decrease in plasma, as observed in our study, could thus also be linked to impaired CD8^+^ cell activation.^36^

T‐cell expression of the cell cycle inhibitor *p16^INK4a^* has been proposed as a promising biomarker of ageing.^8^ Our data indeed demonstrated a significant increase of T‐cell *p16^INK4a^* expression with advancing age. However, because of its low or even unmeasurable expression in a considerable number of patients, accurate evaluation of *p16^INK4a^* was technically challenging, compromising this biomarker’s utility. Finally, six immune‐related miRs showed significantly different plasma expression levels across the age groups: miR‐18a decreased with age; miR‐155 and miR‐326 increased; and miR‐19b, miR‐20a and miR‐195 peaked in the middle group. miRs play critical roles in numerous and diverse developmental events and physiological pathways (including pathological and immune‐related processes) making it hard to determine their specific role at a given moment.^11^, ^37^ Especially for miR‐18a, miR‐19b and miR‐20a, the results should be considered with caution. These miRs all belong to the miR‐17‐92 cluster, which is highly involved in innate and adaptive immune responses and is important for T‐cell proliferation and survival,^12^, ^14^, ^37^ but, simultaneously, has also been implicated in cancer through its oncogenic effects.^38^, ^39^ Expression levels of miRs in this cluster were previously reported to be downregulated with increasing age.^40^ However, since we are considering a cancer population here, observed changes of these miRs in the age groups are difficult to interpret and opposed effects of ageing (downregulation) and cancer (upregulation) may explain the observed ambiguous dynamics of their plasma expression. The pro‐inflammatory miR‐155 showed a marked increase with age, which is in line with its reported active involvement in immune function and chronic inflammation.^14^, ^41^ Furthermore, plasma miR‐195 showed consistent positive correlations with the expression of immune markers within the tumor and was decreased in the oldest age group. Since this miR is known to enhance T‐cell activation in the tumor microenvironment by blocking the PD‐L1/programmed cell death protein 1 (PD‐1) immune checkpoint,^42^, ^43^ it may also play a role in the observed age‐related decrease in tumor immune infiltration.

Apart from age‐related changes, differences in (tumor) immunity between fit and frail patients could be equally important. Based on the G8 scores, the oldest patients were subdivided into a fitter group (G8 > 14) and a frailer group (G8 ≤ 14). In the frailer group, higher T‐cell *p16^INK4a^* expression was observed, in accordance with previous observations by Liu *et al*.,^8^ who even reported a stronger correlation of T‐cell *p16^INK4a^* expression with frailty than with chronical age. Furthermore, the G8‐score was also correlated with several PBMC subsets. In the frailer subgroup, a significantly increased percentage of a distinct CD3^+^CD16^+^ cell population (NK‐like T cells) was found, which had, however, remained relatively stable along the age groups. These cells have been described as cytotoxic effector cells involved in the anti‐cancer immune response.^44^ Yet, the link between accumulating CD3^+^CD16^+^ cells and clinical frailty remains unclear. Increased frailty was also associated with depletion of fully active CD8^+^ cells expressing CD27 and/or CD28 and, concurrently, accumulation of senescent CD8^+^CD57^+^ cells with decreased proliferative capacity. Surprisingly, in our cohort, no significant differences could be demonstrated between frailer and fitter patients with regard to the best documented frailty markers IL‐6, TNFα and CRP. Moreover, while Ipson *et al*.^45^ have identified circulating miR‐326 as a candidate frailty biomarker, our data only showed increased miR‐326 expression in the oldest age category, without any significant difference between G8 subgroups within the old aged. These discrepancies could possibly be explained by the relatively small numbers of fit versus frailer patients and a different frailty assessment used in our study.

A number of associations between blood biomarkers and the tumor infiltrate are interesting to be highlighted. Firstly, levels of several pro‐inflammatory mediators (e.g. MCP‐1, IL‐1α, intermediate monocytes) inversely correlated with different tumor immune infiltrate markers. Hence, low‐grade chronic inflammation associated with ageing may induce a less effective tumor immune response. Secondly, higher plasma levels of the inhibitory immune checkpoint marker TIM‐3^46^ correlated with lower sTILs % and lower infiltration of different immune cell subsets. Furthermore, higher frequencies of fully functional CD27^+^CD28^+/−^ TEMRA cells in blood positively correlated with all T‐ and B‐cell markers in the tumor, whereas expansion of the senescent CD27^−^CD28^−^ and CD57^+^ TEMRA cell blood populations was associated with increased infiltration of FOXP3^+^ cells in the tumor. Not surprisingly, blood Tregs % also inversely correlated with sTILs % and with CD8 density in the tumor. Finally, higher plasma levels of several miRs appeared to be associated with increased abundancy of T‐cell markers in the tumor immune infiltrate.

As a next step, we compiled panels consisting of the 5 or 10 highest significant and biologically most relevant age and frailty biomarkers. These panels proved able to quite accurately separate the oldest age group from the younger age groups, as well as the more ‘fit’ older patients from the ‘frailer’ older patients. Interestingly, uMap plotting with the biomarker panels for age revealed a distinct cluster of older patients exhibiting a clearly different ageing profile compared to the remaining older patients who mingled among the younger patients. Of note, virtually all ‘frail’ older patients clustered in the distinct group. This may suggest that the patients in this separate cluster are mainly ‘frail’ older patients or patients at risk of becoming frail, whereas the older patients mixed among the younger patients are the truly ‘fit’ older patients. Within the old group, uMap plotting with the frailty biomarker panels again showed a separation of the older patients in two clusters: a purely ‘fit’ group and a mixed group comprising all ‘frailer’ patients admixed with ‘fit’ individuals. We speculate that the latter show a biomarker profile that could point to beginning frailty development, which is not yet clinically apparent.

This study has some limitations. Primarily, it is a highly exploratory study without a hard primary endpoint. Further exhaustive research and validation of the findings are required. The study cohort was relatively small, and the old group was composed of relatively fit patients; only 10 patients had a G8 ≤ 14, and none of those had a G8 lower than 12. The difference in ‘frailty’ between the G8‐normal and G8‐decreased patients was thus rather moderate, but apparently still large enough to reveal some noticeable biomarker differences. Also, our study cohort did not allow conclusions on whether the changes in blood biomarkers are driven by ageing alone or are affected by the developing tumor as well. This could be further explored by comparing the blood immune/senescence profile of the patients in our cohort to age‐matched healthy control groups, which were unfortunately not available for this study. Furthermore, no long‐term outcome data are available to date, and also in the future, the limited sample size will not allow survival analysis. Finally, only a selection of potential immune/senescence biomarkers was evaluated; for instance, data on PD‐L1 expression and other immune checkpoint and activation markers on the tumor are not yet available. An extensive multiplex immunohistochemistry analysis, using the newly developed ‘Multiple Iterative Labeling by Antibody Neodeposition (MILAN)’ technique^47^ and including a multitude of additional immune‐related tumor markers is currently ongoing. These in depth analyses will allow us to characterise in more detail the phenotype and functional status of immune subsets of the tumor infiltrate, co‐expression of immune markers and the local interactions between different cell types in the tumor microenvironment. Additional multidimensional bioinformatics techniques will also be applied to the data, aiming at compiling a blood biomarker signature of immunosenescence that could accurately reflect the tumor immune response in older BC patients.

Nevertheless, our study also has major strengths. Most importantly, blood and tumor tissue has been prospectively collected from a relatively homogenous group of patients with a similar luminal breast tumor (hormone‐sensitive, HER2‐negative). In this tumor type, the tumor immune infiltrate has been poorly studied despite it is the most frequent BC subtype. Our investigations thus contributed to further disclosure of the largely unexplored immunological landscape of luminal breast tumors. To the best of our knowledge, this is also the first BC study where the tumor immune infiltrate has been characterised in concert with the peripheral blood immune profile and in function of ageing and clinical frailty. This allowed us to establish interesting correlations between immunosenescence markers in the blood, tumor immune biomarkers and ageing/clinical frailty.

In conclusion, this extensive immune biomarker study revealed several interesting age‐related modifications in the blood immunological portrait and local tumor immune response of patients with luminal BC, as well as differences between fit and frailer old patients. The observed changes can be linked to various processes occurring during the ageing process, such as inflammation, cellular senescence and immunosenescence.^2^ Our data support age‐dependent remodelling of both systemic immunity features and anti‐tumor immune responses. Given this knowledge that interactions between tumor cells, immune cells and inflammatory mediators differ with age, older patients may react differently to BC treatments like chemotherapy or (in the future), immunotherapy approaches. Ageing/immunosenescence should therefore be taken into account when outlining the optimal treatment strategy for each individual patient.

## Methods

### Patient selection

Patients were eligible for this prospective biomarker study, named IMAGE (IMmunity‐AGE), if they met the following inclusion criteria and upon written informed consent: early BC diagnosis, planned upfront surgery; grade II or III invasive carcinoma on core needle biopsy (CNB); ER‐positive, HER2‐negative; and clinical tumor size 1.5 cm or bigger on imaging with sufficient formalin‐fixed and paraffin‐embedded (FFPE) tumor tissue. We aimed to recruit patients within 3 distinct age categories: 35–45 years (premenopausal young population), 55‐65 years (postmenopausal but not ‘old’) and patients ≥ 70 years (postmenopausal older population). To evaluate blood immune parameters, an extra blood sample was drawn and processed at inclusion (before surgery). In the ≥ 70 years group, the G8 screening tool was used at inclusion (before surgery) as a surrogate for clinical frailty.

### G8 assessment

The G8 questionnaire is an easy, widely used geriatric screening tool^48^ that can be considered as surrogate for frailty.^49^ The G8 includes eight items: seven items selected from the mini nutritional assessment (MNA) questionnaire (i.e. nutritional status, weight loss, body mass index, motor skills, psychological status, number of medications and self‐perception of health) and one item indicating the patient’s age (<80; 80–85; >85). The G8 score ranges from 17 (not at all impaired) to 0 (heavily impaired). Patients with a score 14 or lower are considered vulnerable or frail, and patients with a score higher than 14 are considered fit.^48^, ^50^

### Blood collection and biomarker analysis

A detailed description of the materials and methods listed below can be found in Supplementary methods.

Blood was collected in two 10‐mL EDTA tubes (BD Vacutainer^®^; Becton, Dickinson and company, NJ), 4 mL of the total volume was used for EDTA plasma collection, 6 mL for T‐cell isolation, and the remaining blood was used for PBMC isolation, as described in detail in Supplementary methods. Briefly, PBMCs were isolated *via* density gradient centrifugation with Histopaque‐1077^®^ (Sigma‐Aldrich; Saint Louis), using SepMate‐50 tubes (StemCell Technologies, Vancouver) for easy separation of fractions. For T‐cell isolation, RosetteSep Human T‐Cell Enrichment Cocktail (StemCell Technologies, Vancouver) was additionally added.

A broad panel of plasma cytokines [IL‐1α; IL‐1β; IL‐6; IL‐10; IL‐12p70; IL‐17A; IL‐17F; IL‐27; interferon gamma (INFγ); TNFα and free active transforming growth factor‐beta 1 (TGF‐β1)], plasma chemokines (i.e. IP‐10; IL‐8; MCP‐1), immune checkpoint proteins [sCD25; 4‐1BB; CD86; cytotoxic T‐lymphocyte‐associated antigen 4 (CTLA‐4); PD‐L1; PD‐1; TIM‐3; lymphocyte‐activation gene 3 (LAG‐3); Gal‐9; sCD27; programmed cell death‐ligand 2 (PD‐L2)] and C‐reactive protein (CRP) were measured by cytometric bead array assays (AimPlex Human Inflammation 11‐plex; ImTec Diagnostics, Antwerp and YSL AimPlex; BioLegend, San Diego). Insulin‐like growth factor (IGF‐1) levels in plasma were measured by ELISA (Human IGF‐I Quantikine ELISA kit; R&D Systems, Minneapolis). T‐cell *p16^INK4a^* expression was measured *via* a probe‐based RT‐qPCR. Plasma levels of 20 immune‐related miRs (i.e. let‐7e, let‐7i, miR‐9, miR‐17, miR‐18a, miR‐19a, miR‐19b, miR‐20a, miR‐21, miR‐92a, miR‐125b, miR‐126, miR‐146a, miR‐150, miR‐155, miR‐181a, miR‐195, miR‐223, miR‐326 and miR‐424) were measured *via* SYBR Green RT‐qPCR. By using fluorescent antibody panels (Supplementary figure 1), PBMC immune subset profiles were analysed by 8‐colour flow cytometry, using a 3‐laser FACSVerse (BD Biosciences; Becton, Dickinson and company, NJ). All biomarker assay procedures are described in detail in Supplementary methods.

### Tumor immune marker analysis

For the final analysis, we used tumor grade and pathological TMN staging measured on the resection specimen, which explained some discrepancies with grading on CNB and tumor size on imaging. All stainings were performed on surgical resection specimen. FFPE tumor tissue sections were cut at a thickness of 5 µm. sTILs were microscopically assessed on representative haematoxylin and eosin (H&E) stained tumor sections, according to published guidelines.^51^ Further tumor immune infiltrate characterisation was performed by evaluating 7 immune cell markers (CD3, CD4, CD5, CD8, CD20, CD68 and FOXP3) via immunohistochemistry on whole section. CD68 staining grade was determined using a reported method.^52^ The remaining immune cell markers (CD3, CD4, CD5, CD8, CD20 and FOXP3) were scored using a scoring protocol in the QuPath software to compare density and proportion of immune cells in different tumor regions.^53^

### Statistics

This is a prospective exploratory biomarker study; thus, no upfront sample size calculation was performed. Our aim was to include at least 15 patients for the young and middle age categories and 30 for the ≥ 70 years category, allowing to subdivide older patients in fitter group (G8 > 14) and frailer group (G8 ≤ 14). Because of the exploratory character of the study, statistical tests were performed without correction for multiple testing. The Mann–Whitney *U*‐test was used for associations between continuous and binary variables with more than two groups. The Kruskal–Wallis test was used for associations between continuous and categorical variables. Interactions between tumor region and age or G8 groups on the proportion and density of positively stained cells were assessed using linear mixed models, accounting for clustering of regions within patients. Spearman correlations were used for associations between different biomarkers. The analyses were performed using SAS software (SAS, Cary). Additionally, R software (R project, Vienna) was used to calculate biomarker performance and generate the dimensionally reduced data. Biomarkers were ranked using a combination of quantitative [fold change (FC) and *P*‐value (ANOVA)] and qualitative (area under the ROC curve (AUC)) parameters. The final score was calculated using a weighted average of the individual scores. The weights were set to 2:1:1 for AUC:FC:*P*‐value; that is, AUC was weighted double compared to *P*‐value and FC. Panels of 10 and 5 biomarkers were compiled based on their individual statistical performance and biological relevance for both age groups and G8 groups. These panels were dimensionally reduced using three different methods: uniform manifold approximation and projection (uMAP), principal component analysis (PCA) and T‐distributed stochastic neighbour embedding (t‐SNE). Biomarkers were normalised using *Z*‐scores before dimensionality reduction. Patient clustering was performed visually on the dimensionally reduced plots. Visually defined clusters were evaluated for enrichment with specific patient groups using hypergeometric tests. All reported *P*‐values are two‐sided, and significance threshold was set below 5% for all tests.

## Conflict of interest

The authors declare no conflict of interest

## Author contributions

**Lieze Berben:** Data curation; Formal analysis; Funding acquisition; Investigation; Methodology; Validation; Visualization; Writing‐original draft; Writing‐review & editing. **Giuseppe Floris:** Conceptualization; Data curation; Formal analysis; Investigation; Methodology; Supervision; Writing‐original draft; Writing‐review & editing. **Cindy Kenis:** Data curation; Writing‐review & editing. **Bruna Dalmasso:** Data curation; Writing‐review & editing. **Ann Smeets:** Data curation; Writing‐review & editing. **Hanne Vos:** Data curation; Writing‐review & editing. **Patrick Neven:** Data curation; Writing‐review & editing. **Asier Antoranz Martinez:** Formal analysis; Software; Visualization; Writing‐review & editing. **Annouschka Laenen:** Formal analysis; Software; Visualization; Writing‐review & editing. **Hans Wildiers:** Conceptualization; Data curation; Funding acquisition; Investigation; Methodology; Project administration; Supervision; Writing‐original draft; Writing‐review & editing. **Sigrid Hatse:** Conceptualization; Data curation; Formal analysis; Investigation; Methodology; Supervision; Writing‐original draft; Writing‐review & editing.
