# Supplementary material for: Age‐related remodelling of the blood immunological portrait and the local tumor immune response in patients with luminal breast cancer
Source: Clin Transl Immunology. 2020 Oct 3;9(10):e1184. doi: 10.1002/cti2.1184 (PMC7532981; doi:10.1002/cti2.1184)
Supplement: Supplementary file 1 [file CTI2-9-e1184-s001.docx]

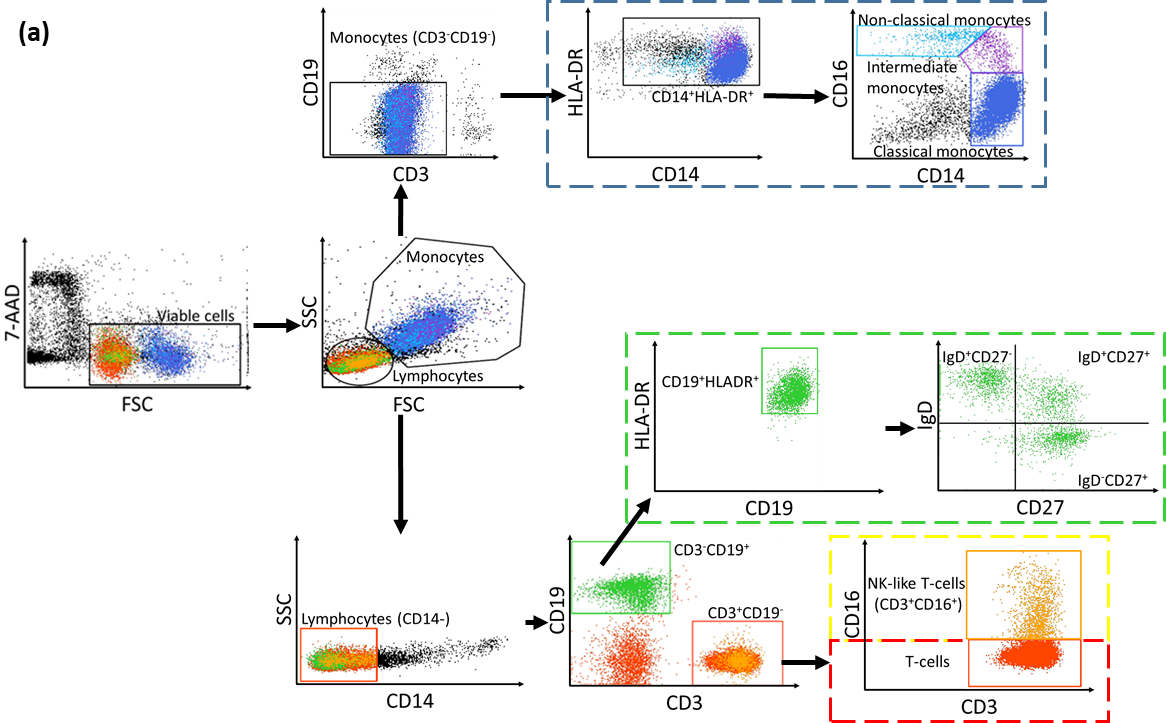


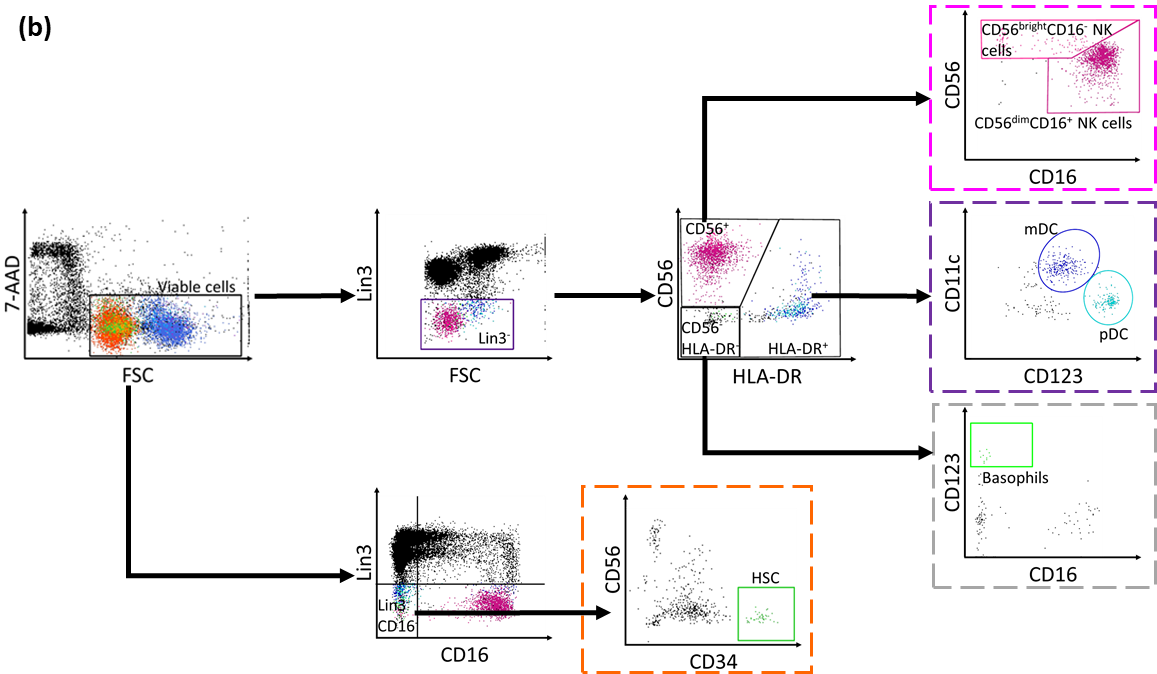


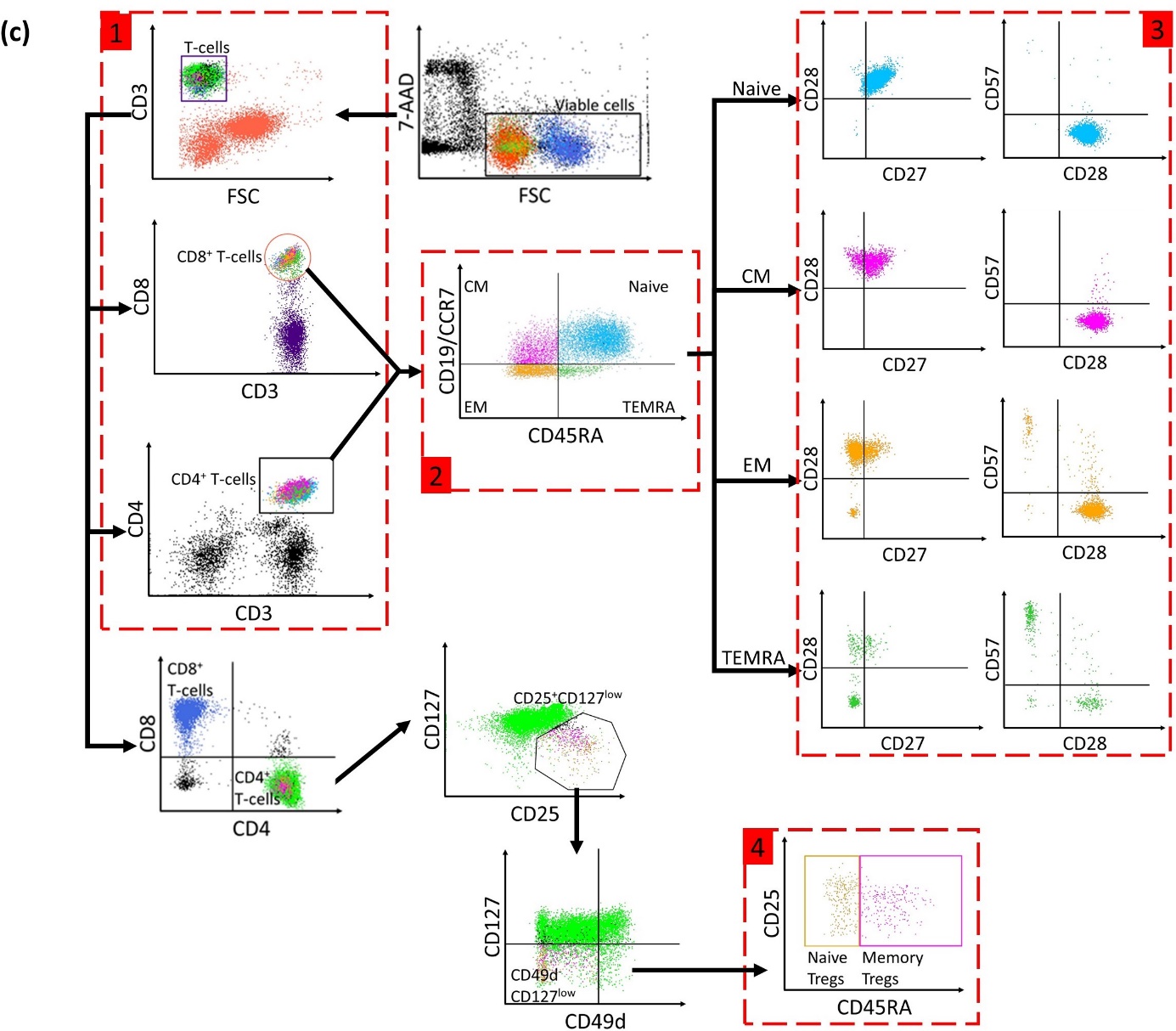


Supplementary figure 1: Schematic overview of the flow cytometry gating strategy applied for PBMC subset profiling with 8-color staining panels. Fluorescence minus one (FMO) controls were used to discriminate between positive and negative signals and to aid gating of the different cell populations. All staining panels included 7-AAD for dead cell exclusion (first gate). The reported PBMC subset populations are depicted in different colors. **(a)** Major PBMC subsets, including monocytes (blue), B-cells (green), NKT-like cells (CD3^+^CD16^+^) (yellow) and T-cells (red) were determined using a panel with the following antibody-fluorochrome combination: CD3-FITC, CD19-APC.H7, HLA-DR-BV510, CD14-APC, CD16-PE.Cy7, CD27-PE and IgD-BV421. **(b)** A staining panel with Lin3-FITC (dump channel for cells expressing CD3, CD14, CD19, CD20), CD56-BV421, HLA-DR-APC.H7, CD16-APC, CD11c-BV510, CD123-PE and CD34-PE.Cy7 identified NK cell populations (pink), basophils (grey), dendritic cells (purple) and HSCs (orange). **(c)** For deeper characterization of the T-cell subsets (red), the following panels were used: CD3-FITC, CD4-PE.Cy7 or CD8-PE.Cy7, CD45RA-BV510, CCR7-BV421, CD28-APC, CD27-APC.H7,CD57-PE, allowing delineation of CD4+ and CD8+ T-cells (1, red), naive and memory T-cell subsets (2, red) and expression of CD27, CD28 and CD57 within the different T-cell subpopulations (3, red); and CD3-FITC, CD8-APC.H7, CD4-BV510, CD127-APC, CD25-BV421, CD49d-PE,CD45RA-PE.Cy7 for identification of Tregs (4, red).
