# Supplementary material for: Age‐related remodelling of the blood immunological portrait and the local tumor immune response in patients with luminal breast cancer
Source: Clin Transl Immunology. 2020 Oct 3;9(10):e1184. doi: 10.1002/cti2.1184 (PMC7532981; doi:10.1002/cti2.1184)
Supplement: Supplementary file 2 [file CTI2-9-e1184-s002.docx]

*Supplementary table 1: Blood immune/senescence markers (plasma protein biomarkers, PBMC subset profiling, T-cell p16^INK4a^ expression and plasma circulating miRs) in the different age categories: young (35-45 years), middle (55-65 years) and old (≥70 years). The number of measurements (N), median, inter quartile range (IQR) and the P-values are reported. The P-values were calculated via the Kruskal-Wallis test, significance threshold was set below 5% (marked in grey).*

|  | **Young (35-45 years)** | | | **Middle (55-65 years)** | | | **Old (≥70 years)** | | | *P*-value |
| --- | --- | --- | --- | --- | --- | --- | --- | --- | --- | --- |
|  | N | Median | IQR | N | Median | IQR | N | Median | IQR |  |
| ***Plasma protein biomarkers*** | | | | | | | | | | |
| IL-1α (pg/mL) | 15 | 10.6 | 8.1; 12.0 | 19 | 11.7 | 9.1; 15.6 | 31 | 16.4 | 14.5; 20.3 | < 0.001 |
| IL-17A (pg/mL) | 15 | 1.2 | 0.6; 2.7 | 19 | 0.7 | 0.4; 1.4 | 31 | 0.5 | 0.2; 0.9 | 0.012 |
| IL-1β (pg/mL) | 15 | 13.1 | 6.8; 20.6 | 19 | 9.1 | 4.3; 14.4 | 31 | 11.1 | 3.4; 22.4 | 0.805 |
| IL-6 (pg/mL) | 15 | 6.6 | 4.9; 7.7 | 19 | 9.5 | 6.0; 15.6 | 31 | 10.0 | 5.4; 15.3 | 0.062 |
| IL-10 (pg/mL) | 15 | 8.8 | 4.5; 13.6 | 19 | 5.6 | 3.4; 11.1 | 31 | 7.3 | 2.8; 13.1 | 0.558 |
| IL-12p70 (pg/mL) | 15 | 6.3 | 3.8; 9.3 | 19 | 5.2 | 3.3; 12.6 | 31 | 8.7 | 3.3; 12.8 | 0.870 |
| IL-17F (pg/mL) | 15 | 0.0 | 0.0; 0.0 | 19 | 0.0 | 0.0; 0.0 | 31 | 0.0 | 0.0; 0.0 | 0.422 |
| IL-27 (pg/mL) | 15 | 6.5 | 5.1; 8.1 | 19 | 6.2 | 4.6; 9.4 | 31 | 7.9 | 4.4; 9.2 | 0.976 |
| IFNγ (pg/mL) | 15 | 9.2 | 6.4; 13.8 | 19 | 7.7 | 4.8; 13.2 | 31 | 9.5 | 4.1; 15.6 | 0.899 |
| TNFα (pg/mL) | 15 | 3.5 | 1.3; 4.7 | 19 | 2.2 | 1.6; 4.4 | 31 | 3.8 | 1.3; 6.9 | 0.864 |
| IP-10 (pg/mL) | 15 | 51.2 | 42.3; 56.9 | 19 | 58.8 | 47.5; 74.7 | 31 | 70.8 | 58.7; 91.8 | < 0.001 |
| IL-8 (pg/mL) | 15 | 16.3 | 14.0; 20.2 | 19 | 22.0 | 16.8; 29.5 | 31 | 22.0 | 19.2; 27.3 | 0.011 |
| MCP-1 (pg/mL) | 15 | 96.5 | 85.4; 107.6 | 19 | 140.7 | 116.2; 149.2 | 31 | 130.9 | 102.4; 163.6 | 0.001 |
| Gal-9 (ng/mL) | 15 | 28.7 | 23.4; 38.0 | 19 | 54.1 | 44.2; 66.9 | 31 | 67.0 | 47.0; 86.4 | < 0.001 |
| sCD25 (pg/mL) | 15 | 503.7 | 388.8; 699.2 | 19 | 921.0 | 571.9; 1217.5 | 31 | 737.8 | 580.0; 1170.1 | 0.008 |
| TIM-3 (ng/mL) | 15 | 4.3 | 3.9; 5.8 | 19 | 8.6 | 5.5; 10.4 | 31 | 7.5 | 4.6; 11.2 | 0.017 |
| 4-1BB (pg/mL) | 15 | 1.0 | 0.0; 22.5 | 19 | 0.0 | 0.0; 1.9 | 31 | 0.0 | 0.0; 0.0 | 0.025 |
| PD-L1 (pg/mL) | 15 | 1.9 | 1.2; 4.0 | 19 | 0.9 | 0.7; 1.4 | 31 | 1.2 | 0.9; 1.7 | 0.008 |
| sCD27 (ng/mL) | 15 | 37.8 | 24.1; 50.9 | 19 | 38.3 | 25.7; 44.4 | 31 | 40.2 | 29.6; 52.0 | 0.673 |
| CD86 (pg/mL) | 15 | 119.2 | 88.1; 139.9 | 19 | 142.0 | 102.4; 169.9 | 31 | 119.2 | 83.6; 167.0 | 0.269 |
| CTLA-4 (pg/mL) | 15 | 0.0 | 0.0; 1.2 | 19 | 0.0 | 0.0; 0.0 | 31 | 0.0 | 0.0; 0.0 | 0.066 |
| Free active TGF-β1 (pg/mL) | 15 | 0.0 | 0.0; 0.0 | 19 | 0.0 | 0.0; 0.0 | 31 | 0.0 | 0.0; 0.0 | 0.383 |
| LAG-3 (pg/mL) | 15 | 643.7 | 277.0; 1065.4 | 19 | 463.3 | 289.9; 630.1 | 31 | 463.4 | 326.3; 735.2 | 0.602 |
| PD-1 (pg/mL) | 15 | 0.5 | 0.3; 1.0 | 19 | 0.3 | 0.1; 0.7 | 31 | 0.3 | 0.2; 0.5 | 0.320 |
| PD-L2 (ng/mL) | 15 | 9.7 | 7.7; 13.9 | 19 | 8.9 | 6.7; 12.8 | 31 | 8.3 | 6.9; 10.2 | 0.286 |
| CRP (ng/mL) | 15 | 450.0 | 252.9; 849.8 | 19 | 834.4 | 526.5; 1672.0 | 31 | 1030.8 | 420.1; 1431.5 | 0.030 |
| IGF-1 (pg/mL) | 15 | 112.4 | 93.2; 153.4 | 19 | 89.3 | 77.2; 116.5 | 31 | 78.0 | 66.3; 102.4 | 0.002 |
| ***PBMC subset profiling*** | | | | | | | | | | |
| CD3^+^ cells (%) | 15 | 50.1 | 46.1; 53.6 | 16 | 47.1 | 42.3; 57.2 | 26 | 49.3 | 43.6; 58.1 | 0.809 |
| CD4^+^ cells (%) | 15 | 29.0 | 20.9; 31.9 | 16 | 25.6 | 15.9; 37.2 | 26 | 25.4 | 20.1; 37.2 | 0.930 |
| CD8^+^ cells (%) | 15 | 16.9 | 12.0; 23.5 | 16 | 16.1 | 11.4; 22.7 | 26 | 16.2 | 10.3; 23.0 | 0.774 |
| CD4/CD8 ratio (%) | 15 | 1.8 | 1.1; 2.6 | 16 | 1.9 | 0.8; 2.5 | 26 | 1.6 | 0.7; 3.6 | 0.998 |
| Total Treg cells (%) | 15 | 0.9 | 0.5; 1.3 | 16 | 1.2 | 0.7; 1.7 | 26 | 1.0 | 0.5; 1.9 | 0.593 |
| Naive Treg cells (%) | 15 | 62.3 | 52.4; 77.1 | 16 | 71.5 | 60.7; 77.1 | 26 | 65.6 | 58.1; 76.2 | 0.518 |
| Memory Treg cells (%) | 15 | 37.1 | 21.8; 46.3 | 16 | 28.1 | 21.7; 38.9 | 26 | 32.0 | 20.3; 38.0 | 0.474 |
| CD3^+^CD16^+^ cells (%) | 15 | 3.0 | 1.4; 7.2 | 16 | 2.3 | 1.4; 3.6 | 26 | 3.4 | 1.6; 5.7 | 0.378 |
| Total NK cells (%) | 15 | 6.8 | 6.3; 11.6 | 16 | 5.7 | 3.6; 10.2 | 26 | 9.1 | 5.2; 13.3 | 0.269 |
| CD56^bright^CD16^-^ NK cells (%) | 15 | 9.3 | 5.1; 11.9 | 16 | 8.2 | 2.6; 10.5 | 26 | 4.8 | 3.1; 6.1 | 0.056 |
| CD56^dim^CD16^+^ NK cells (%) | 15 | 88.3 | 79.6; 93.1 | 16 | 83.9 | 77.2; 92.4 | 26 | 91.2 | 87.1; 94.7 | 0.123 |
| B-cells (%) | 15 | 11.8 | 5.0; 13.7 | 16 | 12.1 | 8.0; 21.4 | 26 | 9.2 | 6.6; 12.6 | 0.337 |
| Naive B-cells (%) | 15 | 40.1 | 24.0; 64.0 | 16 | 51.2 | 39.1; 65.4 | 26 | 43.4 | 31.9; 58.9 | 0.359 |
| Non-switched memory B-cells (%) | 15 | 27.1 | 19.2; 31.8 | 16 | 20.6 | 13.2; 26.5 | 26 | 19.7 | 8.6; 28.2 | 0.118 |
| Class-switched memory B-cells (%) | 15 | 28.2 | 15.4; 40.5 | 16 | 22.4 | 18.9; 35.7 | 26 | 27.3 | 16.6; 37.5 | 0.879 |
| Monocytes (%) | 15 | 16.6 | 8.5; 19.7 | 16 | 10.3 | 7.7; 18.0 | 26 | 14.5 | 8.1; 18.0 | 0.674 |
| Classical monocytes (%) | 15 | 85.9 | 82.5; 92.4 | 16 | 82.4 | 75.4; 87.1 | 26 | 81.3 | 75.8; 87.9 | 0.137 |
| Intermediate monocytes (%) | 15 | 5.1 | 3.8; 8.4 | 16 | 8.0 | 5.4; 13.2 | 26 | 9.2 | 6.1; 11.3 | 0.019 |
| Non-classical monocytes (%) | 15 | 6.8 | 3.7; 9.5 | 16 | 8.5 | 5.6; 10.9 | 26 | 8.8 | 4.9; 13.6 | 0.535 |
| Total pDC (%) | 15 | 0.3 | 0.1; 0.5 | 16 | 0.3 | 0.1; 0.5 | 26 | 0.1 | 0.1; 0.4 | 0.212 |
| Total mDC (%) | 15 | 0.7 | 0.6; 1.6 | 16 | 1.2 | 0.9; 2.0 | 26 | 1.0 | 0.6; 1.6 | 0.290 |
| Total HSC (%) | 15 | 0.1 | 0.1; 0.1 | 16 | 0.1 | 0.0; 0.1 | 26 | 0.1 | 0.0; 0.1 | 0.325 |
| CD4^+^CD27^+^ (%) | 15 | 71.5 | 62.0; 83.0 | 16 | 78.7 | 51.8; 84.0 | 26 | 68.2 | 55.5; 77.8 | 0.500 |
| CD4^+^CD28^+^ (%) | 15 | 97.1 | 93.1; 99.1 | 16 | 95.9 | 81.9; 99.3 | 26 | 96.3 | 73.9; 99.2 | 0.876 |
| CD4^+^CD27^+^CD28^+^ (%) | 15 | 71.5 | 61.4; 82.8 | 16 | 76.9 | 51.6; 83.8 | 26 | 64.8 | 52.9; 77.2 | 0.374 |
| CD4^+^CD27^-^CD28^-^ (%) | 15 | 2.7 | 0.6; 6.6 | 16 | 2.5 | 0.3; 17.9 | 26 | 3.2 | 0.5; 23.6 | 0.862 |
| CD4^+^CD57^+^ (%) | 15 | 6.2 | 1.8; 13.2 | 16 | 6.3 | 2.2; 18.1 | 26 | 7.3 | 2.1; 23.0 | 0.741 |
| CD4^+^ Tregs (%) | 15 | 3.0 | 2.2; 5.0 | 16 | 3.9 | 3.0; 5.8 | 26 | 3.8 | 2.9; 6.4 | 0.297 |
| Naive CD4^+^ (%) | 15 | 39.5 | 27.2; 51.3 | 16 | 29.4 | 16.8; 47.6 | 26 | 30.7 | 20.9; 45.2 | 0.476 |
| Naive CD4^+^CD27^+^ (%) | 15 | 38.3 | 25.8; 51.0 | 16 | 28.9 | 15.4; 45.1 | 26 | 28.4 | 19.9; 44.0 | 0.416 |
| Naive CD4^+^CD28^+^ (%) | 15 | 39.0 | 26.8; 51.0 | 16 | 29.3 | 15.6; 43.9 | 26 | 30.1 | 19.6; 44.9 | 0.397 |
| Naive CD4^+^CD27^+^CD28^+^ (%) | 15 | 38.3 | 25.6; 50.7 | 16 | 28.9 | 14.9; 42.4 | 26 | 28.2 | 16.4; 44.0 | 0.359 |
| Naive CD4^+^CD27^-^CD28^-^ (%) | 15 | 0.1 | 0.0; 0.3 | 16 | 0.2 | 0.1; 0.5 | 26 | 0.2 | 0.1; 0.5 | 0.192 |
| Naive CD4^+^CD57^+^ (%) | 15 | 0.5 | 0.2; 1.3 | 16 | 0.6 | 0.1; 3.7 | 26 | 1.0 | 0.3; 2.7 | 0.534 |
| CM CD4^+^ (%) | 15 | 13.4 | 10.8; 18.8 | 16 | 17.9 | 14.5; 32.8 | 26 | 16.2 | 11.2; 26.3 | 0.367 |
| CM CD4^+^CD27^+^ (%) | 15 | 12.9 | 9.4; 17.2 | 16 | 15.5 | 12.6; 27.1 | 26 | 11.8 | 9.0; 23.8 | 0.352 |
| CM CD4^+^CD28^+^ (%) | 15 | 13.4 | 10.8; 18.8 | 16 | 17.9 | 14.2; 32.8 | 26 | 16.2 | 10.4; 26.3 | 0.367 |
| CM CD4^+^CD27^+^CD28^+^ (%) | 15 | 12.8 | 9.4; 17.2 | 16 | 15.4 | 12.6; 27.0 | 26 | 11.8 | 9.0; 23.8 | 0.355 |
| CM CD4^+^CD27^-^CD28^-^ (%) | 15 | 0.0 | 0.0; 0.0 | 16 | 0.0 | 0.0; 0.1 | 26 | 0.0 | 0.0; 0.1 | 0.685 |
| CM CD4^+^CD57^+^ (%) | 15 | 0.2 | 0.1; 0.4 | 16 | 0.2 | 0.0; 0.4 | 26 | 0.2 | 0.1; 0.5 | 0.834 |
| EM CD4^+^ (%) | 15 | 32.0 | 22.4; 45.4 | 16 | 23.2 | 20.7; 39.7 | 26 | 29.9 | 23.6; 37.3 | 0.517 |
| EM CD4^+^CD27^+^ (%) | 15 | 14.3 | 12.4; 18.3 | 16 | 10.7 | 7.8; 14.4 | 26 | 12.2 | 7.1; 18.6 | 0.294 |
| EM CD4^+^CD28^+^ (%) | 15 | 27.4 | 21.8; 38.4 | 16 | 21.2 | 15.1; 32.9 | 26 | 25.2 | 18.4; 32.3 | 0.307 |
| EM CD4^+^CD27^+^CD28^+^ (%) | 15 | 13.9 | 12.4; 18.2 | 16 | 10.7 | 7.8; 14.2 | 26 | 11.9 | 7.1; 18.6 | 0.285 |
| EM CD4^+^CD27^-^CD28^-^ (%) | 15 | 0.5 | 0.1; 1.6 | 16 | 0.2 | 0.1; 3.3 | 26 | 0.5 | 0.2; 6.3 | 0.706 |
| EM CD4^+^CD57^+^ (%) | 15 | 1.2 | 0.7; 7.1 | 16 | 1.3 | 0.3; 3.7 | 26 | 1.7 | 0.7; 6.5 | 0.541 |
| TEMRA CD4^+^ (%) | 15 | 7.7 | 6.5; 19.0 | 16 | 8.5 | 3.7; 19.8 | 26 | 10.6 | 5.7; 21.6 | 0.490 |
| TEMRA CD4^+^CD27^+^ (%) | 15 | 4.4 | 2.2; 7.0 | 16 | 2.3 | 1.2; 6.8 | 26 | 3.8 | 1.6; 5.8 | 0.464 |
| TEMRA CD4^+^CD28^+^ (%) | 15 | 6.7 | 4.4; 10.6 | 16 | 3.9 | 2.1; 8.3 | 26 | 5.0 | 2.4; 9.4 | 0.399 |
| TEMRA CD4^+^CD27^+^CD28^+^ (%) | 15 | 4.3 | 2.2; 6.9 | 16 | 2.2 | 1.0; 6.7 | 26 | 3.4 | 1.2; 5.8 | 0.416 |
| TEMRA CD4^+^CD27^-^CD28^-^ (%) | 15 | 1.6 | 0.4; 3.2 | 16 | 0.5 | 0.1; 7.7 | 26 | 1.1 | 0.2; 9.6 | 0.738 |
| TEMRA CD4^+^CD57^+^ (%) | 15 | 2.1 | 0.9; 3.3 | 16 | 0.6 | 0.2; 7.1 | 26 | 0.9 | 0.5; 9.8 | 0.746 |
| CD8^+^CD27^+^ (%) | 15 | 50.7 | 32.3; 59.0 | 16 | 52.2 | 36.0; 61.9 | 26 | 32.2 | 18.9; 51.5 | 0.036 |
| CD8^+^CD28^+^ (%) | 15 | 66.1 | 50.5; 75.1 | 16 | 70.3 | 55.9; 76.6 | 26 | 61.6 | 31.7; 72.8 | 0.329 |
| CD8^+^CD27^+^CD28^+^ (%) | 15 | 44.5 | 25.0; 56.3 | 16 | 45.1 | 29.2; 55.2 | 26 | 27.6 | 17.3; 46.4 | 0.066 |
| CD8^+^CD27^-^CD28^-^ (%) | 15 | 32.5 | 17.2; 44.9 | 16 | 21.4 | 16.6; 36.5 | 26 | 37.1 | 20.3; 61.2 | 0.191 |
| CD8^+^CD57^+^ (%) | 15 | 28.6 | 23.6; 44.1 | 16 | 31.3 | 14.5; 44.5 | 26 | 35.1 | 22.8; 47.4 | 0.635 |
| Naive CD8^+^ (%) | 15 | 25.4 | 8.7; 31.9 | 16 | 16.5 | 8.7; 28.3 | 26 | 9.6 | 4.5; 16.8 | 0.035 |
| Naive CD8^+^CD27^+^ (%) | 15 | 22.9 | 6.7; 28.6 | 16 | 14.6 | 6.4; 26.6 | 26 | 6.7 | 3.1; 13.6 | 0.018 |
| Naive CD8^+^CD28^+^ (%) | 15 | 23.7 | 7.3; 30.3 | 16 | 16.1 | 6.5; 27.2 | 26 | 7.9 | 3.0; 16.1 | 0.031 |
| Naive CD8^+^CD27^+^CD28^+^ (%) | 15 | 22.5 | 6.6; 27.6 | 16 | 13.9 | 5.6; 26.2 | 26 | 6.5 | 3.0; 13.4 | 0.021 |
| Naive CD8^+^CD27^-^CD28^-^ (%) | 15 | 0.7 | 0.3; 1.3 | 16 | 1.0 | 0.2; 1.5 | 26 | 0.5 | 0.2; 1.5 | 0.728 |
| Naive CD8^+^CD57^+^ (%) | 15 | 1.2 | 0.3; 2.4 | 16 | 1.6 | 0.4; 4.0 | 26 | 1.1 | 0.3; 2.7 | 0.682 |
| CM CD8^+^ (%) | 15 | 5.2 | 3.1; 6.6 | 16 | 10.7 | 4.4; 18.8 | 26 | 6.9 | 3.4; 12.9 | 0.113 |
| CM CD8^+^CD27^+^ (%) | 15 | 4.2 | 2.3; 5.3 | 16 | 9.0 | 3.4; 12.8 | 26 | 4.3 | 2.4; 8.4 | 0.154 |
| CM CD8^+^CD28^+^ (%) | 15 | 4.6 | 2.9; 6.0 | 16 | 10.1 | 3.7; 18.7 | 26 | 6.3 | 3.2; 11.3 | 0.131 |
| CM CD8^+^CD27^+^CD28^+^ (%) | 15 | 3.9 | 2.0; 4.9 | 16 | 8.8 | 3.1; 12.6 | 26 | 4.0 | 2.4; 8.2 | 0.164 |
| CM CD8^+^CD27^-^CD28^-^ (%) | 15 | 0.2 | 0.1; 0.4 | 16 | 0.2 | 0.0; 0.5 | 26 | 0.2 | 0.1; 0.5 | 0.895 |
| CM CD8^+^CD57^+^ (%) | 15 | 0.4 | 0.1; 0.7 | 16 | 0.9 | 0.1; 1.4 | 26 | 0.5 | 0.2; 1.0 | 0.631 |
| EM CD8^+^ (%) | 15 | 24.5 | 21.1; 39.4 | 16 | 34.9 | 23.1; 41.9 | 26 | 31.0 | 22.9; 45.9 | 0.561 |
| EM CD8^+^CD27^+^ (%) | 15 | 12.0 | 8.9; 16.5 | 16 | 14.8 | 9.2; 18.2 | 26 | 9.7 | 5.3; 14.4 | 0.125 |
| EM CD8^+^CD28^+^ (%) | 15 | 19.7 | 15.0; 27.3 | 16 | 26.7 | 17.8; 33.7 | 26 | 19.5 | 10.6; 33.7 | 0.460 |
| EM CD8^+^CD27^+^CD28^+^ (%) | 15 | 10.9 | 8.2; 13.8 | 16 | 13.4 | 8.4; 15.9 | 26 | 8.2 | 4.8; 14.0 | 0.137 |
| EM CD8^+^CD27^-^CD28^-^ (%) | 15 | 3.6 | 2.4; 8.1 | 16 | 4.3 | 2.8; 8.7 | 26 | 5.2 | 2.9; 10.9 | 0.436 |
| EM CD8^+^CD57^+^ (%) | 15 | 5.8 | 3.1; 9.7 | 16 | 7.2 | 3.8; 13.0 | 26 | 8.0 | 4.3; 13.1 | 0.697 |
| TEMRA CD8^+^ (%) | 15 | 39.9 | 31.5; 52.3 | 16 | 34.6 | 16.3; 42.9 | 26 | 40.3 | 30.1; 68.5 | 0.259 |
| TEMRA CD8^+^CD27^+^ (%) | 15 | 9.0 | 5.1; 13.7 | 16 | 6.0 | 4.8; 9.0 | 26 | 5.9 | 3.1; 9.8 | 0.130 |
| TEMRA CD8^+^CD28^+^ (%) | 15 | 11.3 | 6.6; 15.9 | 16 | 9.7 | 6.2; 13.0 | 26 | 9.2 | 6.3; 21.3 | 0.778 |
| TEMRA CD8^+^CD27^+^CD28^+^ (%) | 15 | 5.0 | 6.3; 21.3 | 16 | 3.9 | 3.0; 5.1 | 26 | 4.3 | 2.1; 5.9 | 0.256 |
| TEMRA CD8^+^CD27^-^CD28^-^ (%) | 15 | 26.2 | 12.3; 34.3 | 16 | 17.5 | 9.0; 25.2 | 26 | 28.8 | 12.8; 47.2 | 0.207 |
| TEMRA CD8^+^CD57^+^ (%) | 15 | 23.0 | 12.3; 29.2 | 16 | 12.7 | 7.7; 26.9 | 26 | 24.1 | 11.6; 33.4 | 0.334 |
| ***T-cell p16^INK4a^ expression*** | | | | | | | | | | |
| *p16^INK4a^* (CNRQ) | 12 | 0.4 | 0.2; 0.7 | 10 | 0.5 | 0.0; 0.8 | 21 | 1.2 | 0.6; 2.2 | 0.014 |
| ***Plasma circulating miRs*** | | | | | | | | | | |
| let-7e (CNRQ) | 15 | 0.7 | 0.4; 1.2 | 19 | 1.0 | 0.6; 1.7 | 31 | 1.3 | 0.7; 2.2 | 0.119 |
| let-7i (CNRQ) | 15 | 1.0 | 0.7; 1.6 | 19 | 0.9 | 0.8; 1.3 | 31 | 1.0 | 0.9; 1.2 | 0.914 |
| miR-9 (CNRQ) | 15 | 0.4 | 0.0; 0.6 | 19 | 0.0 | 0.0; 1.8 | 31 | 0.0 | 0.0; 0.0 | 0.065 |
| miR-17 (CNRQ) | 15 | 1.1 | 0.8; 1.4 | 19 | 1.2 | 0.9; 1.6 | 31 | 1.1 | 0.6; 1.4 | 0.502 |
| miR-18a (CNRQ) | 15 | 1.3 | 0.9; 1.5 | 19 | 1.3 | 0.8; 1.5 | 31 | 0.8 | 0.6; 1.0 | < 0.001 |
| miR-19a (CNRQ) | 15 | 1.2 | 0.9; 1.6 | 19 | 1.3 | 0.7; 1.9 | 31 | 1.0 | 0.7; 1.4 | 0.134 |
| miR-19b (CNRQ) | 15 | 1.0 | 0.9; 1.5 | 19 | 1.3 | 1.0; 1.8 | 31 | 0.8 | 0.6; 1.0 | < 0.001 |
| miR-20a (CNRQ) | 15 | 0.9 | 0.7; 1.2 | 19 | 1.3 | 1.0; 1.6 | 31 | 0.9 | 0.6; 1.3 | 0.002 |
| miR-21 (CNRQ) | 15 | 1.1 | 0.7; 1.3 | 19 | 1.0 | 0.9; 1.5 | 31 | 0.9 | 0.7; 1.2 | 0.177 |
| miR-92a (CNRQ) | 15 | 0.8 | 0.6; 0.8 | 19 | 1.3 | 0.7; 1.6 | 31 | 1.1 | 0.7; 1.5 | 0.056 |
| miR-125b (CNRQ) | 15 | 1.3 | 0.7; 1.4 | 19 | 1.2 | 0.8; 1.5 | 31 | 0.9 | 0.7; 1.2 | 0.099 |
| miR-126 (CNRQ) | 15 | 0.9 | 0.8; 1.2 | 19 | 1.2 | 1.0; 2.2 | 31 | 0.9 | 0.6; 1.3 | 0.149 |
| miR-146a (CNRQ) | 15 | 1.0 | 0.4; 1.7 | 19 | 1.0 | 0.5; 1.7 | 31 | 0.9 | 0.6; 1.7 | 0.943 |
| miR-150 (CNRQ) | 15 | 0.8 | 0.6; 1.4 | 19 | 1.3 | 0.8; 1.6 | 31 | 1.0 | 0.6; 1.3 | 0.087 |
| miR-155 (CNRQ) | 15 | 0.3 | 0.2; 0.7 | 19 | 1.0 | 0.6; 2.2 | 31 | 1.6 | 0.9; 2.4 | < 0.001 |
| miR-181a (CNRQ) | 15 | 0.8 | 0.7; 1.0 | 19 | 1.5 | 0.8; 2.3 | 31 | 0.9 | 0.7; 1.3 | 0.093 |
| miR-195 (CNRQ) | 15 | 1.0 | 0.6; 2.1 | 19 | 1.4 | 0.9; 2.4 | 31 | 0.7 | 0.5; 1.3 | 0.034 |
| miR-223 (CNRQ) | 15 | 0.8 | 0.6; 1.2 | 19 | 1.2 | 0.6; 1.9 | 31 | 0.9 | 0.7; 1.7 | 0.399 |
| miR-326 (CNRQ) | 15 | 0.0 | 0.0; 0.0 | 19 | 0.0 | 0.0; 0.0 | 31 | 0.8 | 0.4; 1.4 | < 0.001 |
| miR-424 (CNRQ) | 15 | 1.5 | 0.8; 2.3 | 19 | 0.9 | 0.3; 1.9 | 31 | 1.4 | 0.6; 2.3 | 0.300 |
