# Supplementary material for: Age‐related remodelling of the blood immunological portrait and the local tumor immune response in patients with luminal breast cancer
Source: Clin Transl Immunology. 2020 Oct 3;9(10):e1184. doi: 10.1002/cti2.1184 (PMC7532981; doi:10.1002/cti2.1184)
Supplement: Supplementary file 4 [file CTI2-9-e1184-s004.docx]

*Supplementary table 3 - Differences in spatial distribution of the tumor infiltrating immune cells.* ***(a)****: The P-values for interaction between region and group (age group or G8 group) are presented.* ***(b)****: Irrespective of the interaction test, differences between age groups and G8 group by regions are shown. Difference >0 means higher proportion in tumor center than invasive front. Difference <0 means lower proportion in tumor center than invasive front. Linear mixed models were used for data analysis. Significance threshold was set below 5% (marked in grey).*

| **(a)** | *P*-values for interaction | |
| --- | --- | --- |
| Marker | Age | G8 |
| CD3 | 0.619 | 0.195 |
| CD4 | 0.687 | 0.895 |
| CD5 | 0.529 | 0.918 |
| CD8 | 0.707 | 0.239 |
| CD20 | 0.612 | 0.708 |
| FOXP3 | 0.856 | 0.458 |

| **(b)** | **Young (35-45 years)** | | **Middle (55-65 years)** | | **Old (≥70 years)** | | **G8 > 14 (‘fit’)** | | **G8 ≤ 14 (‘frail’)** | |
| --- | --- | --- | --- | --- | --- | --- | --- | --- | --- | --- |
| Marker | Difference | *P*-value | Difference | *P*-value | Difference | *P*-value | Difference | *P*-value | Difference | *P*-value |
| CD3 | -3.4 | 0.095 | -1.5 | 0.375 | -1.0 | 0.481 | 0.2 | 0.934 | -4.5 | 0.135 |
| CD4 | -4.6 | 0.013 | -2.6 | 0.102 | -3.0 | 0.020 | -2.9 | 0.114 | -3.3 | 0.198 |
| CD5 | -6.6 | 0.005 | -3.8 | 0.052 | -3.7 | 0.023 | -3.9 | 0.079 | -3.5 | 0.254 |
| CD8 | 1.3 | 0.399 | 0.4 | 0.749 | 1.8 | 0.090 | 0.7 | 0.645 | 3.9 | 0.083 |
| CD20 | -9.3 | < 0.001 | -6.5 | 0.002 | -8.7 | < 0.001 | -10.1 | < 0.001 | -8.5 | 0.020 |
| FOXP3 | 3.4 | 0.009 | 3.9 | < 0.001 | 4.2 | < 0.001 | 3.6 | 0.002 | 5.0 | 0.002 |
