# Supplementary material for: Age‐related remodelling of the blood immunological portrait and the local tumor immune response in patients with luminal breast cancer
Source: Clin Transl Immunology. 2020 Oct 3;9(10):e1184. doi: 10.1002/cti2.1184 (PMC7532981; doi:10.1002/cti2.1184)
Supplement: Supplementary file 5 [file CTI2-9-e1184-s005.docx]

*Supplementary table 4: Blood immune/senescence markers (plasma protein biomarkers, PBMC subset profiling, T-cell p16^INK4a^ expression and plasma circulating miRs) in relation to G8 score (G8 > 14: ‘fit’ and G8 ≤ 14: ‘frail’). The number of measurements (N), median, inter quartile range (IQR) and the P-values are reported. The P-values were calculated via the Mann-Whitney U test, significance threshold was set below 5% (marked in grey).*

|  | **G8 > 14 (‘fit’)** | | | **G8 ≤ 14 (‘frail’)** | | | *P*-value |
| --- | --- | --- | --- | --- | --- | --- | --- |
|  | N | Median | IQR | N | Median | IQR |  |
| ***Plasma protein biomarkers*** | | | | | | | |
| IL-1α (pg/mL) | 19 | 16.4 | 12.5; 19.4 | 10 | 16.1 | 15.6; 20.3 | 0.435 |
| IL-17A (pg/mL) | 19 | 0.4 | 0.1; 0.7 | 10 | 0.7 | 0.2; 1.4 | 0.335 |
| IL-1β (pg/mL) | 19 | 11.1 | 3.4; 20.6 | 10 | 13.0 | 5.2; 22.4 | 0.713 |
| IL-27 (pg/mL) | 19 | 7.9 | 4.4; 8.7 | 10 | 7.1 | 4.0; 9.2 | 0.982 |
| TNFα (pg/mL) | 19 | 3.8 | 1.3; 5.6 | 10 | 3.9 | 1.3; 6.9 | 0.747 |
| IFNγ (pg/mL) | 19 | 9.5 | 5.9; 13.5 | 10 | 9.2 | 4.1; 18.5 | 0.982 |
| IL-12p70 (pg/mL) | 19 | 8.7 | 3.3; 12.8 | 10 | 8.6 | 3.6; 13.4 | 0.801 |
| IL-6 (pg/mL) | 19 | 8.8 | 4.1; 15.7 | 10 | 11.0 | 5.6; 14.1 | 0.927 |
| IL-10 (pg/mL) | 19 | 7.3 | 2.8; 12.0 | 10 | 7.9 | 2.8; 13.1 | 0.890 |
| IL-17F (pg/mL) | 19 | 0.0 | 0.0; 0.0 | 10 | 0.0 | 0.0; 0.0 | 0.514 |
| IP-10 (pg/mL) | 19 | 67.1 | 57.9; 83.1 | 10 | 81.8 | 58.7; 95.9 | 0.176 |
| IL-8 (pg/mL) | 19 | 22.0 | 19.2; 26.8 | 10 | 21.5 | 14.5; 27.3 | 0.748 |
| MCP-1 (pg/mL) | 19 | 130.4 | 103.9; 163.6 | 10 | 122.1 | 97.3; 145.2 | 0.663 |
| Gal-9 (ng/mL) | 19 | 53.5 | 38.7; 80.9 | 10 | 77.2 | 66.6; 89.0 | 0.063 |
| sCD25 (pg/mL) | 19 | 634.8 | 576.5; 1059.8 | 10 | 866.7 | 737.8; 1571.2 | 0.070 |
| TIM-3 (ng/mL) | 19 | 7.1 | 3.6; 11.0 | 10 | 8.2 | 5.1; 11.2 | 0.347 |
| 4-1BB (pg/mL) | 19 | 0.0 | 0.0; 0.0 | 10 | 0.0 | 0.0; 0.0 | 0.566 |
| PD-L1 (pg/mL) | 19 | 1.2 | 0.8; 1.7 | 10 | 1.0 | 0.9; 1.6 | 0.909 |
| PD-1 (pg/mL) | 19 | 0.3 | 0.1; 0.5 | 10 | 0.3 | 0.2; 0.4 | 0.854 |
| CD86 (pg/mL) | 19 | 107.5 | 83.6; 158.9 | 10 | 141.3 | 73.7; 188.2 | 0.422 |
| Free active TGF-β1 (pg/mL) | 19 | 0.0 | 0.0; 0.0 | 10 | 0.0 | 0.0; 0.0 | 1.000 |
| CTLA-4 (pg/mL) | 19 | 0.0 | 0.0; 0.0 | 10 | 0.0 | 0.0; 0.0 | 0.931 |
| LAG-3 (pg/mL) | 19 | 406.7 | 276.7; 735.2 | 10 | 503.4 | 397.9; 866.3 | 0.347 |
| sCD27 (ng/mL) | 19 | 36.4 | 25.3; 55.2 | 10 | 41.4 | 33.3; 47.7 | 0.630 |
| PD-L2 (ng/mL) | 19 | 8.5 | 5.7; 10.26 | 10 | 7.8 | 6.9; 10.2 | 0.766 |
| CRP (ng/mL) | 19 | 1113.3 | 420.1; 1431.5 | 10 | 685.2 | 348.8; 1334.2 | 0.347 |
| IGF-1 (pg/mL) | 19 | 81.9 | 69.2; 96.3 | 10 | 84.9 | 54.0; 114.9 | 0.731 |
| ***PBMC subset profiling*** | | | | | | | |
| CD3^+^ cells (%) | 17 | 48.4 | 42.9; 53.6 | 8 | 51.8 | 46.8; 61.0 | 0.232 |
| CD4^+^ cells (%) | 17 | 25.5 | 20.4; 37.2 | 8 | 23.1 | 15.6; 35.9 | 0.620 |
| CD8^+^ cells (%) | 17 | 13.2 | 10.0; 19.6 | 8 | 19.7 | 13.4; 29.9 | 0.256 |
| CD4/CD8 ratio (%) | 17 | 1.8 | 0.7; 3.6 | 8 | 1.1 | 0.5; 2.7 | 0.398 |
| Total Treg cells (%) | 17 | 1.6 | 0.6; 2.1 | 8 | 0.8 | 0.4; 1.3 | 0.256 |
| Naive Treg cells (%) | 17 | 65.2 | 57.4; 73.1 | 8 | 68.5 | 59.8; 78.6 | 0.466 |
| Memory Treg cells (%) | 17 | 33.2 | 20.3; 37.4 | 8 | 30.9 | 20.7; 39.5 | 0.884 |
| CD3^+^CD16^+^ cells (%) | 17 | 1.8 | 1.3; 3.4 | 8 | 6.0 | 5.7; 8.4 | < 0.001 |
| Total NK cells (%) | 17 | 7.6 | 3.5; 12.8 | 8 | 10.0 | 8.5; 13.9 | 0.336 |
| CD56^bright^CD16^-^ NK cells (%) | 17 | 5.1 | 4.4; 6.6 | 8 | 3.4 | 3.1; 4.5 | 0.123 |
| CD56^dim^CD16^+^ NK Cells (%) | 17 | 90.8 | 87.1; 91.8 | 8 | 94.6 | 87.6; 95.5 | 0.067 |
| B-cells (%) | 17 | 11.6 | 7.4; 12.6 | 8 | 8.3 | 6.6; 9.2 | 0.308 |
| Naive B-cells (%) | 17 | 46.4 | 31.9; 67.9 | 8 | 40.7 | 37.2; 50.0 | 0.793 |
| Non-switched memory (%) | 17 | 16.2 | 8.5; 29.3 | 8 | 19.9 | 13.3; 20.6 | 0.838 |
| Class-switched memory (%) | 17 | 23.0 | 16.6; 32.5 | 8 | 36.9 | 16.4; 43.4 | 0.171 |
| Monocytes (%) | 17 | 14.6 | 7.8; 18.0 | 8 | 15.1 | 12.0; 19.5 | 0.793 |
| Classical monocytes (%) | 17 | 81.5 | 76.3; 85.7 | 8 | 83.8 | 74.0; 88.1 | 0.838 |
| Intermediate monocytes (%) | 17 | 9.2 | 7.1; 11.3 | 8 | 9.2 | 6.0; 12.7 | 0.977 |
| Non-classical monocytes (%) | 17 | 8.8 | 4.9; 10.8 | 8 | 7.8 | 4.5; 15.4 | 0.662 |
| Total pDC (%) | 17 | 0.1 | 0.1; 0.4 | 8 | 0.1 | 0.0; 0.2 | 0.281 |
| Total mDC (%) | 17 | 1.0 | 0.6; 1.4 | 8 | 0.9 | 0.5; 1.3 | 0.884 |
| Total HSC (%) | 17 | 0.1 | 0.0; 0.1 | 8 | 0.1 | 0.0; 0.1 | 0.367 |
| CD4^+^CD27^+^ (%) | 17 | 70.9 | 55.9; 77.8 | 8 | 68.2 | 47.4; 80.8 | 0.793 |
| CD4^+^CD28^+^ (%) | 17 | 98.7 | 86.6; 99.3 | 8 | 91.3 | 57.5; 97.3 | 0.109 |
| CD4^+^CD27^+^CD28^+^ (%) | 17 | 70.9 | 54.7; 77.2 | 8 | 64.7 | 38.9; 76.5 | 0.432 |
| CD4^+^CD27^-^CD28^-^ (%) | 17 | 1.2 | 0.4; 13.3 | 8 | 8.3 | 2.5; 38.9 | 0.086 |
| CD4^+^CD57^+^ (%) | 17 | 4.8 | 2.0; 17.4 | 8 | 8.8 | 5.7; 40.8 | 0.109 |
| CD4^+^ Tregs (%) | 17 | 4.0 | 3.2; 6.5 | 8 | 3.1 | 1.4; 4.2 | 0.097 |
| Naive CD4^+^ (%) | 17 | 30.3 | 22.1; 39.0 | 8 | 37.1 | 20.4; 46.6 | 0.884 |
| Naive CD4^+^CD27^+^ (%) | 17 | 27.2 | 20.0; 34.8 | 8 | 36.0 | 21.8; 44.9 | 0.749 |
| Naive CD4^+^CD28^+^ (%) | 17 | 30.0 | 21.6; 38.7 | 8 | 36.9 | 14.7; 46.3 | 0.930 |
| Naive CD4^+^CD27^+^CD28^+^ (%) | 17 | 27.2 | 19.6; 34.7 | 8 | 35.9 | 11.8; 44.8 | 0.977 |
| Naive CD4^+^CD27^-^CD28^-^ (%) | 17 | 0.2 | 0.1; 0.5 | 8 | 0.2 | 0.1; 2.4 | 0.977 |
| Naive CD4^+^CD57^+^ (%) | 17 | 0.8 | 0.2; 2.4 | 8 | 1.1 | 0.7; 4.2 | 0.281 |
| CM CD4^+^ (%) | 17 | 17.0 | 11.2; 29.9 | 8 | 12.3 | 10.9; 16.0 | 0.210 |
| CM CD4^+^CD27^+^ (%) | 17 | 12.7 | 10.1; 26.2 | 8 | 10.3 | 8.2; 13.3 | 0.123 |
| CM CD4^+^CD28^+^ (%) | 17 | 16.7 | 11.2; 29.8 | 8 | 12.1 | 10.3; 16.0 | 0.190 |
| CM CD4^+^CD27^+^CD28^+^ (%) | 17 | 12.7 | 10.1; 26.2 | 8 | 10.3 | 8.2; 13.3 | 0.137 |
| CM CD4^+^CD27^-^CD28^-^ (%) | 17 | 0.0 | 0.0; 0.0 | 8 | 0.0 | 0.0; 0.2 | 0.323 |
| CM CD4^+^CD57^+^ (%) | 17 | 0.2 | 0.1; 0.4 | 8 | 0.2 | 0.1; 0.5 | 0.705 |
| EM CD4^+^ (%) | 17 | 27.6 | 22.0; 34.8 | 8 | 33.1 | 29.3; 36.4 | 0.210 |
| EM CD4^+^CD27^+^ (%) | 17 | 11.8 | 9.0; 18.2 | 8 | 13.4 | 4.8; 21.0 | 0.749 |
| EM CD4^+^CD28^+^ (%) | 17 | 25.5 | 18.4; 32.3 | 8 | 27.4 | 14.7; 32.8 | 0.884 |
| EM CD4^+^CD27^+^CD28^+^ (%) | 17 | 11.8 | 9.0; 18.1 | 8 | 13.2 | 4.6; 20.9 | 0.793 |
| EM CD4^+^CD27^-^CD28^-^ (%) | 17 | 0.3 | 0.1; 4.6 | 8 | 3.1 | 0.3; 12.2 | 0.153 |
| EM CD4^+^CD57^+^ (%) | 17 | 1.3 | 0.6; 4.5 | 8 | 6.5 | 1.5; 8.1 | 0.058 |
| TEMRA CD4^+^ (%) | 17 | 9.2 | 4.5; 21.6 | 8 | 14.2 | 8.3; 23.0 | 0.281 |
| TEMRA CD4^+^CD27^+^ (%) | 17 | 3.7 | 1.9; 4.6 | 8 | 4.4 | 1.4; 7.5 | 0.816 |
| TEMRA CD4^+^CD28^+^ (%) | 17 | 5.1 | 3.1; 9.4 | 8 | 5.3 | 1.8; 10.8 | 0.884 |
| TEMRA CD4^+^CD27^+^CD28^+^ (%) | 17 | 3.2 | 1.8; 4.6 | 8 | 4.4 | 1.0; 7.1 | 0.793 |
| TEMRA CD4^+^CD27^-^CD28^-^ (%) | 17 | 0.5 | 0.2; 5.7 | 8 | 2.1 | 0.7; 18.3 | 0.154 |
| TEMRA CD4^+^CD57^+^ (%) | 17 | 0.6 | 0.3; 5.7 | 8 | 2.6 | 0.9; 18.4 | 0.076 |
| CD8^+^CD27^+^ (%) | 17 | 47.6 | 21.4; 55.1 | 8 | 22.0 | 12.6; 43.2 | 0.044 |
| CD8^+^CD28^+^ (%) | 17 | 72.3 | 47.1; 79.9 | 8 | 39.2 | 22.1; 61.6 | 0.016 |
| CD8^+^CD27^+^CD28^+^ (%) | 17 | 45.0 | 19.2; 50.7 | 8 | 19.3 | 9.4; 36.8 | 0.029 |
| CD8^+^CD27^-^CD28^-^ (%) | 17 | 27.3 | 16.6; 51.3 | 8 | 53.0 | 37.1; 74.7 | 0.013 |
| CD8^+^CD57^+^ (%) | 17 | 26.9 | 19.1; 34.9 | 8 | 48.5 | 38.4; 62.0 | 0.007 |
| Naive CD8^+^ (%) | 17 | 10.5 | 6.8; 16.8 | 8 | 6.1 | 3.5; 14.7 | 0.308 |
| Naive CD8^+^CD27^+^ (%) | 17 | 8.1 | 3.4; 13.6 | 8 | 4.6 | 0.7; 11.3 | 0.171 |
| Naive CD8^+^CD28^+^ (%) | 17 | 8.3 | 4.3; 16.1 | 8 | 5.0 | 1.1; 12.1 | 0.171 |
| Naive CD8^+^CD27^+^CD28^+^ (%) | 17 | 7.8 | 3.4; 13.4 | 8 | 4.0 | 0.6; 11.2 | 0.171 |
| Naive CD8^+^CD27^-^CD28^-^ (%) | 17 | 0.3 | 0.2; 1.1 | 8 | 0.9 | 0.6; 2.2 | 0.097 |
| Naive CD8^+^CD57^+^ (%) | 17 | 1.0 | 0.2; 3.1 | 8 | 1.5 | 0.8; 2.0 | 0.541 |
| CM CD8^+^ (%) | 17 | 8.8 | 5.5; 13.7 | 8 | 5.8 | 1.9; 6.2 | 0.086 |
| CM CD8^+^CD27^+^ (%) | 17 | 6.1 | 3.6; 11.2 | 8 | 3.4 | 1.2; 4.7 | 0.097 |
| CM CD8^+^CD28^+^ (%) | 17 | 8.5 | 5.1; 13.4 | 8 | 5.2 | 1.5; 6.1 | 0.109 |
| CM CD8^+^CD27^+^CD28^+^ (%) | 17 | 5.9 | 3.5; 11.0 | 8 | 3.4 | 1.1; 4.6 | 0.123 |
| CM CD8^+^CD27^-^CD28^-^ (%) | 17 | 0.2 | 0.1; 0.5 | 8 | 0.2 | 0.0; 0.5 | 0.620 |
| CM CD8^+^CD57^+^ (%) | 17 | 0.6 | 0.2; 1.0 | 8 | 0.5 | 0.2; 0.8 | 0.580 |
| EM CD8^+^ (%) | 17 | 31.1 | 25.0; 50.4 | 8 | 28.1 | 15.8; 33.4 | 0.398 |
| EM CD8^+^CD27^+^ (%) | 17 | 10.3 | 6.6; 20.7 | 8 | 9.7 | 4.6; 12.4 | 0.432 |
| EM CD8^+^CD28^+^ (%) | 17 | 26.0 | 10.9; 37.7 | 8 | 13.1 | 8.6; 23.1 | 0.210 |
| EM CD8^+^CD27^+^CD28^+^ (%) | 17 | 9.6 | 5.7; 17.3 | 8 | 7.6 | 4.1; 11.7 | 0.322 |
| EM CD8^+^CD27^-^CD28^-^ (%) | 17 | 3.9 | 2.8; 10.6 | 8 | 7.5 | 4.2; 10.6 | 0.281 |
| EM CD8^+^CD57^+^ (%) | 17 | 5.7 | 4.2; 13.0 | 8 | 8.6 | 5.6; 12.4 | 0.432 |
| TEMRA CD8^+^ (%) | 17 | 35.9 | 23.3; 51.6 | 8 | 63.6 | 35.8; 70.5 | 0.097 |
| TEMRA CD8^+^CD27^+^ (%) | 17 | 7.1 | 5.3; 10.1 | 8 | 3.1 | 2.4; 6.2 | 0.058 |
| TEMRA CD8^+^CD28^+^ (%) | 17 | 9.2 | 7.9; 22.6 | 8 | 6.1 | 5.0; 11.8 | 0.171 |
| TEMRA CD8^+^CD27^+^CD28^+^ (%) | 17 | 5.0 | 4.1; 6.3 | 8 | 2.3 | 1.8; 3.3 | 0.044 |
| TEMRA CD8^+^CD27^-^CD28^-^ (%) | 17 | 17.6 | 11.8; 32.7 | 8 | 44.8 | 29.6; 60.4 | 0.013 |
| TEMRA CD8^+^CD57^+^ (%) | 17 | 19.1 | 11.1; 28.6 | 8 | 36.0 | 26.8; 47.5 | 0.005 |
| ***T-cell p16^INK4a^ expression*** | | | | | | | |
| *p16^INK4a^* (CNRQ) | 14 | 0.9 | 0.5; 1.2 | 6 | 3.2 | 1.7; 5.3 | 0.043 |
| ***Plasma circulating miRs*** | | | | | | | |
| let-7e (CNRQ) | 19 | 1.3 | 0.6; 2.8 | 10 | 0.9 | 0.7; 1.8 | 0.224 |
| let-7i (CNRQ) | 19 | 1.0 | 0.8; 1.2 | 10 | 1.0 | 0.9; 1.5 | 0.836 |
| miR-9 (CNRQ) | 19 | 0.0 | 0.0; 0.4 | 10 | 0.0 | 0.0; 0.0 | 0.056 |
| miR-17 (CNRQ) | 19 | 1.1 | 0.5; 1.4 | 10 | 1.3 | 1.0; 1.4 | 0.124 |
| miR-18a (CNRQ) | 19 | 0.8 | 0.6; 1.0 | 10 | 0.7 | 0.0; 1.0 | 0.302 |
| miR-19a (CNRQ) | 19 | 1.0 | 0.7; 1.4 | 10 | 0.9 | 0.4; 1.5 | 0.697 |
| miR-19b (CNRQ) | 19 | 0.8 | 0.6; 1.0 | 10 | 0.8 | 0.7; 1.1 | 0.836 |
| miR-20a (CNRQ) | 19 | 0.7 | 0.5; 1.3 | 10 | 1.0 | 0.9; 1.4 | 0.057 |
| miR-21 (CNRQ) | 19 | 0.9 | 0.7; 1.2 | 10 | 1.0 | 0.7; 1.4 | 0.536 |
| miR-92a (CNRQ) | 19 | 0.8 | 0.6; 1.3 | 10 | 1.2 | 1.0; 1.5 | 0.113 |
| miR-125b (CNRQ) | 19 | 0.9 | 0.7; 1.2 | 10 | 0.9 | 0.6; 1.2 | 0.945 |
| miR-126 (CNRQ) | 19 | 0.8 | 0.6; 1.2 | 10 | 1.2 | 0.6; 1.8 | 0.176 |
| miR-146a (CNRQ) | 19 | 0.9 | 0.6; 1.7 | 10 | 0.9 | 0.6; 1.9 | 0.982 |
| miR-150 (CNRQ) | 19 | 1.0 | 0.6; 1.3 | 10 | 1.1 | 0.8; 1.6 | 0.422 |
| miR-155 (CNRQ) | 19 | 1.4 | 0.8; 2.0 | 10 | 2.2 | 1.5; 2.7 | 0.224 |
| miR-181a (CNRQ) | 19 | 0.9 | 0.7; 1.1 | 10 | 1.0 | 0.8; 1.4 | 0.477 |
| miR-195 (CNRQ) | 19 | 0.6 | 0.4; 1.1 | 10 | 1.1 | 0.6; 1.6 | 0.070 |
| miR-223 (CNRQ) | 19 | 0.9 | 0.5; 1.4 | 10 | 1.5 | 0.8; 2.3 | 0.176 |
| miR-326 (CNRQ) | 19 | 0.8 | 0.4; 1.4 | 10 | 0.7 | 0.0; 1.0 | 0.835 |
| miR-424 (CNRQ) | 19 | 1.4 | 0.7; 2.4 | 10 | 0.8 | 0.4; 1.7 | 0.191 |
