# Supplementary material for: Age‐related remodelling of the blood immunological portrait and the local tumor immune response in patients with luminal breast cancer
Source: Clin Transl Immunology. 2020 Oct 3;9(10):e1184. doi: 10.1002/cti2.1184 (PMC7532981; doi:10.1002/cti2.1184)
Supplement: Supplementary file 6 [file CTI2-9-e1184-s006.docx]

*Supplementary table 5 - Tumor immune infiltrate markers in relation to G8 score (G8 >14: ‘fit’ and G8 ≤ 14: ‘frail’). The percentage of sTILs, CD68 staining grade and the proportion and density of positively stained CD3, CD4, CD5, CD8, CD20 and FOXP3 cells are reported. The proportion of positive lymphocytes was defined as the ratio of positively stained lymphocytes versus the total number of infiltrating lymphocytes counted in that region after digital segmentation. The density of positive lymphocytes was defined as the number of positively stained lymphocytes per mm². The P-values were calculated via the Mann-Whitney U test, significance threshold was set below 5% (marked in grey).*

|  | **G8 > 14 (‘fit’)** | | | **G8 ≤ 14 (‘frail’)** | | | *P*-value |
| --- | --- | --- | --- | --- | --- | --- | --- |
|  | N | Median | IQR | N | Median | IQR |  |
| sTILs (%) | 18 | 6.5 | 2.0; 14.0 | 9 | 9.0 | 3.2; 18.0 | 0.662 |
| CD68 staining grade | 18 | II | I; II | 9 | II | II; II | 0.650 |
| CD3^+^ cells - Tumor center |  |  |  |  |  |  |  |
| Proportion (%) | 18 | 42.4 | 35.1; 53.2 | 8 | 45.2 | 31.3; 51.0 | 0.718 |
| Density (+ cells/mm²) | 18 | 73.7 | 29.4; 139.0 | 8 | 102.5 | 48.2; 212.6 | 0.420 |
| CD3^+^ cells - Invasive front |  |  |  |  |  |  |  |
| Proportion (%) | 18 | 42.5 | 36.2; 48.9 | 8 | 48.2 | 35.3; 55.6 | 0.420 |
| Density (+ cells/mm²) | 18 | 184.8 | 99.0; 398.8 | 8 | 174.1 | 116.2; 429.7 | 0.803 |
| CD3^+^ cells - Whole tumor |  |  |  |  |  |  |  |
| Proportion (%) | 18 | 43.7 | 34.6; 49.5 | 8 | 46.1 | 36.7; 52.7 | 0.760 |
| Density (+ cells/mm²) | 18 | 120.0 | 66.0; 229.3 | 8 | 144.8 | 94.5; 262.8 | 0.453 |
| CD4^+^ cells - Tumor center |  |  |  |  |  |  |  |
| Proportion (%) | 18 | 27.7 | 19.8; 33.6 | 9 | 29.6 | 24.6; 34.3 | 0.341 |
| Density (+ cells/mm²) | 18 | 39.9 | 12.0; 84.6 | 9 | 83.9 | 40.2; 214.3 | 0.143 |
| CD4^+^ cells - Invasive front |  |  |  |  |  |  |  |
| Proportion (%) | 18 | 30.2 | 24.3; 37.9 | 9 | 33.0 | 31.8; 41.3 | 0.173 |
| Density (+ cells/mm²) | 18 | 185.2 | 79.3; 312.4 | 9 | 180.5 | 130.8; 675.8 | 0.487 |
| CD4^+^ cells - Whole tumor |  |  |  |  |  |  |  |
| Proportion (%) | 18 | 30.8 | 23.6; 36.8 | 9 | 31.5 | 30.4; 34.8 | 0.625 |
| Density (+ cells/mm²) | 18 | 86.1 | 33.8; 165.6 | 9 | 110.1 | 77.0; 306.2 | 0.247 |
| CD5^+^ cells - Tumor center |  |  |  |  |  |  |  |
| Proportion (%) | 18 | 30.8 | 22.6; 44.0 | 9 | 45.9 | 32.0; 50.7 | 0.060 |
| Density (+ cells/mm²) | 18 | 52.1 | 19.7; 111.2 | 9 | 108.8 | 64.9; 247.8 | 0.076 |
| CD5^+^ cells - Invasive front |  |  |  |  |  |  |  |
| Proportion (%) | 18 | 35.6 | 30.2; 39.3 | 9 | 52.9 | 30.5; 55.2 | 0.117 |
| Density (+ cells/mm²) | 18 | 161.4 | 79.9; 289.0 | 9 | 197.6 | 128.7; 441.1 | 0.396 |
| CD5^+^ cells - Whole tumor |  |  |  |  |  |  |  |
| Proportion (%) | 18 | 33.5 | 29.8; 42.0 | 9 | 46.5 | 31.2; 52.5 | 0.105 |
| Density (+ cells/mm²) | 18 | 84.8 | 46.9; 170.0 | 9 | 132.5 | 121.9; 319.4 | 0.269 |
| CD8^+^ cells - Tumor center |  |  |  |  |  |  |  |
| Proportion (%) | 18 | 19.1 | 15.5; 27.2 | 9 | 22.8 | 21.6; 29.1 | 0.247 |
| Density (+ cells/mm²) | 18 | 33.9 | 25.4; 83.5 | 9 | 80.9 | 67.8; 117.7 | 0.095 |
| CD8^+^ cells - Invasive front |  |  |  |  |  |  |  |
| Proportion (%) | 18 | 20.0 | 14.2; 28.4 | 9 | 20.4 | 17.4; 23.1 | 0.979 |
| Density (+ cells/mm²) | 18 | 107.9 | 52.3; 170.6 | 9 | 101.3 | 78.0; 241.3 | 0.589 |
| CD8^+^ cells - Whole tumor |  |  |  |  |  |  |  |
| Proportion (%) | 18 | 20.7 | 15.0; 26.1 | 9 | 22.2 | 19.5; 25.1 | 0.738 |
| Density (+ cells/mm²) | 18 | 67.3 | 39.7; 110.7 | 9 | 81.6 | 72.8; 170.5 | 0.341 |
| CD20^+^ cells - Tumor center |  |  |  |  |  |  |  |
| Proportion (%) | 18 | 8.8 | 4.4; 13.6 | 9 | 8.6 | 6.1; 14.4 | 0.979 |
| Density (+ cells/mm²) | 18 | 7.9 | 2.5; 28.8 | 9 | 17.7 | 4.3; 68.3 | 0.487 |
| CD20^+^ cells - Invasive front |  |  |  |  |  |  |  |
| Proportion (%) | 18 | 16.6 | 12.5; 28.0 | 9 | 17.2 | 9.2; 25.4 | 0.700 |
| Density (+ cells/mm²) | 18 | 62.3 | 17.9; 209.6 | 9 | 56.5 | 37.3; 127.2 | 0.898 |
| CD20^+^ cells - Whole tumor |  |  |  |  |  |  |  |
| Proportion (%) | 18 | 14.4 | 9.3; 25.8 | 9 | 12.8 | 8.7; 21.5 | 0.520 |
| Density (+ cells/mm²) | 18 | 38.8 | 14.7; 78.7 | 9 | 30.6 | 10.5; 126.2 | 0.898 |
| FOXP3^+^ cells - Tumor center |  |  |  |  |  |  |  |
| Proportion (%) | 18 | 8.4 | 3.1; 11.8 | 9 | 11.6 | 9.6; 17.6 | 0.060 |
| Density (+ cells/mm²) | 18 | 7.2 | 4.0; 24.5 | 9 | 34.2 | 12.3; 40.9 | 0.037 |
| FOXP3^+^ cells - Invasive front |  |  |  |  |  |  |  |
| Proportion (%) | 18 | 4.4 | 2.4; 7.2 | 9 | 8.5 | 7.9; 9.9 | 0.015 |
| Density (+ cells/mm²) | 18 | 17.0 | 6.7; 61.4 | 9 | 31.9 | 21.5; 75.5 | 0.173 |
| FOXP3^+^ cells - Whole tumor |  |  |  |  |  |  |  |
| Proportion (%) | 18 | 5.7 | 2.8; 9.0 | 9 | 10.6 | 9.5; 13.1 | 0.007 |
| Density (+ cells/mm²) | 18 | 12.3 | 3.9; 38.2 | 9 | 30.4 | 14.8; 47.5 | 0.129 |
| CD8/CD3 ratio |  |  |  |  |  |  |  |
| Tumor center | 18 | 0.6 | 0.5; 0.9 | 8 | 0.6 | 0.3; 1.0 | 0.892 |
| Invasive front | 18 | 0.5 | 0.4; 0.7 | 8 | 0.5 | 0.4; 0.7 | 0.849 |
| Whole tumor | 18 | 0.6 | 0.4; 0.8 | 8 | 0.5 | 0.4; 0.6 | 0.531 |
