# Supplementary material for: Age‐related remodelling of the blood immunological portrait and the local tumor immune response in patients with luminal breast cancer
Source: Clin Transl Immunology. 2020 Oct 3;9(10):e1184. doi: 10.1002/cti2.1184 (PMC7532981; doi:10.1002/cti2.1184)
Supplement: Supplementary file 7 [file CTI2-9-e1184-s007.docx]

*Supplementary table 6 - Correlations between the blood immune/senescence (plasma protein biomarkers, PBMC subset profiling, T-cell p16^INK4a^ expression and plasma circulating miRs) and tumor immune infiltrate markers (sTILs %, CD68 staining grade, density of CD3^+^, CD4^+^, CD5^+^, CD8^+^, CD20^+^ and FOXP3^+^ cells). Rho and the p-values are reported, the P-values were obtained by using the Spearman correlations test, significance threshold was set below 5% (marked in grey). The color scale indicates the direction and strength of the correlation. Values marked in green indicate a negative correlation; values marked in red indicate a positive correlation.*

*
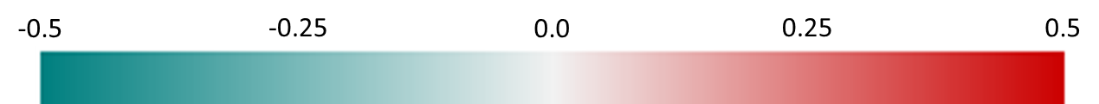
*

|  |  |  |  |  | **Density in whole tumor** | | | | | | | | | | | |
| --- | --- | --- | --- | --- | --- | --- | --- | --- | --- | --- | --- | --- | --- | --- | --- | --- |
|  | **sTILs (%)** | | **CD68 grade** | | **CD3^+^ cells** | | **CD4^+^ cells** | | **CD5^+^ cells** | | **CD8^+^ cells** | | **CD20^+^ cells** | | **FOXP3^+^ cells** | |
|  | Rho | *P*-value | Rho | *P*-value | Rho | *P*-value | Rho | *P*-value | Rho | *P*-value | Rho | *P*-value | Rho | *P*-value | Rho | *P*-value |
| ***Plasma protein biomarkers*** | | | | | | | | | | | | | | | | |
| IL-1α | -0.295 | 0.020 | 0.087 | 0.504 | -0.223 | 0.084 | -0.215 | 0.096 | -0.214 | 0.095 | -0.324 | 0.010 | -0.217 | 0.090 | -0.107 | 0.414 |
| IL-17A | 0.132 | 0.306 | -0.052 | 0.689 | 0.125 | 0.337 | 0.121 | 0.352 | 0.095 | 0.462 | 0.165 | 0.200 | 0.046 | 0.724 | 0.016 | 0.902 |
| IL-1β | -0.036 | 0.784 | -0.048 | 0.713 | 0.043 | 0.744 | 0.056 | 0.666 | 0.052 | 0.686 | 0.061 | 0.637 | 0.032 | 0.805 | 0.048 | 0.716 |
| IL-6 | -0.305 | 0.016 | 0.066 | 0.611 | -0.185 | 0.154 | -0.209 | 0.105 | -0.253 | 0.048 | -0.198 | 0.122 | -0.210 | 0.102 | -0.187 | 0.148 |
| IL-10 | -0.042 | 0.744 | 0.007 | 0.956 | 0.044 | 0.736 | 0.003 | 0.979 | 0.027 | 0.836 | 0.066 | 0.611 | 0.018 | 0.891 | 0.027 | 0.834 |
| IL-12p70 | -0.106 | 0.413 | -0.069 | 0.596 | -0.054 | 0.682 | -0.030 | 0.818 | -0.063 | 0.629 | -0.019 | 0.886 | -0.071 | 0.582 | -0.047 | 0.721 |
| IL-17F | -0.009 | 0.946 | 0.154 | 0.232 | -0.035 | 0.790 | -0.043 | 0.743 | -0.014 | 0.913 | -0.039 | 0.765 | -0.007 | 0.959 | 0.004 | 0.977 |
| IL-27 | -0.128 | 0.320 | 0.104 | 0.420 | -0.044 | 0.734 | -0.040 | 0.758 | -0.067 | 0.603 | -0.055 | 0.673 | -0.109 | 0.401 | -0.057 | 0.661 |
| IFNγ | -0.116 | 0.368 | -0.009 | 0.946 | -0.044 | 0.737 | -0.038 | 0.772 | -0.034 | 0.793 | -0.006 | 0.964 | -0.015 | 0.911 | -0.027 | 0.835 |
| TNFα | -0.082 | 0.525 | -0.019 | 0.884 | -0.021 | 0.874 | -0.025 | 0.846 | -0.026 | 0.841 | -0.010 | 0.939 | -0.027 | 0.833 | -0.011 | 0.930 |
| IP-10 | -0.085 | 0.510 | 0.099 | 0.444 | -0.087 | 0.507 | -0.066 | 0.615 | -0.113 | 0.381 | -0.167 | 0.193 | -0.077 | 0.552 | 0.013 | 0.919 |
| IL-8 | -0.230 | 0.073 | 0.049 | 0.704 | -0.172 | 0.185 | -0.139 | 0.287 | -0.177 | 0.170 | -0.229 | 0.073 | -0.103 | 0.427 | -0.180 | 0.165 |
| MCP-1 | -0.373 | 0.003 | -0.165 | 0.200 | -0.299 | 0.019 | -0.244 | 0.058 | -0.287 | 0.024 | -0.386 | 0.002 | -0.219 | 0.087 | -0.357 | 0.005 |
| Gal-9 | -0.256 | 0.045 | -0.042 | 0.744 | -0.144 | 0.270 | -0.123 | 0.345 | -0.160 | 0.215 | -0.209 | 0.104 | -0.260 | 0.041 | -0.031 | 0.810 |
| sCD25 | -0.204 | 0.112 | -0.192 | 0.136 | -0.106 | 0.416 | -0.128 | 0.327 | -0.152 | 0.239 | -0.144 | 0.265 | -0.186 | 0.148 | -0.073 | 0.575 |
| TIM-3 | -0.334 | 0.008 | -0.320 | 0.011 | -0.198 | 0.126 | -0.174 | 0.179 | -0.243 | 0.057 | -0.275 | 0.030 | -0.270 | 0.034 | -0.180 | 0.165 |
| 4-1BB | 0.073 | 0.573 | 0.131 | 0.309 | 0.220 | 0.088 | 0.139 | 0.285 | 0.159 | 0.218 | 0.127 | 0.325 | 0.213 | 0.097 | 0.126 | 0.332 |
| PD-L1 | 0.144 | 0.265 | 0.073 | 0.571 | 0.246 | 0.056 | 0.195 | 0.132 | 0.197 | 0.124 | 0.173 | 0.179 | 0.227 | 0.077 | 0.145 | 0.266 |
| sCD27 | -0.082 | 0.529 | -0.043 | 0.738 | -0.050 | 0.703 | -0.102 | 0.435 | -0.096 | 0.458 | -0.026 | 0.841 | -0.111 | 0.391 | 0.002 | 0.989 |
| CD86 | -0.116 | 0.368 | -0.042 | 0.748 | 0.030 | 0.817 | 0.071 | 0.588 | 0.061 | 0.637 | -0.008 | 0.950 | 0.081 | 0.532 | 0.020 | 0.880 |
| CTLA-4 | 0.051 | 0.696 | 0.057 | 0.661 | 0.035 | 0.786 | -0.083 | 0.523 | 0.019 | 0.881 | 0.003 | 0.981 | -0.028 | 0.828 | -0.064 | 0.625 |
| Free active TGF-β1 | 0.046 | 0.720 | 0.061 | 0.635 | 0.100 | 0.441 | -0.039 | 0.765 | 0.043 | 0.738 | 0.088 | 0.498 | 0.014 | 0.917 | -0.015 | 0.909 |
| LAG-3 | -0.032 | 0.805 | 0.145 | 0.260 | 0.140 | 0.282 | 0.117 | 0.368 | 0.133 | 0.304 | 0.083 | 0.521 | 0.062 | 0.631 | 0.106 | 0.418 |
| PD-1 | 0.103 | 0.427 | 0.071 | 0.585 | 0.225 | 0.082 | 0.174 | 0.180 | 0.216 | 0.092 | 0.159 | 0.216 | 0.265 | 0.038 | 0.126 | 0.335 |
| PD-L2 | -0.101 | 0.436 | -0.126 | 0.329 | -0.122 | 0.351 | -0.054 | 0.682 | -0.150 | 0.246 | -0.014 | 0.912 | -0.072 | 0.580 | -0.101 | 0.439 |
| CRP | -0.259 | 0.043 | -0.012 | 0.927 | -0.179 | 0.167 | -0.133 | 0.307 | -0.191 | 0.137 | -0.141 | 0.274 | -0.115 | 0.374 | -0.177 | 0.173 |
| IGF-1 | 0.162 | 0.209 | 0.088 | 0.497 | 0.048 | 0.711 | 0.063 | 0.631 | 0.088 | 0.497 | 0.133 | 0.303 | 0.004 | 0.975 | 0.068 | 0.605 |
| ***PBMC subset profiling*** | | | | | | | | | | | | | | | | |
| CD3^+^ cells | -0.088 | 0.527 | 0.097 | 0.486 | 0.035 | 0.801 | 0.052 | 0.714 | 0.020 | 0.887 | -0.107 | 0.443 | -0.085 | 0.540 | 0.024 | 0.867 |
| CD4^+^ cells | -0.250 | 0.068 | 0.070 | 0.617 | -0.117 | 0.402 | -0.087 | 0.537 | -0.149 | 0.282 | -0.268 | 0.050 | -0.199 | 0.150 | -0.174 | 0.212 |
| CD8^+^ cells | 0.265 | 0.053 | -0.097 | 0.484 | 0.259 | 0.061 | 0.218 | 0.116 | 0.237 | 0.085 | 0.239 | 0.081 | 0.183 | 0.185 | 0.310 | 0.024 |
| CD4/CD8 ratio | -0.255 | 0.063 | 0.103 | 0.461 | -0.172 | 0.220 | -0.153 | 0.274 | -0.178 | 0.197 | -0.263 | 0.055 | -0.177 | 0.199 | -0.224 | 0.107 |
| Total Treg cells | -0.325 | 0.016 | -0.092 | 0.508 | -0.251 | 0.070 | -0.189 | 0.174 | -0.223 | 0.105 | -0.287 | 0.036 | -0.211 | 0.126 | -0.280 | 0.042 |
| Naive Treg cells | 0.015 | 0.914 | -0.022 | 0.872 | -0.081 | 0.565 | -0.112 | 0.425 | -0.015 | 0.915 | 0.061 | 0.660 | -0.008 | 0.954 | 0.000 | 1.000 |
| Memory Treg cells | -0.085 | 0.542 | 0.048 | 0.731 | 0.017 | 0.907 | 0.030 | 0.833 | -0.047 | 0.735 | -0.119 | 0.391 | -0.091 | 0.512 | -0.103 | 0.465 |
| CD3^+^CD16^+^ cells | 0.148 | 0.284 | 0.253 | 0.065 | 0.130 | 0.353 | 0.174 | 0.214 | 0.157 | 0.257 | 0.131 | 0.343 | 0.143 | 0.302 | 0.246 | 0.076 |
| Total NK cells | 0.008 | 0.956 | 0.330 | 0.015 | 0.011 | 0.937 | 0.058 | 0.679 | 0.037 | 0.791 | 0.060 | 0.667 | 0.023 | 0.868 | 0.042 | 0.768 |
| CD56^bright^CD16^-^ NK cells | 0.056 | 0.687 | 0.052 | 0.707 | -0.011 | 0.938 | 0.122 | 0.384 | 0.018 | 0.897 | -0.002 | 0.989 | 0.101 | 0.466 | -0.008 | 0.957 |
| CD56^dim^CD16^+^ NK Cells | 0.069 | 0.619 | 0.004 | 0.977 | 0.133 | 0.343 | 0.018 | 0.898 | 0.055 | 0.693 | 0.085 | 0.543 | -0.007 | 0.962 | 0.078 | 0.578 |
| B-cells | -0.155 | 0.264 | -0.245 | 0.075 | -0.151 | 0.281 | -0.251 | 0.070 | -0.203 | 0.140 | -0.163 | 0.240 | -0.101 | 0.466 | -0.075 | 0.593 |
| Naive B-cells | -0.130 | 0.350 | 0.341 | 0.012 | -0.224 | 0.107 | -0.160 | 0.252 | -0.227 | 0.099 | -0.284 | 0.038 | -0.211 | 0.126 | -0.223 | 0.108 |
| Non-switched memory B-cells | 0.042 | 0.762 | -0.135 | 0.332 | 0.160 | 0.251 | 0.105 | 0.454 | 0.134 | 0.333 | 0.154 | 0.265 | 0.152 | 0.272 | 0.112 | 0.423 |
| Class-switched memory B-cells | 0.102 | 0.464 | -0.382 | 0.004 | 0.168 | 0.229 | 0.072 | 0.611 | 0.184 | 0.184 | 0.236 | 0.086 | 0.125 | 0.369 | 0.158 | 0.259 |
| Monocytes | 0.283 | 0.038 | -0.019 | 0.893 | 0.123 | 0.382 | 0.211 | 0.128 | 0.226 | 0.100 | 0.316 | 0.020 | 0.235 | 0.087 | 0.218 | 0.116 |
| Classical monocytes | 0.258 | 0.060 | -0.075 | 0.589 | 0.171 | 0.222 | 0.274 | 0.047 | 0.202 | 0.143 | 0.225 | 0.101 | 0.126 | 0.362 | 0.200 | 0.152 |
| Intermediate monocytes | -0.265 | 0.053 | -0.021 | 0.880 | -0.227 | 0.103 | -0.330 | 0.016 | -0.276 | 0.043 | -0.241 | 0.079 | -0.217 | 0.116 | -0.234 | 0.092 |
| Non-classical monocytes | -0.215 | 0.119 | 0.185 | 0.182 | -0.114 | 0.415 | -0.178 | 0.201 | -0.125 | 0.367 | -0.196 | 0.157 | -0.063 | 0.650 | -0.137 | 0.328 |
| Total pDC | 0.085 | 0.543 | -0.160 | 0.248 | 0.080 | 0.570 | 0.000 | 0.999 | 0.054 | 0.699 | 0.114 | 0.411 | 0.062 | 0.654 | 0.045 | 0.748 |
| Total mDC | -0.015 | 0.916 | -0.139 | 0.315 | 0.028 | 0.840 | -0.138 | 0.325 | 0.006 | 0.966 | 0.071 | 0.608 | -0.040 | 0.773 | 0.041 | 0.773 |
| Total HSC | -0.119 | 0.391 | 0.150 | 0.280 | -0.117 | 0.404 | -0.074 | 0.600 | -0.061 | 0.659 | -0.013 | 0.925 | -0.024 | 0.866 | -0.014 | 0.923 |
| CD4^+^CD27^+^ | -0.074 | 0.595 | 0.013 | 0.928 | -0.002 | 0.987 | 0.040 | 0.774 | -0.059 | 0.672 | -0.107 | 0.441 | -0.010 | 0.943 | -0.096 | 0.495 |
| CD4^+^CD28^+^ | -0.241 | 0.080 | 0.139 | 0.316 | -0.170 | 0.224 | -0.185 | 0.184 | -0.239 | 0.082 | -0.263 | 0.055 | -0.194 | 0.160 | -0.303 | 0.028 |
| CD4^+^CD27^+^CD28^+^ | -0.093 | 0.505 | 0.020 | 0.884 | -0.011 | 0.940 | 0.005 | 0.974 | -0.091 | 0.515 | -0.133 | 0.336 | -0.028 | 0.841 | -0.120 | 0.393 |
| CD4^+^CD27^-^CD28^-^ | 0.206 | 0.136 | -0.128 | 0.355 | 0.145 | 0.302 | 0.153 | 0.275 | 0.209 | 0.129 | 0.234 | 0.089 | 0.164 | 0.237 | 0.287 | 0.037 |
| CD4^+^CD57^+^ | 0.182 | 0.189 | -0.086 | 0.534 | 0.064 | 0.650 | 0.078 | 0.580 | 0.129 | 0.352 | 0.199 | 0.150 | 0.115 | 0.406 | 0.168 | 0.229 |
| CD4^+^ Tregs | -0.171 | 0.216 | -0.107 | 0.441 | -0.208 | 0.136 | -0.115 | 0.413 | -0.134 | 0.336 | -0.123 | 0.374 | -0.112 | 0.419 | -0.192 | 0.170 |
| Naive CD4^+^ | -0.039 | 0.781 | -0.014 | 0.923 | 0.028 | 0.842 | 0.057 | 0.684 | -0.024 | 0.865 | -0.118 | 0.397 | 0.077 | 0.579 | -0.042 | 0.763 |
| Naive CD4^+^CD27^+^ | -0.036 | 0.796 | -0.010 | 0.942 | 0.036 | 0.800 | 0.060 | 0.668 | -0.014 | 0.917 | -0.111 | 0.425 | 0.083 | 0.553 | -0.032 | 0.822 |
| Naive CD4^+^CD28^+^ | -0.049 | 0.723 | 0.005 | 0.969 | 0.031 | 0.825 | 0.054 | 0.701 | -0.029 | 0.835 | -0.124 | 0.372 | 0.074 | 0.594 | -0.053 | 0.707 |
| Naive CD4^+^CD27^+^CD28^+^ | -0.044 | 0.749 | -0.011 | 0.935 | 0.040 | 0.775 | 0.041 | 0.769 | -0.037 | 0.789 | -0.127 | 0.360 | 0.072 | 0.605 | -0.049 | 0.728 |
| Naive CD4^+^CD27^-^CD28^-^ | 0.123 | 0.374 | 0.071 | 0.612 | -0.012 | 0.930 | 0.121 | 0.389 | 0.070 | 0.615 | 0.078 | 0.577 | 0.051 | 0.716 | 0.153 | 0.274 |
| Naive CD4^+^CD57^+^ | 0.050 | 0.718 | 0.087 | 0.530 | -0.113 | 0.420 | 0.028 | 0.840 | 0.007 | 0.963 | 0.057 | 0.682 | 0.019 | 0.890 | 0.012 | 0.934 |
| CM CD4^+^ | -0.042 | 0.762 | -0.119 | 0.390 | -0.189 | 0.176 | -0.155 | 0.268 | -0.147 | 0.287 | -0.104 | 0.453 | -0.167 | 0.227 | -0.175 | 0.209 |
| CM CD4^+^CD27^+^ | -0.013 | 0.924 | -0.104 | 0.454 | -0.132 | 0.345 | -0.113 | 0.421 | -0.104 | 0.452 | -0.070 | 0.617 | -0.119 | 0.390 | -0.131 | 0.348 |
| CM CD4^+^CD28^+^ | -0.049 | 0.725 | -0.120 | 0.386 | -0.192 | 0.169 | -0.160 | 0.251 | -0.154 | 0.265 | -0.112 | 0.419 | -0.170 | 0.219 | -0.181 | 0.195 |
| CM CD4^+^CD27^+^CD28^+^ | -0.011 | 0.938 | -0.106 | 0.444 | -0.127 | 0.365 | -0.110 | 0.434 | -0.099 | 0.475 | -0.066 | 0.636 | -0.117 | 0.400 | -0.126 | 0.368 |
| CM CD4^+^CD27^-^CD28^-^ | 0.103 | 0.458 | -0.089 | 0.522 | -0.010 | 0.942 | 0.059 | 0.677 | 0.092 | 0.507 | 0.111 | 0.426 | 0.090 | 0.519 | 0.120 | 0.394 |
| CM CD4^+^CD57^+^ | 0.043 | 0.757 | -0.059 | 0.670 | -0.156 | 0.263 | -0.089 | 0.526 | -0.077 | 0.582 | 0.016 | 0.909 | -0.073 | 0.601 | -0.063 | 0.652 |
| EM CD4^+^ | 0.070 | 0.613 | 0.102 | 0.462 | 0.074 | 0.599 | 0.017 | 0.904 | 0.063 | 0.648 | 0.115 | 0.408 | 0.075 | 0.592 | 0.069 | 0.625 |
| EM CD4^+^CD27^+^ | 0.043 | 0.759 | 0.192 | 0.165 | 0.173 | 0.214 | 0.124 | 0.375 | 0.114 | 0.412 | 0.125 | 0.367 | 0.117 | 0.401 | 0.105 | 0.453 |
| EM CD4^+^CD28^+^ | 0.021 | 0.883 | 0.162 | 0.243 | 0.112 | 0.423 | 0.034 | 0.808 | 0.065 | 0.640 | 0.092 | 0.509 | 0.076 | 0.587 | 0.046 | 0.741 |
| EM CD4^+^CD27^+^CD28^+^ | 0.048 | 0.730 | 0.201 | 0.144 | 0.183 | 0.190 | 0.131 | 0.349 | 0.121 | 0.384 | 0.128 | 0.355 | 0.121 | 0.385 | 0.112 | 0.424 |
| EM CD4^+^CD27^-^CD28^-^ | 0.274 | 0.045 | -0.105 | 0.450 | 0.184 | 0.187 | 0.233 | 0.094 | 0.258 | 0.059 | 0.291 | 0.033 | 0.250 | 0.069 | 0.310 | 0.024 |
| EM CD4^+^CD57^+^ | 0.223 | 0.105 | -0.070 | 0.616 | 0.106 | 0.449 | 0.100 | 0.478 | 0.159 | 0.252 | 0.221 | 0.108 | 0.167 | 0.227 | 0.178 | 0.204 |
| TEMRA CD4^+^ | 0.060 | 0.664 | 0.164 | 0.236 | 0.128 | 0.361 | 0.083 | 0.553 | 0.138 | 0.319 | 0.091 | 0.513 | 0.039 | 0.782 | 0.196 | 0.159 |
| TEMRA CD4^+^CD27^+^ | -0.003 | 0.985 | 0.355 | 0.009 | 0.234 | 0.091 | 0.152 | 0.277 | 0.172 | 0.214 | 0.124 | 0.374 | 0.136 | 0.327 | 0.153 | 0.273 |
| TEMRA CD4^+^CD28^+^ | -0.054 | 0.700 | 0.261 | 0.057 | 0.182 | 0.193 | 0.094 | 0.502 | 0.134 | 0.335 | 0.078 | 0.574 | 0.124 | 0.372 | 0.100 | 0.477 |
| TEMRA CD4^+^CD27^+^CD28^+^ | -0.023 | 0.868 | 0.323 | 0.017 | 0.226 | 0.104 | 0.146 | 0.298 | 0.168 | 0.224 | 0.116 | 0.402 | 0.126 | 0.364 | 0.129 | 0.358 |
| TEMRA CD4^+^CD27^-^CD28^-^ | 0.260 | 0.058 | -0.132 | 0.340 | 0.227 | 0.102 | 0.202 | 0.148 | 0.257 | 0.061 | 0.249 | 0.069 | 0.198 | 0.152 | 0.368 | 0.007 |
| TEMRA CD4^+^CD57^+^ | 0.230 | 0.094 | -0.087 | 0.533 | 0.213 | 0.126 | 0.161 | 0.248 | 0.230 | 0.094 | 0.237 | 0.084 | 0.176 | 0.204 | 0.325 | 0.017 |
| CD8^+^CD27^+^ | -0.125 | 0.368 | 0.057 | 0.683 | -0.078 | 0.578 | -0.117 | 0.403 | -0.098 | 0.480 | -0.119 | 0.393 | -0.102 | 0.461 | -0.234 | 0.092 |
| CD8^+^CD28^+^ | -0.219 | 0.111 | -0.016 | 0.911 | -0.152 | 0.277 | -0.168 | 0.230 | -0.183 | 0.185 | -0.225 | 0.101 | -0.198 | 0.152 | -0.306 | 0.026 |
| CD8^+^CD27^+^CD28^+^ | -0.134 | 0.334 | 0.056 | 0.687 | -0.069 | 0.622 | -0.116 | 0.407 | -0.089 | 0.520 | -0.133 | 0.338 | -0.085 | 0.542 | -0.222 | 0.110 |
| CD8^+^CD27^-^CD28^-^ | 0.197 | 0.154 | -0.010 | 0.944 | 0.128 | 0.361 | 0.159 | 0.257 | 0.167 | 0.228 | 0.198 | 0.152 | 0.185 | 0.181 | 0.292 | 0.034 |
| CD8^+^CD57^+^ | 0.190 | 0.170 | 0.071 | 0.609 | 0.111 | 0.430 | 0.142 | 0.312 | 0.135 | 0.331 | 0.198 | 0.151 | 0.155 | 0.264 | 0.222 | 0.110 |
| Naive CD8^+^ | -0.041 | 0.770 | 0.033 | 0.810 | -0.034 | 0.810 | -0.032 | 0.818 | -0.034 | 0.807 | -0.086 | 0.536 | -0.071 | 0.608 | -0.123 | 0.379 |
| Naive CD8^+^CD27^+^ | -0.048 | 0.731 | 0.027 | 0.845 | -0.003 | 0.985 | -0.021 | 0.880 | -0.012 | 0.930 | -0.072 | 0.605 | -0.048 | 0.728 | -0.129 | 0.358 |
| Naive CD8^+^CD28^+^ | -0.065 | 0.642 | 0.027 | 0.844 | -0.024 | 0.867 | -0.034 | 0.808 | -0.031 | 0.824 | -0.084 | 0.545 | -0.066 | 0.638 | -0.140 | 0.316 |
| Naive CD8^+^CD27^+^CD28^+^ | -0.051 | 0.713 | 0.043 | 0.758 | -0.003 | 0.983 | -0.022 | 0.874 | -0.016 | 0.909 | -0.072 | 0.604 | -0.050 | 0.718 | -0.133 | 0.344 |
| Naive CD8^+^CD27^-^CD28^-^ | 0.128 | 0.355 | 0.063 | 0.650 | -0.031 | 0.826 | 0.090 | 0.524 | 0.071 | 0.609 | 0.047 | 0.734 | 0.024 | 0.863 | 0.149 | 0.286 |
| Naive CD8^+^CD57^+^ | 0.085 | 0.541 | 0.065 | 0.642 | -0.067 | 0.633 | 0.066 | 0.639 | 0.041 | 0.769 | 0.055 | 0.695 | 0.023 | 0.871 | 0.049 | 0.726 |
| CM CD8^+^ | -0.203 | 0.141 | 0.020 | 0.884 | -0.264 | 0.056 | -0.237 | 0.087 | -0.295 | 0.030 | -0.267 | 0.051 | -0.292 | 0.032 | -0.289 | 0.036 |
| CM CD8^+^CD27^+^ | -0.199 | 0.149 | 0.049 | 0.725 | -0.213 | 0.126 | -0.192 | 0.169 | -0.224 | 0.103 | -0.207 | 0.133 | -0.219 | 0.111 | -0.267 | 0.053 |
| CM CD8^+^CD28^+^ | -0.198 | 0.152 | 0.005 | 0.973 | -0.230 | 0.097 | -0.202 | 0.147 | -0.261 | 0.057 | -0.247 | 0.072 | -0.261 | 0.056 | -0.287 | 0.037 |
| CM CD8^+^CD27^+^CD28^+^ | -0.208 | 0.131 | 0.035 | 0.801 | -0.217 | 0.118 | -0.199 | 0.153 | -0.225 | 0.102 | -0.209 | 0.129 | -0.221 | 0.108 | -0.275 | 0.046 |
| CM CD8^+^CD27^-^CD28^-^ | -0.013 | 0.926 | -0.011 | 0.936 | -0.151 | 0.280 | -0.139 | 0.321 | -0.105 | 0.448 | -0.030 | 0.831 | -0.099 | 0.477 | -0.033 | 0.815 |
| CM CD8^+^CD57^+^ | -0.057 | 0.681 | 0.105 | 0.450 | -0.161 | 0.249 | -0.120 | 0.394 | -0.127 | 0.360 | -0.082 | 0.554 | -0.084 | 0.546 | -0.112 | 0.425 |
| EM CD8^+^ | -0.130 | 0.348 | -0.105 | 0.449 | -0.145 | 0.300 | -0.199 | 0.153 | -0.224 | 0.104 | -0.129 | 0.352 | -0.196 | 0.155 | -0.190 | 0.174 |
| EM CD8^+^CD27^+^ | -0.109 | 0.433 | -0.015 | 0.916 | -0.061 | 0.664 | -0.099 | 0.482 | -0.092 | 0.507 | -0.042 | 0.761 | -0.038 | 0.783 | -0.204 | 0.143 |
| EM CD8^+^CD28^+^ | -0.191 | 0.166 | -0.102 | 0.463 | -0.088 | 0.531 | -0.143 | 0.306 | -0.162 | 0.242 | -0.140 | 0.311 | -0.166 | 0.230 | -0.205 | 0.141 |
| EM CD8^+^CD27^+^CD28^+^ | -0.143 | 0.303 | -0.052 | 0.710 | -0.065 | 0.642 | -0.097 | 0.488 | -0.108 | 0.437 | -0.072 | 0.606 | -0.064 | 0.644 | -0.203 | 0.145 |
| EM CD8^+^CD27^-^CD28^-^ | 0.088 | 0.527 | -0.130 | 0.350 | 0.038 | 0.789 | 0.068 | 0.630 | 0.040 | 0.776 | 0.136 | 0.328 | 0.068 | 0.628 | 0.149 | 0.288 |
| EM CD8^+^CD57^+^ | 0.054 | 0.700 | -0.013 | 0.928 | -0.011 | 0.939 | -0.019 | 0.894 | -0.027 | 0.847 | 0.083 | 0.550 | 0.019 | 0.889 | 0.014 | 0.920 |
| TEMRA CD8^+^ | 0.196 | 0.156 | 0.100 | 0.470 | 0.269 | 0.052 | 0.294 | 0.033 | 0.301 | 0.027 | 0.238 | 0.083 | 0.257 | 0.061 | 0.355 | 0.009 |
| TEMRA CD8^+^CD27^+^ | 0.163 | 0.240 | 0.234 | 0.089 | 0.345 | 0.011 | 0.302 | 0.028 | 0.330 | 0.015 | 0.269 | 0.049 | 0.292 | 0.032 | 0.193 | 0.166 |
| TEMRA CD8^+^CD28^+^ | -0.049 | 0.723 | 0.067 | 0.632 | 0.164 | 0.241 | 0.145 | 0.300 | 0.139 | 0.316 | 0.045 | 0.748 | 0.129 | 0.354 | 0.050 | 0.721 |
| TEMRA CD8^+^CD27^+^CD28^+^ | 0.130 | 0.349 | 0.175 | 0.206 | 0.310 | 0.024 | 0.274 | 0.048 | 0.289 | 0.034 | 0.212 | 0.124 | 0.265 | 0.053 | 0.159 | 0.254 |
| TEMRA CD8^+^CD27^-^CD28^-^ | 0.215 | 0.119 | 0.027 | 0.844 | 0.212 | 0.127 | 0.222 | 0.111 | 0.228 | 0.097 | 0.193 | 0.162 | 0.214 | 0.121 | 0.345 | 0.011 |
| TEMRA CD8^+^CD57^+^ | 0.232 | 0.091 | 0.080 | 0.567 | 0.232 | 0.095 | 0.248 | 0.074 | 0.249 | 0.069 | 0.218 | 0.114 | 0.241 | 0.079 | 0.360 | 0.008 |
| ***T-cell p16^INK4a^ expression*** | | | | | | | | | | | | | | | | |
| *p16^INK4a^* | 0.285 | 0.068 | 0.066 | 0.676 | 0.197 | 0.217 | 0.260 | 0.096 | 0.274 | 0.08 | 0.135 | 0.394 | 0.258 | 0.099 | 0.311 | 0.048 |
| ***Plasma circulating miRs*** | | | | | | | | | | | | | | | | |
| let-7e | -0.098 | 0.449 | -0.219 | 0.088 | -0.183 | 0.159 | -0.225 | 0.081 | -0.152 | 0.240 | -0.119 | 0.359 | -0.185 | 0.151 | -0.147 | 0.258 |
| let-7i | -0.117 | 0.367 | -0.053 | 0.684 | -0.152 | 0.241 | -0.177 | 0.173 | -0.123 | 0.343 | -0.130 | 0.314 | -0.151 | 0.242 | -0.186 | 0.150 |
| miR-9 | 0.087 | 0.501 | -0.262 | 0.039 | 0.153 | 0.239 | 0.102 | 0.434 | 0.139 | 0.282 | 0.073 | 0.574 | 0.170 | 0.186 | 0.105 | 0.422 |
| miR-17 | -0.026 | 0.840 | -0.023 | 0.860 | -0.027 | 0.834 | -0.037 | 0.777 | 0.028 | 0.832 | 0.055 | 0.672 | -0.082 | 0.529 | -0.045 | 0.731 |
| miR-18a | 0.045 | 0.731 | -0.110 | 0.393 | 0.031 | 0.814 | -0.007 | 0.960 | 0.092 | 0.479 | 0.155 | 0.228 | 0.038 | 0.767 | -0.079 | 0.546 |
| miR-19a | -0.047 | 0.717 | -0.234 | 0.067 | -0.023 | 0.862 | -0.032 | 0.809 | -0.034 | 0.796 | -0.050 | 0.701 | -0.026 | 0.840 | -0.090 | 0.492 |
| miR-19b | 0.032 | 0.806 | -0.171 | 0.183 | 0.154 | 0.237 | 0.103 | 0.427 | 0.112 | 0.385 | 0.098 | 0.450 | 0.139 | 0.280 | 0.011 | 0.931 |
| miR-20a | 0.078 | 0.548 | 0.130 | 0.314 | 0.200 | 0.123 | 0.220 | 0.088 | 0.217 | 0.090 | 0.213 | 0.096 | 0.208 | 0.105 | 0.157 | 0.226 |
| miR-21 | -0.026 | 0.840 | 0.028 | 0.831 | 0.021 | 0.871 | -0.005 | 0.969 | 0.077 | 0.554 | 0.045 | 0.728 | 0.079 | 0.544 | 0.000 | 1.000 |
| miR-92a | -0.142 | 0.273 | 0.155 | 0.229 | -0.079 | 0.543 | -0.127 | 0.330 | -0.101 | 0.433 | -0.102 | 0.428 | -0.107 | 0.406 | -0.048 | 0.712 |
| miR-125b | 0.084 | 0.515 | -0.047 | 0.718 | 0.254 | 0.049 | 0.200 | 0.122 | 0.174 | 0.176 | 0.184 | 0.152 | 0.251 | 0.049 | 0.160 | 0.217 |
| miR-126 | 0.146 | 0.257 | 0.055 | 0.673 | 0.173 | 0.183 | 0.129 | 0.320 | 0.227 | 0.076 | 0.224 | 0.080 | 0.146 | 0.258 | 0.214 | 0.097 |
| miR-146a | 0.047 | 0.718 | 0.025 | 0.849 | 0.027 | 0.837 | -0.016 | 0.900 | 0.094 | 0.465 | 0.130 | 0.314 | -0.014 | 0.916 | 0.023 | 0.858 |
| miR-150 | 0.107 | 0.406 | 0.017 | 0.898 | 0.160 | 0.218 | 0.176 | 0.176 | 0.128 | 0.321 | 0.131 | 0.310 | 0.258 | 0.043 | 0.189 | 0.144 |
| miR-155 | 0.047 | 0.718 | -0.026 | 0.840 | -0.064 | 0.622 | 0.038 | 0.772 | 0.002 | 0.987 | -0.031 | 0.813 | -0.003 | 0.981 | 0.070 | 0.590 |
| miR-181a | -0.183 | 0.153 | -0.128 | 0.321 | -0.183 | 0.158 | -0.090 | 0.489 | -0.086 | 0.507 | -0.114 | 0.379 | -0.084 | 0.517 | -0.167 | 0.200 |
| miR-195 | 0.192 | 0.134 | 0.211 | 0.099 | 0.313 | 0.014 | 0.319 | 0.012 | 0.264 | 0.038 | 0.320 | 0.011 | 0.171 | 0.184 | 0.299 | 0.019 |
| miR-223 | 0.094 | 0.469 | -0.056 | 0.668 | 0.034 | 0.796 | 0.052 | 0.691 | 0.120 | 0.351 | 0.136 | 0.293 | 0.005 | 0.968 | 0.071 | 0.586 |
| miR-326 | 0.014 | 0.915 | 0.000 | 0.998 | -0.075 | 0.566 | -0.006 | 0.961 | -0.060 | 0.641 | -0.112 | 0.386 | -0.077 | 0.552 | 0.000 | 0.997 |
| miR-424 | -0.092 | 0.476 | -0.253 | 0.047 | -0.206 | 0.112 | -0.149 | 0.251 | -0.159 | 0.216 | -0.210 | 0.102 | -0.235 | 0.066 | -0.242 | 0.061 |
