# Supplementary material for: Age‐related remodelling of the blood immunological portrait and the local tumor immune response in patients with luminal breast cancer
Source: Clin Transl Immunology. 2020 Oct 3;9(10):e1184. doi: 10.1002/cti2.1184 (PMC7532981; doi:10.1002/cti2.1184)
Supplement: Supplementary file 8 [file CTI2-9-e1184-s008.docx]

*Supplementary table 7 - Correlations between the blood immune/senescence (plasma protein biomarkers, PBMC subset profiling, T-cell p16^INK4a^ expression and plasma circulating miRs), tumor immune infiltrate (sTILs %, CD68 staining grade, density of CD3^+^, CD4^+^, CD5^+^, CD8^+^, CD20^+^ and FOXP3^+^ cells) and pathological tumor characteristics (tumor size, tumor grade and lymph node involvement) are shown. Rho and the P-values are reported for tumor size, the P-values were obtained by using the Spearman correlations test, significance threshold was set below 5% (marked in grey). The color scale indicates the direction and strength of the correlation. Values marked in green indicate a negative correlation; values marked in red indicate a positive correlation. For tumor grade and lymph node involvement (pN^-^: no lymph node involvement, pN^+^: lymph node involvement) the median and P-values are reported and these were calculated by using the Mann-Whitney U test, significance threshold was set below 5% (marked in grey).*

*
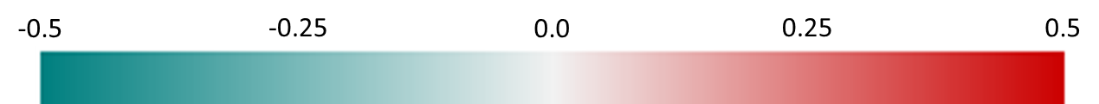
*

|  | **Tumor size** | | **Tumor grade** | | | **Lymph node involvement** | | |  |
| --- | --- | --- | --- | --- | --- | --- | --- | --- | --- |
|  | Rho | *P*-value | Median | | *P*-value | Median | | *P*-value |  |
|  |  |  | Grade I-II | Grade III |  | pN^-^ | pN^+^ |  |  |
| ***Plasma protein biomarkers*** | | | | | | | | | |
| IL-1α | 0.092 | 0.466 | 10.4 | 9.7 | 0.462 | 9.7 | 10.4 | 0.984 |  |
| IL-17A | 0.052 | 0.681 | 0.7 | 0.5 | 0.505 | 0.6 | 0.7 | 0.335 |  |
| IL-1β | 0.225 | 0.072 | 15 | 13 | 0.292 | 13.3 | 15.6 | 0.632 |  |
| IL-6 | 0.230 | 0.065 | 8.8 | 8.2 | 0.649 | 9.5 | 7.7 | 0.348 |  |
| IL-10 | 0.111 | 0.380 | 7.3 | 5.9 | 0.514 | 6.9 | 6.5 | 0.699 |  |
| IL-12p70 | 0.122 | 0.332 | 7.9 | 5.6 | 0.305 | 6.1 | 5.7 | 0.572 |  |
| IL-17F | -0.155 | 0.219 | 0 | 0 | 0.871 | 0 | 0 | 0.164 |  |
| IL-27 | 0.175 | 0.164 | 7.4 | 5.7 | 0.331 | 7.4 | 6.4 | 0.901 |  |
| IFNγ | 0.135 | 0.286 | 9.2 | 8.1 | 0.434 | 8.8 | 9.5 | 0.541 |  |
| TNFα | 0.105 | 0.406 | 3.2 | 2.2 | 0.358 | 3.3 | 2.5 | 0.718 |  |
| IP-10 | 0.159 | 0.208 | 61 | 63.1 | 0.854 | 58.7 | 63 | 0.906 |  |
| IL-8 | 0.153 | 0.224 | 21.2 | 20.7 | 0.729 | 23.1 | 19.2 | 0.142 |  |
| MCP-1 | 0.160 | 0.204 | 132.2 | 112.8 | 0.133 | 131 | 120.9 | 0.332 |  |
| Gal-9 | 0.200 | 0.111 | 47 | 56.3 | 0.729 | 52.3 | 47 | 0.738 |  |
| sCD25 | 0.093 | 0.462 | 707.5 | 609.5 | 0.419 | 685.2 | 699.2 | 0.829 |  |
| TIM-3 | 0.137 | 0.277 | 7.1 | 6.5 | 0.518 | 6.8 | 6.8 | 0.568 |  |
| 4-1BB | -0.059 | 0.645 | 0 | 0 | 0.771 | 0 | 0 | 0.340 |  |
| PD-L1 | -0.104 | 0.413 | 1.3 | 0.9 | 0.204 | 1.1 | 1.3 | 0.303 |  |
| sCD27 | 0.073 | 0.565 | 41.8 | 34.2 | 0.140 | 42 | 34.3 | 0.458 |  |
| CD86 | -0.039 | 0.760 | 119.5 | 134.9 | 0.908 | 117.1 | 134.3 | 0.111 |  |
| CTLA-4 | -0.097 | 0.442 | 0 | 0 | 0.626 | 0 | 0 | 0.892 |  |
| Free active TGF-β1 | -0.067 | 0.599 | 0 | 0 | 0.286 | 0 | 0 | 0.167 |  |
| LAG-3 | 0.130 | 0.302 | 431.3 | 516 | 0.887 | 447.3 | 491.6 | 0.880 |  |
| PD-1 | 0.009 | 0.945 | 0.4 | 0.3 | 0.089 | 0.3 | 0.5 | 0.256 |  |
| PD-L2 | 0.008 | 0.947 | 9.1 | 7.8 | 0.083 | 8.9 | 8.5 | 0.550 |  |
| CRP | -0.028 | 0.825 | 849.8 | 678.3 | 0.286 | 841.2 | 756 | 0.508 |  |
| IGF-1 | -0.218 | 0.081 | 87.6 | 91.8 | 0.669 | 89.5 | 92.8 | 0.397 |  |
| ***PBMC subset profiling (%)*** | | | | | | | | | |
| CD3^+^ Lymphocytes | 0.262 | 0.049 | 48.1 | 52.1 | 0.134 | 47.1 | 49.8 | 0.350 |  |
| CD4^+^ Lymphocytes | 0.183 | 0.175 | 26.3 | 25.3 | 0.823 | 25.3 | 28 | 0.448 |  |
| CD8^+^ Lymphocytes | -0.030 | 0.827 | 14 | 19.4 | 0.034 | 16.9 | 14.8 | 0.780 |  |
| CD4/CD8 ratio | 0.076 | 0.576 | 1.8 | 1.1 | 0.224 | 1.6 | 1.8 | 0.638 |  |
| Total Treg cells | 0.068 | 0.615 | 1 | 1.1 | 0.980 | 1.1 | 1.1 | 0.955 |  |
| Naive Treg cells | -0.058 | 0.671 | 69.5 | 62.3 | 0.063 | 69.2 | 64.2 | 0.604 |  |
| Memory Treg cells | 0.093 | 0.494 | 30.5 | 35.3 | 0.286 | 30.8 | 33.3 | 0.981 |  |
| CD3^+^CD16^+^ cells | -0.181 | 0.179 | 2.3 | 4.6 | 0.025 | 3.1 | 2.1 | 0.153 |  |
| Total NK cells | -0.196 | 0.144 | 8.4 | 8.2 | 0.697 | 9.5 | 6.7 | 0.120 |  |
| CD56^bright^CD16^-^ NK cells | -0.088 | 0.516 | 6.5 | 4.9 | 0.250 | 4.9 | 7.7 | 0.080 |  |
| CD56^dim^CD16^+^ NK Cells | 0.124 | 0.359 | 87.5 | 91.1 | 0.271 | 91.8 | 86.6 | 0.120 |  |
| B-cells | -0.097 | 0.477 | 12.1 | 6.6 | 0.090 | 9 | 11.7 | 0.930 |  |
| Naive B-cells | -0.108 | 0.424 | 44.6 | 42.4 | 0.673 | 49.6 | 40 | 0.311 |  |
| Non-switched memory B-cells | 0.102 | 0.453 | 21.6 | 20.6 | 0.849 | 19.9 | 23.9 | 0.318 |  |
| Class-switched memory B-cells | 0.076 | 0.575 | 25.2 | 27.8 | 0.888 | 25.3 | 24.6 | 0.955 |  |
| Monocytes | -0.254 | 0.056 | 14.5 | 10.1 | 0.862 | 15.4 | 11.8 | 0.487 |  |
| Classical monocytes | -0.210 | 0.117 | 84 | 83.9 | 0.747 | 84 | 83.7 | 0.930 |  |
| Intermediate monocytes | 0.219 | 0.102 | 8.6 | 5.8 | 0.194 | 8.8 | 6.6 | 0.560 |  |
| Non-classical monocytes | 0.205 | 0.126 | 8.4 | 8.2 | 0.591 | 8.2 | 8.4 | 0.731 |  |
| Total pDC | 0.011 | 0.933 | 0.2 | 0.2 | 0.798 | 0.2 | 0.2 | 0.334 |  |
| Total mDC | 0.100 | 0.459 | 1 | 1.1 | 0.875 | 1 | 1 | 0.367 |  |
| Total HSC | -0.286 | 0.031 | 0.1 | 0.1 | 0.060 | 0.1 | 0.1 | 0.059 |  |
| CD4^+^CD27^+^ | 0.086 | 0.527 | 72.2 | 69.6 | 0.747 | 69.6 | 74.3 | 0.598 |  |
| CD4^+^CD28^+^ | 0.201 | 0.134 | 97.9 | 94.4 | 0.188 | 96.1 | 96.9 | 0.823 |  |
| CD4^+^CD27^+^CD28^+^ | 0.081 | 0.549 | 71.4 | 64.4 | 0.626 | 68.8 | 71.4 | 0.756 |  |
| CD4^+^CD27^-^CD28^-^ | -0.215 | 0.108 | 1.2 | 4.7 | 0.155 | 3.1 | 2 | 0.544 |  |
| CD4^+^CD57^+^ | -0.149 | 0.270 | 5.9 | 8.5 | 0.579 | 8.5 | 4.8 | 0.439 |  |
| CD4^+^ Tregs | -0.005 | 0.971 | 3.5 | 3.4 | 0.967 | 3.6 | 3.3 | 0.968 |  |
| Naive CD4^+^ | 0.095 | 0.482 | 31.9 | 33.7 | 0.980 | 31.1 | 35.1 | 0.867 |  |
| Naive CD4^+^CD27^+^ | 0.097 | 0.475 | 30.4 | 32.1 | 0.967 | 29.8 | 31.9 | 0.743 |  |
| Naive CD4^+^CD28^+^ | 0.119 | 0.381 | 30.6 | 33.6 | 0.980 | 30.3 | 34.3 | 0.854 |  |
| Naive CD4^+^CD27^+^CD28^+^ | 0.089 | 0.512 | 29.7 | 31.9 | 0.928 | 29.5 | 31.5 | 0.955 |  |
| Naive CD4^+^CD27^-^CD28^-^ | -0.186 | 0.167 | 0.1 | 0.3 | 0.185 | 0.2 | 0.1 | 0.798 |  |
| Naive CD4^+^CD57^+^ | -0.140 | 0.302 | 0.7 | 0.7 | 0.691 | 0.5 | 0.8 | 0.975 |  |
| CM CD4^+^ | -0.032 | 0.814 | 17.4 | 13.4 | 0.043 | 17.3 | 15.7 | 0.458 |  |
| CM CD4^+^CD27^+^ | -0.006 | 0.964 | 14.7 | 11.7 | 0.092 | 14.6 | 13 | 0.678 |  |
| CM CD4^+^CD28^+^ | -0.030 | 0.826 | 17.3 | 13.4 | 0.041 | 17.3 | 15.2 | 0.448 |  |
| CM CD4^+^CD27^+^CD28^+^ | -0.007 | 0.956 | 14.7 | 11.7 | 0.098 | 14.6 | 12.9 | 0.672 |  |
| CM CD4^+^CD27^-^CD28^-^ | -0.225 | 0.092 | 0 | 0 | 0.363 | 0 | 0 | 0.980 |  |
| CM CD4^+^CD57^+^ | -0.172 | 0.202 | 0.2 | 0.1 | 0.297 | 0.2 | 0.2 | 0.482 |  |
| EM CD4^+^ | -0.046 | 0.734 | 28.2 | 29.2 | 0.337 | 30.2 | 27.8 | 0.503 |  |
| EM CD4^+^CD27^+^ | 0.085 | 0.531 | 11.6 | 14.3 | 0.148 | 12.5 | 12.6 | 0.905 |  |
| EM CD4^+^CD28^+^ | 0.005 | 0.973 | 24.1 | 26.8 | 0.579 | 24.8 | 26.1 | 0.672 |  |
| EM CD4^+^CD27^+^CD28^+^ | 0.083 | 0.542 | 11.6 | 14.3 | 0.143 | 12 | 12.5 | 0.892 |  |
| EM CD4^+^CD27^-^CD28^-^ | -0.278 | 0.036 | 0.2 | 1.3 | 0.073 | 0.4 | 0.4 | 0.416 |  |
| EM CD4^+^CD57^+^ | -0.215 | 0.108 | 1.3 | 3.8 | 0.350 | 2.2 | 1.2 | 0.127 |  |
| TEMRA CD4^+^ | -0.095 | 0.485 | 8.6 | 11 | 0.060 | 9.5 | 7.9 | 0.359 |  |
| TEMRA CD4^+^CD27^+^ | 0.060 | 0.658 | 2.9 | 4.9 | 0.036 | 4 | 3.5 | 0.873 |  |
| TEMRA CD4^+^CD28^+^ | 0.013 | 0.922 | 4.6 | 5.6 | 0.107 | 5.6 | 5.2 | 0.867 |  |
| TEMRA CD4^+^CD27^+^CD28^+^ | 0.054 | 0.692 | 2.7 | 4.7 | 0.040 | 3.9 | 3.5 | 0.949 |  |
| TEMRA CD4^+^CD27^-^CD28^-^ | -0.248 | 0.063 | 0.7 | 3.1 | 0.167 | 2.1 | 0.7 | 0.411 |  |
| TEMRA CD4^+^CD57^+^ | -0.203 | 0.130 | 0.9 | 1.5 | 0.442 | 2.3 | 0.9 | 0.330 |  |
| CD8^+^CD27^+^ | -0.109 | 0.422 | 50.8 | 39.3 | 0.200 | 43.3 | 51.2 | 0.539 |  |
| CD8^+^CD28^+^ | 0.145 | 0.283 | 67.9 | 61.8 | 0.286 | 61.8 | 67.4 | 0.311 |  |
| CD8^+^CD27^+^CD28^+^ | -0.125 | 0.355 | 45.1 | 32 | 0.215 | 40.3 | 45.1 | 0.503 |  |
| CD8^+^CD27^-^CD28^-^ | -0.116 | 0.392 | 29.2 | 37.5 | 0.230 | 35.9 | 27.9 | 0.247 |  |
| CD8^+^CD57^+^ | -0.217 | 0.106 | 30.9 | 36.6 | 0.286 | 39.7 | 28.9 | 0.172 |  |
| Naive CD8^+^ | -0.153 | 0.257 | 16.3 | 10.8 | 0.230 | 12.4 | 16.3 | 0.719 |  |
| Naive CD8^+^CD27^+^ | -0.127 | 0.348 | 13.2 | 7.3 | 0.264 | 9.5 | 13.4 | 0.666 |  |
| Naive CD8^+^CD28^+^ | -0.127 | 0.350 | 15.5 | 8.9 | 0.230 | 9.8 | 15.5 | 0.643 |  |
| Naive CD8^+^CD27^+^CD28^+^ | -0.124 | 0.360 | 13 | 7 | 0.257 | 8.8 | 13 | 0.661 |  |
| Naive CD8^+^CD27^-^CD28^-^ | -0.286 | 0.031 | 0.6 | 0.8 | 0.447 | 0.7 | 0.7 | 0.873 |  |
| Naive CD8^+^CD57^+^ | -0.237 | 0.075 | 1.4 | 1.1 | 1.000 | 1.1 | 1.5 | 0.655 |  |
| CM CD8^+^ | 0.074 | 0.585 | 8.3 | 5.4 | 0.021 | 6.6 | 5.9 | 0.655 |  |
| CM CD8^+^CD27^+^ | 0.073 | 0.590 | 5.5 | 3.7 | 0.045 | 4.7 | 4.6 | 0.823 |  |
| CM CD8^+^CD28^+^ | 0.067 | 0.620 | 7.1 | 5.1 | 0.030 | 6.3 | 5.4 | 0.582 |  |
| CM CD8^+^CD27^+^CD28^+^ | 0.066 | 0.625 | 5.4 | 3.7 | 0.041 | 4.4 | 4.3 | 0.792 |  |
| CM CD8^+^CD27^-^CD28^-^ | -0.221 | 0.098 | 0.2 | 0.1 | 0.317 | 0.2 | 0.2 | 0.354 |  |
| CM CD8^+^CD57^+^ | -0.141 | 0.296 | 0.6 | 0.2 | 0.278 | 0.5 | 0.6 | 0.518 |  |
| EM CD8^+^ | 0.204 | 0.128 | 32.7 | 25.8 | 0.747 | 33.2 | 27.7 | 0.684 |  |
| EM CD8^+^CD27^+^ | 0.046 | 0.738 | 12 | 12 | 0.540 | 12 | 11.5 | 0.911 |  |
| EM CD8^+^CD28^+^ | 0.279 | 0.035 | 21 | 19.9 | 0.862 | 20.5 | 20.3 | 0.930 |  |
| EM CD8^+^CD27^+^CD28^+^ | 0.082 | 0.546 | 10.7 | 11 | 0.747 | 11 | 10.3 | 0.780 |  |
| EM CD8^+^CD27^-^CD28^-^ | -0.072 | 0.594 | 4.1 | 4.4 | 0.503 | 4 | 4.3 | 0.621 |  |
| EM CD8^+^CD57^+^ | -0.173 | 0.200 | 6.9 | 5.7 | 0.914 | 6.1 | 7 | 0.892 |  |
| TEMRA CD8^+^ | -0.120 | 0.376 | 33.8 | 40.4 | 0.034 | 36.5 | 39.7 | 0.708 |  |
| TEMRA CD8^+^CD27^+^ | -0.117 | 0.389 | 5.9 | 6.8 | 0.186 | 5.8 | 7.1 | 0.334 |  |
| TEMRA CD8^+^CD28^+^ | 0.123 | 0.362 | 9.1 | 11.3 | 0.206 | 8.8 | 10.3 | 0.207 |  |
| TEMRA CD8^+^CD27^+^CD28^+^ | -0.056 | 0.681 | 4 | 5 | 0.155 | 4.1 | 4.7 | 0.311 |  |
| TEMRA CD8^+^CD27^-^CD28^-^ | -0.119 | 0.381 | 21.8 | 28.7 | 0.101 | 28.4 | 18.7 | 0.162 |  |
| TEMRA CD8^+^CD57^+^ | -0.209 | 0.120 | 20.4 | 28 | 0.090 | 24 | 17.9 | 0.163 |  |
| ***T-cell p16^INK4a^ expression (CNRQ)*** | | | | | | | | | |
| *p16^INK4a^* | -0.043 | 0.784 | 0.5 | 1.1 | 0.037 | 0.7 | 0.6 | 0.635 |  |
| ***Plasma circulating miRs (CNRQ)*** | | | | | | | | | |
| let-7e CNRQ | -0.052 | 0.684 | 1 | 1.1 | 0.492 | 1 | 1.1 | 0.525 |  |
| let-7i CNRQ | -0.149 | 0.239 | 1.1 | 0.9 | 0.064 | 1.1 | 0.9 | 0.055 |  |
| miR-9 CNRQ | 0.033 | 0.795 | 0 | 0 | 0.437 | 0 | 0 | 0.838 |  |
| miR-17 CNRQ | -0.042 | 0.738 | 1.2 | 1 | 0.224 | 1.2 | 1.1 | 0.210 |  |
| miR-18a CNRQ | -0.025 | 0.845 | 1 | 0.9 | 0.919 | 0.9 | 1 | 0.412 |  |
| miR-19a CNRQ | 0.201 | 0.109 | 1.1 | 1 | 0.760 | 1 | 1.1 | 0.718 |  |
| miR-19b CNRQ | 0.150 | 0.235 | 1 | 1 | 0.865 | 1 | 1 | 0.798 |  |
| miR-20a CNRQ | -0.051 | 0.689 | 1 | 1 | 0.366 | 1 | 1 | 0.595 |  |
| miR-21 CNRQ | -0.054 | 0.671 | 0.9 | 1.1 | 0.116 | 1 | 1 | 0.788 |  |
| miR-92a CNRQ | -0.040 | 0.750 | 1.1 | 0.9 | 0.844 | 1 | 1 | 0.516 |  |
| miR-125b CNRQ | -0.006 | 0.959 | 1 | 1.1 | 0.262 | 1 | 1.1 | 0.829 |  |
| miR-126 CNRQ | -0.101 | 0.426 | 0.9 | 1.1 | 0.152 | 0.9 | 1 | 0.911 |  |
| miR-146a CNRQ | -0.023 | 0.858 | 1 | 1.1 | 0.930 | 0.9 | 1.1 | 0.335 |  |
| miR-150 CNRQ | -0.094 | 0.458 | 0.9 | 1.4 | 0.032 | 1.1 | 1 | 0.475 |  |
| miR-155 CNRQ | -0.033 | 0.795 | 0.9 | 1.5 | 0.403 | 1.1 | 1.1 | 0.798 |  |
| miR-181a CNRQ | 0.121 | 0.339 | 0.9 | 0.9 | 0.719 | 0.9 | 0.9 | 0.670 |  |
| miR-195 CNRQ | -0.059 | 0.643 | 0.9 | 1 | 0.229 | 1.1 | 1 | 0.475 |  |
| miR-223 CNRQ | -0.063 | 0.621 | 1 | 1.1 | 0.396 | 1.1 | 1.1 | 0.542 |  |
| miR-326 CNRQ | 0.132 | 0.298 | 0 | 0 | 0.956 | 0 | 0 | 0.612 |  |
| miR-424 CNRQ | 0.158 | 0.209 | 0.9 | 1.4 | 0.582 | 1.5 | 0.9 | 0.718 |  |
| ***Tumor immune infiltrate*** | | | | | | | | | |
| sTILs (%) | -0.246 | 0.054 | 5.4 | 18 | < 0.001 | 7.5 | 7 | 0.612 |  |
| CD68 stainings grade | 0.076 | 0.558 | 2 | 2 | 0.643 | 2 | 2 | 0.305 |  |
| CD3^+^ cells - Tumor center |  |  |  |  |  |  |  |  |  |
| Proportion (%) | 0.411 | 0.001 | 43.3 | 53.2 | 0.008 | 47.9 | 51.4 | 0.432 |  |
| Density (+ cells/mm²) | 0.008 | 0.954 | 79.2 | 380.8 | < 0.001 | 89.3 | 129.5 | 0.713 |  |
| CD3^+^ cells - Invasive front |  |  |  |  |  |  |  |  |  |
| Proportion (%) | 0.316 | 0.013 | 46.4 | 52.4 | 0.068 | 46.2 | 50.9 | 0.352 |  |
| Density (+ cells/mm²) | -0.101 | 0.442 | 163.4 | 850.3 | < 0.001 | 172.4 | 292.7 | 0.670 |  |
| CD3^+^ cells - Whole tumor |  |  |  |  |  |  |  |  |  |
| Proportion (%) | 0.393 | 0.002 | 45 | 53.3 | 0.038 | 45.5 | 51 | 0.316 |  |
| Density (+ cells/mm²) | -0.079 | 0.546 | 126.6 | 624.3 | < 0.001 | 135.8 | 205.9 | 0.423 |  |
| CD4^+^ cells - Tumor center |  |  |  |  |  |  |  |  |  |
| Proportion (%) | 0.057 | 0.662 | 27.7 | 36.6 | 0.002 | 29.6 | 31.9 | 0.440 |  |
| Density (+ cells/mm²) | -0.018 | 0.889 | 42.7 | 205.3 | < 0.001 | 52.7 | 72.7 | 0.789 |  |
| CD4^+^ cells - Invasive front |  |  |  |  |  |  |  |  |  |
| Proportion (%) | 0.086 | 0.513 | 32.7 | 37.9 | 0.013 | 32.8 | 35.7 | 0.245 |  |
| Density (+ cells/mm²) | -0.044 | 0.736 | 146.5 | 705.8 | < 0.001 | 180.5 | 237.1 | 0.649 |  |
| CD4^+^ cells - Whole tumor |  |  |  |  |  |  |  |  |  |
| Proportion (%) | 0.105 | 0.424 | 30.5 | 37 | 0.008 | 31.1 | 34.6 | 0.269 |  |
| Density (+ cells/mm²) | -0.057 | 0.662 | 80.9 | 346 | < 0.001 | 89.6 | 133.3 | 0.374 |  |
| CD5^+^ cells - Tumor center |  |  |  |  |  |  |  |  |  |
| Proportion (%) | 0.070 | 0.590 | 31.1 | 44 | 0.001 | 34.4 | 37.8 | 0.773 |  |
| Density (+ cells/mm²) | -0.098 | 0.449 | 64 | 297.4 | < 0.001 | 75.8 | 96.1 | 0.540 |  |
| CD5^+^ cells - Invasive front |  |  |  |  |  |  |  |  |  |
| Proportion (%) | 0.101 | 0.437 | 37.2 | 50.1 | 0.002 | 40.2 | 41.4 | 0.678 |  |
| Density (+ cells/mm²) | -0.111 | 0.393 | 143.2 | 621.2 | < 0.001 | 207.4 | 245.9 | 0.512 |  |
| CD5^+^ cells - Whole tumor |  |  |  |  |  |  |  |  |  |
| Proportion (%) | 0.074 | 0.571 | 33.6 | 47.5 | 0.002 | 36.3 | 39.2 | 0.568 |  |
| Density (+ cells/mm²) | -0.147 | 0.256 | 93.3 | 391.7 | < 0.001 | 116.8 | 135.7 | 0.342 |  |
| CD8^+^ cells - Tumor center |  |  |  |  |  |  |  |  |  |
| Proportion (%) | 0.190 | 0.140 | 22.7 | 31.2 | 0.078 | 24.2 | 26.2 | 0.486 |  |
| Density (+ cells/mm²) | -0.101 | 0.436 | 67.8 | 184.6 | 0.001 | 69 | 82.2 | 0.590 |  |
| CD8^+^ cells - Invasive front |  |  |  |  |  |  |  |  |  |
| Proportion (%) | 0.212 | 0.098 | 21.3 | 28.8 | 0.012 | 22.4 | 26 | 0.223 |  |
| Density (+ cells/mm²) | -0.114 | 0.380 | 94 | 415.9 | < 0.001 | 108.1 | 166.2 | 0.571 |  |
| CD8^+^ cells - Whole tumor |  |  |  |  |  |  |  |  |  |
| Proportion (%) | 0.185 | 0.151 | 23.1 | 29.4 | 0.040 | 23.8 | 26.4 | 0.314 |  |
| Density (+ cells/mm²) | -0.146 | 0.260 | 79.1 | 266.1 | < 0.001 | 81.2 | 104.9 | 0.389 |  |
| CD20^+^ cells - Tumor center |  |  |  |  |  |  |  |  |  |
| Proportion (%) | -0.126 | 0.329 | 8 | 12.7 | 0.040 | 8.2 | 11.6 | 0.174 |  |
| Density (+ cells/mm²) | -0.116 | 0.371 | 8.9 | 69.5 | < 0.001 | 11.4 | 20.2 | 0.627 |  |
| CD20^+^ cells - Invasive front |  |  |  |  |  |  |  |  |  |
| Proportion (%) | -0.293 | 0.021 | 16.3 | 21.7 | 0.382 | 19.1 | 18.2 | 0.218 |  |
| Density (+ cells/mm²) | -0.232 | 0.070 | 47.1 | 230.4 | < 0.001 | 66.4 | 106.9 | 0.429 |  |
| CD20^+^ cells - Whole tumor |  |  |  |  |  |  |  |  |  |
| Proportion (%) | -0.276 | 0.029 | 12.8 | 21.1 | 0.344 | 12.8 | 15.9 | 0.137 |  |
| Density (+ cells/mm²) | -0.258 | 0.043 | 29.5 | 126.6 | < 0.001 | 35.9 | 49.3 | 0.265 |  |
| FOXP3^+^ cells - Tumor center |  |  |  |  |  |  |  |  |  |
| Proportion (%) | -0.160 | 0.219 | 9.3 | 11.1 | 0.078 | 10.9 | 9 | 0.891 |  |
| Density (+ cells/mm²) | -0.199 | 0.124 | 10 | 53.2 | < 0.001 | 12.3 | 24.6 | 0.761 |  |
| FOXP3^+^ cells - Invasive front |  |  |  |  |  |  |  |  |  |
| Proportion (%) | -0.058 | 0.658 | 4.6 | 6.4 | 0.055 | 6.2 | 5.3 | 0.891 |  |
| Density (+ cells/mm²) | -0.154 | 0.238 | 22.2 | 96.8 | < 0.001 | 24.6 | 33.9 | 0.651 |  |
| FOXP3^+^ cells - Whole tumor |  |  |  |  |  |  |  |  |  |
| Proportion (%) | -0.099 | 0.449 | 6.4 | 8 | 0.057 | 7.7 | 6.5 | 0.660 |  |
| Density (+ cells/mm²) | -0.197 | 0.128 | 14.1 | 67.6 | < 0.001 | 17 | 28.2 | 0.544 |  |
