# Supplementary material for: Age‐related remodelling of the blood immunological portrait and the local tumor immune response in patients with luminal breast cancer
Source: Clin Transl Immunology. 2020 Oct 3;9(10):e1184. doi: 10.1002/cti2.1184 (PMC7532981; doi:10.1002/cti2.1184)
Supplement: Supplementary file 9 [file CTI2-9-e1184-s009.docx]

# Supplementary methods

## BLOOD PROCESSING

Blood was collected in two 10 ml EDTA tubes (BD Vacutainer®; Becton, Dickinson and company, New Jersey), 4 ml of the total volume was used for EDTA plasma collection, 6 ml for T cell isolation the remaining blood was used for peripheral blood mononuclear cell (PBMC) isolation.

*Plasma collection*

Blood used for plasma collection was centrifuged at 1600 g for 15 minutes at 4 °C. After centrifugation, aliquots of EDTA plasma were stored at -80 °C.

PBMC isolation

PBMCs were isolated from fresh blood via density gradient centrifugation. An equal volume of PBS/2%FBS was added to the blood. 15 ml of Histopaque®-1077 (Sigma-Aldrich, Saint Louis) was transferred to a SepMate-50 tube (StemCell Technologies, Vancouver), afterwards the diluted blood sample was added as well. After centrifugation, the top layer (containing the enriched PBMCs) was poured off into a new tube and centrifuged again. The cell pellet was washed twice with PBS/2%FBS and resuspended in 2 ml of cold freezing medium and evenly divided over two 1.8-ml CryoTube vials and stored in liquid nitrogen.

T-cell isolation

After removal of plasma, an equal amount of PBS/2%FBS [Dulbecco’s phosphate-buffered saline (DPBS); Sigma-Aldrich, Saint Louis] containing 2% fetal bovine serum (FBS) (Invitrogen, Carlsbad) was added to the blood pellet. Afterwards the diluted blood was added to the 6 ml of blood for T cell isolation. RosetteSep Human T Cell Enrichment Cocktail (StemCell Technologies, Vancouver) was added to tube and incubated for 10 min at room temperature. Afterwards, an equal volume of PBS/2%FBS was added and mixed gently. 15 ml Histopaque®-1077 (Sigma-Aldrich, Saint Louis) was transferred to a SepMate-50 tube (StemCell Technologies, Vancouver), the diluted blood sample was added as well. After a centrifugation step, the top layer (containing the T cells) was poured off into a new tube and centrifuged again. The cell pellet was washed twice with PBS/2%FBS, resuspended in 1 ml of cold freezing medium [90% FBS with 10% dimethylsulfoxide (DMSO)] (Sigma-Aldrich, Saint Louis) and stored in liquid nitrogen.

## IMMUNE/SENESCENCE BIOMARKER ANALYSIS

*Plasma markers*

Following the manufacturer’s instructions, plasma levels of 11 pro- and anti-inflammatory cytokines and chemokines were analyzed by multiplex cytometric bead array technology (AimPlex Human Inflammation 11-plex; ImTec Diagnostics, Antwerp). These 11 inflammatory mediators were combined in a pre-mixed panel provided by ImTec Diagnostics and are often highly associated with inflammation. A serial dilution series of standards was used to determine the concentration of each analyte. All samples, standards and controls were assayed in duplicate. The data analysis was performed by using the FCAPArray software (BD Biosciences; Becton, Dickinson and company, New Jersey). The following plasma cytokines and chemokines were measured: tumor necrosis factor alpha (TNFα); interferon gamma-induced protein 10 (IP-10); interleukin (IL) 1 beta (IL-1β); IL-27; interferon gamma (INFγ); IL-8; IL-1α; IL-12p70; IL-6; monocyte chemoattractant protein 1 (MCP-1) and IL-10. In the same manner two additional Custom LEGENDplex^TM^ Assay panels (12-plex and 3-plex; BioLegend, San Diego) were run. Plasma cytokines IL-17A, IL-17F and Free active transforming growth factor beta 1 (TGF-β1) were measured, these are also associated with inflammation. Furthermore, the following immune checkpoint markers were assessed: soluble CD25 (sCD25); 4-1BB, CD86; cytotoxic T-lymphocyte-associated antigen 4 (CTLA-4); PD-L1; PD-1; T-cell immunoglobulin and mucin-domain containg-3 (TIM-3); lymphocyte-activation gene 3 (LAG-3) ; galectin-9 (Gal-9); soluble CD27 (sCD27); programmed cell death-ligand 2 (PD-L2) and also C-reactive protein (CRP). In a later stage, it will be checked if the immune checkpoint markers in plasma correlate with their expression in the tumor.

*IGF-1*

According to the manufacturer’s protocol, the plasma level of IGF-1 were measured with the Human IGF-I Quantikine ELISA kit (R&D Systems, Minneapolis). A serial dilution series of standards was included in order to obtain a standard curve, which was used to determine the concentration of IGF-1 in the samples. All samples, standards and controls were assayed in duplicate. Absorbance was measured at 450 nm and 570 nm. The readings at 570 nm were subtracted from the readings at 450 nm to correct for optical imperfections.

*p16^INK4a^*

RNA was extracted using the PicoPure® RNA Isolation Kit (Thermo Fisher Scientific, Waltham), from T-lymphocytes that were purified (up to >95% CD3^+^ cells) from PBMC with magnetic beads (EasySep™ Human T Cell Enrichment Kit; Stemcell Technologies, Vancouver) after a pre-incubation of 1 hour at 37 °C. 250 ng RNA was reverse transcribed using the SuperScript® III First-Strand Synthesis SuperMix (Invitrogen, Carlsbad) and cDNA was pre-amplified by using RealTime ready cDNA Pre-Amp Master (Roche Life Science, Penzberg). Via a probe-based real-time qPCR assay mRNA expression of *p16^INK4a^* was assessed, primers spanned the exon1α-exon2 boundary, to ensure that the assay is specific for *p16^INK4a^* without measuring the alternative transcript *p14^ARF^*. All procedures were performed following the manufacturer’s instructions and the samples were run in triplicate. The data was analyzed with the Qbase+ (Biogazelle, Zwijnaarde) using two reference house-keeping genes, Ribosomal Protein L13a (RPL13A) and Human 14-3-3 protein zeta/delta (YWHAZ) for normalization.

*mIRs*

Based on the literature a panel of miR was established, all miRs were linked to the immune response, inflammation and/or immunosenescence ^1-4^. miRs included in this panel are: let-7e, let-7i, miR-9, miR-17, miR-18a, miR-19a, miR-19b, miR-20a, miR-21, miR-92a, miR-125b, miR-126, miR-146a, miR-150, miR-155, miR-181a, miR-195, miR-223, miR-326 and miR-424. The miRs used for data normalization were miR-23a, miR-29a, miR-29c, miR-140 and miR-484; these have been validated as appropriate reference miRs for plasma samples in our previous miR study ^5^. miRs were isolated from plasma using the miRNeasy Serum/Plasma Advanced Kit (Qiagen, Hilden) according to the manufacturers protocol. During the isolation, spike-ins UniSp2; UniSp4 and UniSp5 from the RNA Spike-in-kit (Qiagen, Hilden) were added to evaluate the quality of the RNA isolation steps. To stabilize the RNA during the cDNA synthesis MS2 RNA (Roche *via* Sigma-Aldrich; Saint Louis) was also added to serve as a carrier. Isolated miRs were eluted with two times 25 µl DNase/RNase-free water and stored at -80°C. The isolated miR was reverse transcribed with the miRCURY LNA RT Kit (Qiagen, Hilden). To evaluate the cDNA synthesis two additional spike-ins UniSp6 from the miRCURY LNA RT Kit (Qiagen, Hilden) and cel-miR-39-3p from the RNA Spike-in kit (Qiagen, Hilden) were added during the procedure. The cDNA samples were stored at -80 °C until usage. Quality control was performed using the miRCURY LNA miRNA QC PCR Panel (Qiagen, Hilden) followed by miR measurement via RT-qPCR using miRCURY LNA miRNA Focus PCR Panels (Qiagen, Hilden) and miRCURY LNA SYBR® Green PCR Kit (Qiagen, Hilden). cDNA was assayed in 10 µl PCR reactions according to the manufacturers protocol and in triplicate. Once the PCR plate was loaded, the qPCR amplification was performed using the LightCycler® 480 II (Roche Life Science, Penzberg). The data was analyzed with the Qbase+ (Biogazelle, Zwijnaarde).

*PBMC subtyping*

The PBMC immune subset profiles were analyzed by 8-color flow cytometry, using a 3-laser FACSVerse platform (BD Biosciences; Becton, Dickinson and company, New Jersey). The use of appropriate fluorescent antibody panels targeted at specific cell surface markers allowed identification and frequency analysis of the different relevant immune cell subsets (Supplementary figure 1). Markers that are used were CD3 (T-lymphocytes); CD16 and CD56 (NK cells); CD14 and human leukocyte antigen - DR isotype (HLA-DR) (monocytes) with CD16 (to distinguish between classical, intermediate and non-classical monocytes); CD19 and HLA-DR (B-lymphocytes) with CD27 and immunoglobulin D (IgD) (to distinguish between naïve and memory B cells; HLA-DR with CD123 (plasmacytoid dendritic cell); HLA-DR with CD11c (myeloid dendritic cells); CD34 (stem cells). Within the T-cell population, further subtyping is carried out by looking at the following markers CD4^+^CD8^-^ (helper T-cells) *versus* CD4-CD8^+^ (cytotoxic T-cells); CD4^+^CD25^+^CD127^low^CD49d^-^ (Tregs); CD45RA^+^CCR7^+^ (naive) *versus* CD45RA^-^ C-C chemokine receptor type 7 (CCR7)^+/-^ (memory cells) *versus* CD45RA^+^CCR7^-^ (terminally differentiated effector cells). The co-stimulatory receptors CD27 and CD28 were additionally used to assess age-associated changes within the T-cell subsets as well as the senescence marker CD57. The following antibodies were purchased from BD Biosciences; Becton, Dickson and company, New Jersey: CD3-fluorescein isothiocyanate (FITC), CD4-phycoerythrin (PE).cyanine-7 (Cy7), CD8-allophycocyanin (APC).H7, CD11c-brilliant violet (BV)510, CD14-APC, CD16-PE.Cy7, CD19-APC.H7, CD27-APC.H7, CD56-BV421, HLA-DR-BV510 and HLA-DR-APC.H7. The remaining antibodies: CCR7-BV421, CD4-BV510, CD8-PE.Cy7, CD16-APC, CD25-BV421, CD27-PE, CD28-APC, CD34-PE.Cy7, CD45RA-BV510, CD45RA-PE.Cy7, CD49d-PE, CD57-PE CD123-PE, CD127-APC and IgD-BV421 were purchased from BioLegend, San Diego.

## TUMOR IMMUNE INFILTRATE ANALYSIS

*Tumor tissue*

The collected tumor tissue was formalin-fixed and paraffin-embedded (FFPE) after surgery. The FFPE tumor tissue sections were cut at a thickness of 5 µm.

*Characterization immune infiltrate*

The stromal tumor infiltrating lymphocytes (sTILs) were microscopically assessed on hematoxylin and eosin (H&E) stained representative tumor sections, according to published guidelines ^6^. To further characterize the tumor immune infiltrate a selection immune cell markers were evaluated. All markers are considered to play a role in the immune response in the tumor. T-cell markers CD3 and CD5, T-helper cell marker CD4 and the cytotoxic T-cell marker CD8 were chosen to evaluate the anti-cancer immune response in the tumor and their presences is associated with good prognosis however, the role of B-cells (CD20) in the tumor is more unknown ^7^. The tumor macrophages (CD68) are involved in many processes in the tumor microenvironment e.g. inflammation, response to treatment,… ^8^. FOXP3 was selected as a marker for Tregs, which are immunosuppressive T-cells that could impair the immune response in the tumor. The immunohistochemical stainings with the anti-CD3 (1:100, polyclonal, Dako; Agilent, Santa Clara), anti-CD5 (1:50, 4C7, Dako; Agilent, Santa Clara), anti-CD8 (1:100, C8/144, Dako; Agilent, Santa Clara), anti-CD20cy (1:400, L26, Dako); Agilent, Santa Clara), and anti-CD68 (1:100, PG-M1, Dako; Agilent, Santa Clara), immunostainings were performed manually. Using the Bond-Max autostainer (Leica Microsystems, Wetzlar) the immunohistochemical stainings of the anti-CD4 (1:50, 4B22, Dako; Agilent, Santa Clara) and anti-FOXP3 (1:100, 22510, Abcam; Cambridge) were carried out. The manual as well as the automated IHC procedure were carried out following the manufacturer’s instructions and can also be found in our methodological paper ^9^.

*CD68 staining grade*

The tumor macrophages were scored on an entire section stained for CD68 by assessing the stromal tissue between the tumor nests and at the invasive front, using a reported method ^10^. Grade I was assigned to the tumor sections with no or scanty macrophages in the tumor stroma; Grade II when small foci of macrophages were present in the tumor stroma; Grade III when large foci of macrophages were present in the tumor stroma and Grade IV when a diffuse dense infiltration of macrophages was observed in the tumor stroma. Where small foci represented aggregates of macrophages occupying less than half the surface area of a high power field (400x) and large foci represented aggregates of macrophages occupying more than half of the surface area of a high power field.

*QuPath analysis*

All immunohistochemical-stained slides were scanned using the Philips Ultra Fast Scanner version 1.6 (Philips, Andover), afterwards the scans were imported into Qupath for characterization of the immune infiltrate. The markers that were scored using this method were the T-cell markers CD3, CD4, CD5, CD8, CD20 and FOXP3. In different tumor regions, the *proportion* of positively stained lymphocytes was determined as a percentage by calculating the ratio positively stained lymphocytes and the total number of lymphocytes. The *density* of the positively stained lymphocytes was calculated as well by determining the number of positively stained lymphocytes per mm². A detailed description of the developed scoring system can be found methodological paper ^9^.

# **REFERENCES**

1. Olivieri F, Rippo MR, Monsurro V*, et al.* MicroRNAs linking inflamm-aging, cellular senescence and cancer. *Ageing Res Rev*. 2013.

2. Schickel R, Boyerinas B, Park SM, Peter ME. MicroRNAs: key players in the immune system, differentiation, tumorigenesis and cell death. *Oncogene*. 2008; **27**; 5959-5974.

3. Mogilyansky E, Rigoutsos I. The miR-17/92 cluster: a comprehensive update on its genomics, genetics, functions and increasingly important and numerous roles in health and disease. *Cell death and differentiation*. 2013; **20**; 1603-1614.

4. Schetter AJ, Heegaard NH, Harris CC. Inflammation and cancer: interweaving microRNA, free radical, cytokine and p53 pathways. *Carcinogenesis*. 2010; **31**; 37-49.

5. Hatse S, Brouwers B, Dalmasso B*, et al.* Circulating MicroRNAs as easy-to-measure aging biomarkers in older breast cancer patients: correlation with chronological age but not with fitness/frailty status. *PloS one*. 2014; **9**; e110644.

6. Salgado R, Denkert C, Demaria S*, et al.* The evaluation of tumor-infiltrating lymphocytes (TILs) in breast cancer: recommendations by an International TILs Working Group 2014. *Annals of Oncology*. 2015; **26**; 259-271.

7. Gonzalez H, Hagerling C, Werb Z. Roles of the immune system in cancer: from tumor initiation to metastatic progression. *Genes & development*. 2018; **32**; 1267-1284.

8. Yang M, McKay D, Pollard JW, Lewis CE. Diverse Functions of Macrophages in Different Tumor Microenvironments. *Cancer Research*. 2018; **78**; 5492-5503.

9. Berben L, Wildiers H, Marcelis L*, et al.* Computerised scoring protocol for identification and quantification of different immune cell populations in breast tumour regions by the use of QuPath software. *Histopathology*. **n/a**.

10. Ch'ng ES, Tuan Sharif SE, Jaafar H. In human invasive breast ductal carcinoma, tumor stromal macrophages and tumor nest macrophages have distinct relationships with clinicopathological parameters and tumor angiogenesis. *Virchows Archiv : an international journal of pathology*. 2013; **462**; 257-267.
