# Supplementary material for: Age‐related remodelling of the blood immunological portrait and the local tumor immune response in patients with luminal breast cancer
Source: Clin Transl Immunology. 2020 Oct 3;9(10):e1184. doi: 10.1002/cti2.1184 (PMC7532981; doi:10.1002/cti2.1184)
Supplement: Supplementary file 10 [file CTI2-9-e1184-s010.docx]

# Supplementary results

## Association of blood/tumor immune markers with pathological tumor characteristics

When looking at the breast tumor biology parameters (size, grade and nodal status), we found some interesting associations with the immune profile, mainly at the tumor level. Proportion of CD3^+^ cells in the tumor infiltrate positively correlated with tumor size (*P* = 0.002), whereas the opposite was true for CD20^+^ cells (*P* =0.029). Thus, the immune infiltrate in bigger tumors contains relatively more CD3^+^ and relatively less CD20^+^ cells as compared to tumors of smaller size. Moreover, grade III tumors showed significantly higher sTILs % (*P* < 0.001) and clearly were more heavily infiltrated by all immune subsets (*P* < 0.001 for all CD3^+^, CD4^+^, CD5^+^, CD8^+^, CD20^+^ and FOXP3^+^) than grade I-II tumors (Supplementary table 7). In contrast, no solid relationships could be demonstrated between blood immune/senescence markers and tumor size and grade, apart from some weak correlations of tumor grade with several specific CD4^+^ and CD8^+^ PBMC subsets. Lymph node involvement was not associated with any of the blood immune/senescence markers, nor with sTILs % or with any other tumor immune marker.
